# Effectiveness and safety of anti-BCMA chimeric antigen receptor T-cell treatment in relapsed/refractory multiple myeloma: a comprehensive review and meta-analysis of prospective clinical trials

**DOI:** 10.3389/fphar.2023.1149138

**Published:** 2023-06-20

**Authors:** Dingyuan Hu, Liming Chen, Diqin Yan, Wenliang Dong, Min Chen, Suping Niu, Simin Wang, Jiaojiao Zhang, Xiaoyan Nie, Yi Fang

**Affiliations:** ^1^ Clinical Trial Institution, Peking University People’s Hospital, Beijing, China; ^2^ Department of Clinical Pharmacy, School of Pharmaceutical Sciences, Peking University, Beijing, China; ^3^ Department of Science and Research, Peking University People’s Hospital, Beijing, China; ^4^ Department of Clinical Pharmacy, Xuzhou Medical University, Xuzhou, China

**Keywords:** chimeric antigen receptor T-cell treatment, BCMA, cancer immunotherapy, multiple myeloma, effectiveness, safety, meta-analysis

## Abstract

**Background:** Chimeric antigen receptor T cells treatment targeting B cell maturation antigen (BCMA) is an emerging treatment option for relapsed/refractory multiple myeloma (RRMM) and has demonstrated outstanding outcomes in clinical studies.

**Objective:** The aim of this comprehensive review and meta-analysis was to summarize the effectiveness and safety of anti-BCMA CAR-T treatment for patients with relapsed/refractory multiple myeloma (RRMM). Our research identifies variables influencing outcome measures to provide additional evidence for CAR-T product updates, clinical trial design, and clinical treatment guidance.

**Methods:** The Preferred Reporting Items for Systematic Reviews and Meta-Analyses (PRISMA) standard was followed for conducting this comprehensive review and meta-analysis, which was submitted to PROSPERO (CRD42023390037). From the inception of the study until 10 September 2022, PubMed, Web of Science, EMBASE, the Cochrane Library, CNKI, and WanFang databases were searched for eligible studies. Stata software (version 16.0) was used to assess effectiveness and safety outcomes.

**Results:** Out of 875 papers, we found 21 relevant trials with 761 patients diagnosed as RRMM and were given anti-BCMA CAR-T treatment. The overall response rate (ORR) for the entire sample was 87% (95% CI: 80–93%) complete response rate (CRR) was 44% (95% CI: 34–54%). The minimal residual disease (MRD) negativity rate within responders was 78% (95% CI: 65–89%). The combined incidence of cytokine release syndrome was 82% (95% CI: 72–91%) and neurotoxicity was 10% (95% CI: 5%–17%). The median progression-free survival (PFS) was 8.77 months (95% CI: 7.48–10.06), the median overall survival (OS) was 18.87 months (95% CI: 17.20–20.54) and the median duration of response (DOR) was 10.32 months (95% CI: 9.34–11.31).

**Conclusion:** According to this meta-analysis, RRMM patients who received anti-BCMA CAR-T treatment have demonstrated both effectiveness and safety. Subgroup analysis confirmed the anticipated inter-study heterogeneity and pinpointed potential factors contributing to safety and efficacy, which may help with the development of CAR-T cell studies and lead to optimized BCMA CAR-T-cell products.

**Systematic Review Registration:** Clinicaltrials.gov, PROSPERO, CRD42023390037.

## 1 Introduction

With a frequency of 6.5 per 100,000 people annually, multiple myeloma (MM) is the second most prevalent hematological malignancy after non-lymphoma (Lipe et al., 2016). During the past decades, RRMM remains an incurable condition despite considerable advancements in therapies, such as autologous stem cell transplantation (ASCT), new generations of proteasome inhibitors (PIs), immunomodulatory drugs (IMiDs), monoclonal antibodies (mAbs), bispecific antibodies (BsAbs) and antibody-drug conjugates (ADCs) (Manier et al., 2022). Compared with the methods of treatment mentioned above, the therapeutic effect of chimeric antigen receptor T-cell treatment appears to be more optimistic.

CAR-T cell therapy is a form of immunotherapy that modifies T cells with chimeric antigen receptors, which typically have an intracellular domain, a functional transmembrane domain, a target-recognition ectodomain, and a hinge region. The production of CAR-T cells can be obtained by transduction of T cells with either lentiviral or retroviral vectors carrying CAR-encoding genes or virus-free CRISPR CAR (VFC-CAR) (Mueller et al., 2022). The recombinant T cells occur to expand *in vitro* to produce tumor-specific chimeric antigen receptors (CAR). Additionally, by releasing soluble molecules that can alter stromal or immune cell function, CAR-T cells can also change the tumor microenvironment (Manier et al., 2022). The effectiveness of CAR-T treatment depends on the choice of targets. Currently, BCMA, CD19, CD22, CD138, SLAM7, and FHVH are targets of CAR-T cells investigated for RRMM (Sun et al., 2010; Drent et al., 2016; Schmidts et al., 2019; Lam et al., 2020; Golubovskaya et al., 2021; Luanpitpong et al., 2021; Zhao W H et al., 2022). When BCMA, also known as TNF receptor superfamily 17 (TNFRSF17), binds to its ligands (B cell activator of the TNF family [BAFF] and a proliferation-inducing ligand [April]), it releases pro-survival signaling those aids in the survival and growth of MM cells. Considering how strongly it exhibits its expression on the surface of malignant myeloma cells, BCMA is the most frequently chosen target (Madry et al., 1998). This article discusses the effectiveness and safety of anti-BCMA CAR-T products in the treatment of relapsed/refractory multiple myeloma. The strengths of our study are the large study population, the complete variety of anti-BCMA CAR-T products, the rich evaluation indicators, and the comprehensive subgroup analyses, which provide an evidence-based reference for the development of a new generation of CAR-T treatment regimens and optimization of the structure. It is important for the clinical application of BCMA-targeted CAR-T therapy in the treatment of relapsed/refractory multiple myeloma.

## 2 Materials and methods

### 2.1 Methods

The procedures used in this comprehensive review and meta-analysis adhered to the Preferred Reporting Items for Systematic Reviews and Meta Analyses (PRISMA) criteria and were recorded on PROSPERO (CRD42023390037) (Knobloch et al., 2011).

### 2.2 Search strategy

The benefits and risks of anti-BCMA CAR-T cell treatment in RRMM are being thoroughly reviewed and meta-analyzed in this study. From the start of the study through 10 September 2022, without regard to language, relevant clinical studies were meticulously searched and screened by PubMed, Web of Science, EMBASE, the Cochrane Library, CNKI, and WanFang databases. We merged Medical Subject Headings (Mesh) phrases with free-text terms including (“B-cell maturation antigen” OR “BCMA”) AND (“chimeric antigen receptor” OR “CAR”) AND “multiple myeloma” to search for eligible studies. The Supplementary Materials provide a thorough search methodology. Additionally, we looked through PROSPERO for any pertinent systematic reviews.

### 2.3 Inclusion and exclusion criteria

The following studies were eligible for inclusion in this investigation:

1) Study type: Prospective single-arm studies that could be single-center or multicenter, phase 1 or phase 2, were eligible for inclusion. Chinese Clinical Trial Registry (ChiCTR number) or Clinicaltrials.gov (NCT number) clinical studies were taken into consideration.
2) Participants: Patients with relapsed or refractory multiple myeloma were included.
3) Intervention: Patients who received anti-BCMA CAR T-cell treatment were included.
4) Results: At least one of the effectiveness outcomes and one of the safety outcomes were reported by qualified studies. Efficacy outcome measures include the overall response rate (ORR), complete response rate (CRR), minimal residual disease (MRD) negativity within responders who obtained VGPR or better, progression-free survival (PFS), overall survival (OS) and duration of Response (DOR). ORR included strict complete response (sCR), complete response (CR), very good partial response (VGPR), and partial response (PR). CRR included sCR and CR. Safety outcome measures include any grade cytokine-release syndrome (CRS), grade≥3 CRS, CAR-T-related encephalopathy syndrome (CRES), grade≥3 CRES, hematologic toxicity (neutropenia, anemia, thrombocytopenia, leukopenia, lymphopenia), relapse, infection, and fever. (Gagelmann et al., 2020; Roex et al., 2020; Xiang et al., 2020; Yang et al., 2021; Zhang et al., 2021).

Studies that match the following criteria were excluded:

1) Combined use of other treatments;
2) Received dual-target or multiple-target CAR-T treatment;
3) Case reports, observational studies, conference presentations, abstracts, editorials and review articles;
4) Similar and repeated studies.

### 2.4 Data extraction

The following data was reviewed and extracted by two authors independently: study characteristics, patient characteristics, anti-BCMA CAR-T treatment characteristics, and outcome measures. Any differences of opinion were settled by debate until an agreement was achieved or by contacting a third author.

### 2.5 Assessment of study quality and publication bias

To rate the methodological excellence of the included studies, the Methodological Index for Non-randomized Studies (MINORS) was employed. (Slim et al., 2003). All of the studies lacked a control group, scores for non-randomized trials were calculated using only eight methodological elements. Each research could receive a maximum score of 16, with the items being evaluated as 0 (not known), 1 (known but insufficient), or 2 (known and adequate). Confirmation of exposure bias was obtained with Egger’s and Begg’s test and analyzed using funnel plots.

### 2.6 Statistical analysis

Stata software (version 16.0) was utilized to conduct this meta-analysis. We used the extracted data for response and incidence rates of adverse events to conduct meta-analyses on each outcome. All results were presented together with their corresponding 95% confidence intervals (CI). The chi-squared test (χ^2^ test) and the I-squared test (I^2^ test) were used to assess the heterogeneity among studies. Analysis was carried out using the random-effect model if there was any chance of heterogeneity (I^2^> 50%); while the fixed-effect model was used in all other cases.

Preplanned subgroup analysis was conducted according to age (<55 vs. ≥55 years), dose (high dose group ≥200 × 10^6cells or 5 × 10^6 cells/kg vs. low dose group <200 × 10^6cells or 5 × 10^6 cells/kg), antigen-recognition domain origin (Human vs. Murine vs. Llama), costimulatory molecule (4-1BB vs. others), loading (Lentiviral vs. Retrovirus), the median time from diagnosis (<4 vs. ≥4 years), lines of prior treatment (<8 vs. ≥8), percentage of previous ASCT (<75% vs. ≥75%), percentage of high-risk cytogenetics (<48% vs. ≥48%), percentage of extramedullary disease (<29% vs. ≥29%), the proportion of ECOG≥3 level (<25% vs. ≥25%), the proportion of ISS≥3 level (<28% vs. ≥28%), the proportion of mAb exposed (<39% vs. ≥39%) to investigate the sources of heterogeneity. Statistics were considered significant for P values under 0.05. Sensitivity analysis was used to calculate the effect when the included study with the greatest sample size was excluded.

## 3 Results

### 3.1 Study selection and characteristics

**FIGURE 1:**
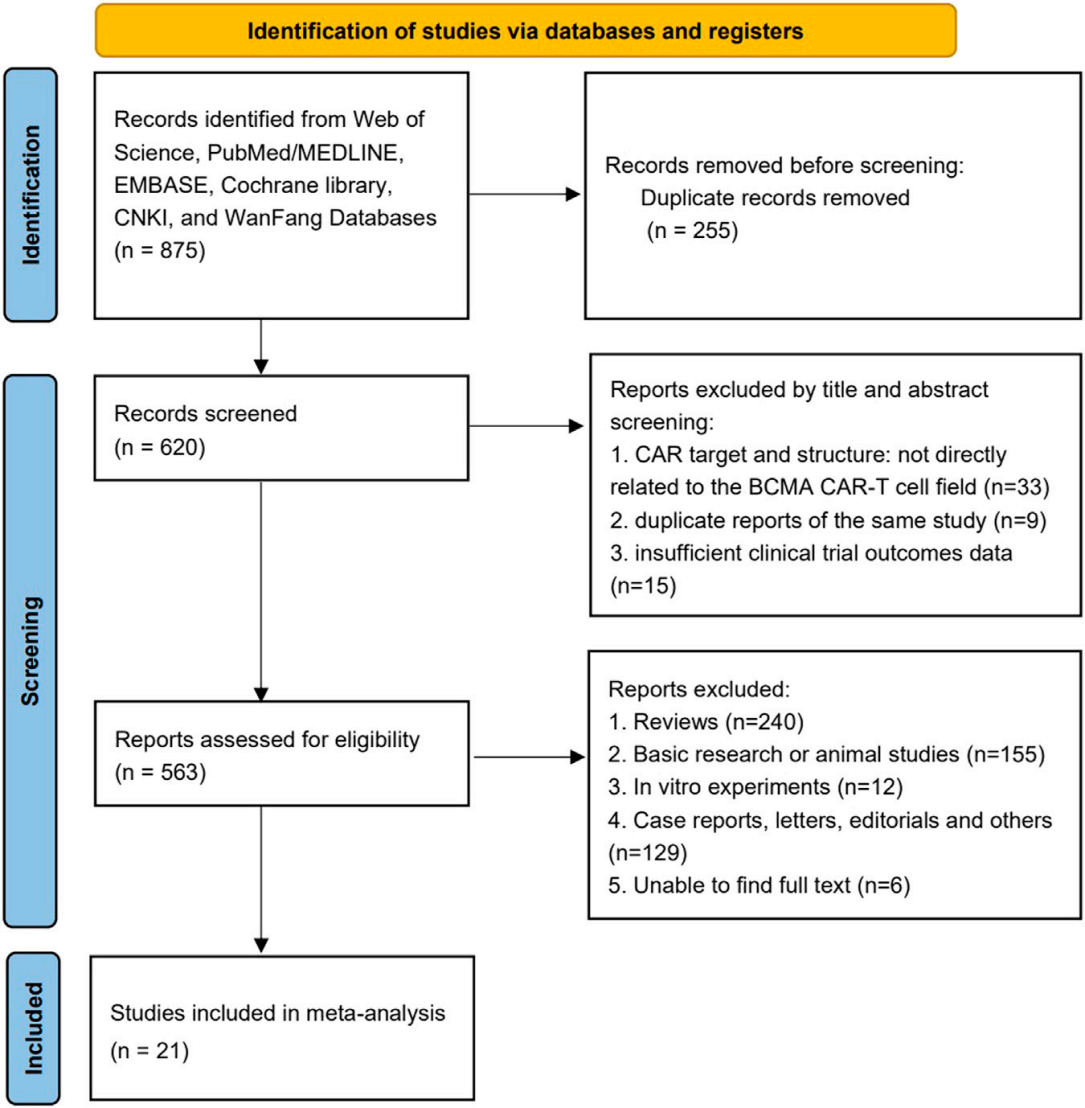
PRISMA flow diagram for the record selection process.

The PRISMA flow diagram depicts the search method used to identify the pertinent publications (Figure 1). Through the comprehensive search of six databases, 875 records were found overall. Considering the title and abstract, we omitted 542 items after deleting 255 duplicates. The whole text of the remaining 78 possibly pertinent papers was examined. Only the most recent findings for the identical study were shown. After a detailed evaluation of these reports, 21 studies enrolling a total of 761 participants, were considered for analysis (Alsina et al., 2020; Berdeja et al., 2021; Brudno et al., 2018; Chen et al., 2020; C. L. Costello et al., 2020; Wang et al., 2021; Du et al., 2022; Frigault et al., 2020; Green et al., 2018; Hao et al., 2020; Hu et al., 2019; S. K. Kumar et al., 2020; Li et al., 2021; Lin et al., 2020; Mailankody, Ghosh, et al., 2018; Mailankody, Htut, et al., 2018; Manjunath et al., 2021; Munshi et al., 2021; Qu et al., 2022; Xiao-Yuan et al., 2022; Zhao A et al., 2022). The characteristics of the included studies and the enrolled patients who were diagnosed as RRMM and treated with BCMA CAR-T therapy were displayed in Tables 1, 2, respectively.

**TABLE 1:** Characteristics of included studies.

No	Study	Year	Registration number	Production name	Design	No. of patient	Conditioning	CAR-T infused dose	Antigen-recognition domain	Costimulatory molecule	Loading	Distinctive features	T cell origin	Follow up
1	Lin Y	2020	NCT02658929	Idecabtagene Vicleucel (bb2121)	2-part, phase 1, dose escalation and expansion	62	CP300 mg/m^2^ + Flu30 mg/m^2^ daily for 2d	50 × 10^6cells (3)	A murine anti-BCMA ScFv	4-1BB	__	__	__	14.7month
								150 × 10^6cells (18)						
								450× 10^6^^6cells (38)						
								800 × 10^6cells (3)						
2	Jennifer N. Brudno	2018	NCT02215967	Anti-BCMA CAR-T cells	Phase I, single arm	16	CP300 mg/m^2^ +Flu30 mg/m^2^ daily for 3d	9 × 10^6 cells/kg	A murine anti-BCMA ScFv	CD28	Retrovirus	__	__	__
3	Hao S	2020	NCT03716856, NCT03302403, NCT03380039	Zevorcabtagene autoleucel (CT053)	3-Site, phase I, single-arm, open-label	24	CP1610 mg + Flu108 mg daily for 2-4d	150 × 10^6cells (21)	A fully human anti-BCMA ScFv (25C2)	4-1BB	__	__	Autologous	24 month.
								50 × 10^6cells (1)						
								100 × 10^6cells (1)						
								180 × 10^6cells (1)						
4	Alsina M	2020	NCT03274219	bb21217	Multi-center, phase 1, dose escalation and expansion	46	CP300 mg/m^2^ + Flu30 mg/m^2^ daily for 3d	150 × 10^6cells (12)	A murine anti-BCMA ScFv	4-1BB	__	the PI3K inhibitor bb007	__	8.5 month
								300 × 10^6cells (14)						
								450 × 10^6cells (20)						
5	Wan-Hong Zhao	2022	NCT03090659 ChiCTR-ONH-17012285	Ciltacabtagene autoleucel (JNJ-68284528 LCAR-B38M)	Multicenter, phase 1, single-arm, open-label	74	CP300 mg/m^2^ or CP250 mg/m^2^ + Flu20 mg/m^2^	0.513 × 10^6 cells/kg	Two llama-derived heavy-chain-based anti-BCMA single-domain antibodies	4-1BB	Lentiviral	__	Autologous	47.8 month
6	Sham Mailankody	2018a	NCT03430011	Orvacabtagene autoleucel (JCARH125)	Multisite phase1/2, single arm	8	CP300 mg/m^2^ + Flu30 mg/m^2^ daily for 3d	50 × 10^6 cells (5)	A fully human anti-BCMA ScFv	4-1BB	Lentiviral	__	__	5 weeks
								150 × 10^6 cells (3)						
7	Kumar SK	2020	NCT03915184	Zevorcabtagene autoleucel (CT053)	Multisite, Phase 1b/2, single arm	14	CP500 mg/m^2^/d*2d + Flu25 mg/m^2^/d*3d	150–180 × 10^6cells (8)	A fully human anti-BCMA ScFv (25C2)	4-1BB	__	__	Autologous	4.5 month
								250–300× 10^6cells (6)						
8	Sham Mailankody	2018b	NCT03070327	EGFRt/BCMA-41BBz CAR T cells (MCARH171)	Phase I, single arm	11	Cy3mg/m^2^ or CP/Flu: CP300 mg/m^2^ + Flu30 mg/m^2^ daily for 3d	72–137×10^6 cells (6)	A human anti-BCMA ScFv	4-1BB	__	a truncated epidermal growth factor receptor safety system	Autologous	__
								475–818×10^6 cells (5)						
9	Di Wang	2021	ChiCTR1800018137	CT103A	Phase 1, open-label, single-arm, dose escalation, and expansion	18	CP20 mg/m^2^ + Flu25 mg/m^2^ for 3d	1 × 10^6 cells/kg (9)	A fully human anti-BCMA ScFv	4-1BB	__	__	Autologous	394 days
								3 × 10^6 cells/kg (6)						
								6 × 10^6 cells/kg (3)						
10	Shwetha H. Manjunath	2021	NCT02546167	CART-BCMA	Phase I, single arm	25	CP or none	10–50 × 10^6 cells (8)	A fully human anti-BCMA ScFv	4-1BB	__	__	Autologous	16.3 month
								100–500×10^6cells (17)						
11	Damian J. Green	2018	NCT03338972	Anti-BCMA CAR-expressing CD4+/CD8+ T-lymphocytes (FCARH143)	Phase I, single arm	7	Null	50 × 10^6 cells (5)	A fully human anti-BCMA ScFv	4-1BB	Lentiviral	The CD8^+^ and CD4^+^ T cells were stimulated with anti-CD3/antiCD28 paramagnetic beads	Autologous	__
								150 × 10^6 cells (2)						
12	Jesus G Berdeja MD	2021	NCT03548207	Ciltacabtagene autoleucel (JNJ-68284528 LCAR-B38M)	Phase 1b/2	97	CP300 mg/m^2^ + Flu30 mg/m^2^ daily for 3d	0.75 × 10^6cells/kg	Two llama-derived heavy-chain-based anti-BCMA single-domain antibodies	4-1BB	__	__	__	8.8 month
13	Nikhil C. Munshi	2022	NCT03361748	Idecabtagene Vicleucel (bb2121)	Multicenter, phase 2, single-arm, open-label	128	CP300 mg/m^2^+Flu30 mg/m^2^ daily for 3d	150 × 10^6 cells (4)	A murine anti-BCMA ScFv	4-1BB	Lentiviral	__	__	13.3 month
								300 × 10^6 cells (70)						
								450 × 10^6 cells (54)						
14	Xiaoyuan, Zhang	2022	__	BCMA-CART cells	Phase I, single arm	21	CP1g/m^2^ for 5d + Flu20-25 mg/m^2^ daily for 3d	4.2 × 10^6 cells/kg	A fully human anti-BCMA ScFv (14)	4-1BB (14)4-1BB/CD28 (7)	__	__	Autologous	19.3 month
									A murine anti-BCMA ScFv (7)					
15	Juan Du	2022	NCT03093168	HDS269B	Open-label, single-arm, phase I/II	49	CP300 mg/m^2^ +Flu30 mg/m^2^ daily for 3d	9 × 10^6 cells/kg	A murine anti-BCMA ScFv	4-1BB/CD137	Retrovirus	__	Autologous	14 month
16	Xiaoyan Qu	2022	NCT04322292 NCT03815383 NCT03751293 NCT04295018	C-CAR088	Multi-center, single-arm, open-label, phase 1, dose escalation and expansion	31	CP300 mg/m^2^ + Flu30 mg/m^2^ daily for 3d	1 × 10^6 cells/kg (4)	A human IgG1 antibody anti-BCMA ScFv	4-1BB	Lentiviral	__	Autologous	9.5 month
								3 × 10^6 cells/kg (13)						
								6 × 10^6 cells/kg (14)						
17	Chen W	2020	NCT03975907	Zevorcabtagene autoleucel (CT053)	Phase 1, dose escalation and expansion	14	CP300 mg/m^2^ +Flu25 mg/m^2^ daily for 3d	100 × 10^6cells (3)	A fully human anti-BCMA ScFv (25C2)	4-1BB	__	__	Autologous	5 month
								150 × 10^6cells (11)						
18	Costello CL	2020	NCT03288493	P-BCMA-101	Phase 1/2	43	CP300 mg/m^2^ + Flu30 mg/m^2^ daily for 3d	0.75 × 10^6 cells/kg	A fully human anti-BCMA ScFv	4-1BB	PiggyBac	P-BCMA-101 cells comprise a high percentage of Tscm cells and carry a selection gene and a “safety switch” gene.	Autologous	__
19	Frigault MJ	2020	NCT04155749	CART-Ddbcma	Phase 1, multicenter, open-label, dose-escalation	10	CP/Flu	100 × 10^6 cells (6)	a non-human, non-immunoglobulin–derived BCMA-binding domain discovered from D domain phage display libraries	4-1BB	__	__	__	208 days
								300 × 10^6 cells (4)						
20	Hu Y	2019	ChiCTR-1800017404	BCMA CAR-T	Phase 1, single-arm, open-label, single center	33	CP/Flu	3.5 (1–6) ×10^6 cells/kg	__	4-1BB	Lentiviral	__	Autologous	8 month
21	Chunrui Li	2021	ChiCTR-OPC-16009113	BCMA CAR-T	Phase I, single arm	30	CP20 mg/m^2^ + Flu25 mg/m^2^ daily for 3d	11.2 × 10^6 cells/kg	A murine anti-BCMA ScFv	CD28	Lentiviral	__	Autologous	385 days

**TABLE 2:** Characteristics of included patients.

No	Study	Male/Female	Mean age (years)	Median time from diagnosis (years)	Lines of prior treatment	Prior ASCT (%)	High-risk cytogenetics (%)	Extramedullary disease (%)	ECOG≥3 level (%)	ISS ≥3 level (%)	mAb exposed (%)	BCMA positivity requirement at enrollment (%)
1	Lin Y	—	61	5	6	97	45	27	—	—	90	—
2	Jennifer N. Brudno	—	—	—	9.5	75	40	—	—	—	44	—
3	Hao S	13/11	60.1	3.5	4.5	42	38	42	33	38	21	91.3
4	Alsina M	—	62	—	6	85	40	—	—	—	72	—
5	Wan-Hong Zhao	45/29	54.5	4	3	24	36	30	16	28	—	—
6	Sham Mailankody	—	53	4	10	88	50	—	—	—	—	—
7	Kumar SK	—	59	3.9	6	57	64	36	—	—	—	—
8	Sham Mailankody	—	—	—	6	—	82	—	—	—	9	—
9	Di Wang	10/8	53.5	2.6	4	33	39	—	—	0	39	83
10	Shwetha H. Manjunath	16/9	58	4.6	7	92	96	28	—	—	76	—
11	Damian J. Green	—	63	—	8	71	71	—	—	—	—	—
12	Jesus G Berdeja MD	57/40	61	5.9	6	90	24	—	4	14	84	>50% (57/62)
13	Nikhil C. Munshi	76/52	61	6	6	78	78	78	67	76	—	>50% (85/109)
14	XiaoYuan, Zhang	12/9	55	—	5	38	86	24	—	38	—	—
15	Juan Du	26/23	57	2.7	4	28	43	22	41	27	9	46
16	Xiaoyan Qu	17/14	61	—	4	23	48	10	0	16	23	49
17	Chen W	—	54	—	6	71	36	14	—	—	—	—
18	Costello CL	29/14	60	—	7	58	—	—	—	—	—	—
19	Frigault MJ	—	66	—	5	—	89	67	—	—	—	—
20	Hu Y	—	62.5	—	—	—	—	—	—	—	—	—
21	Chunrui Li	17/13	55	3.7	4	37	80	47	—	29	13	93

Abbreviations: ASCT, autologous stem cell transplant; ECOG, eastern cooperative oncology group; ISS, international staging system.

### 3.2 Evaluation of study quality and bias risk

**FIGURE 2:**
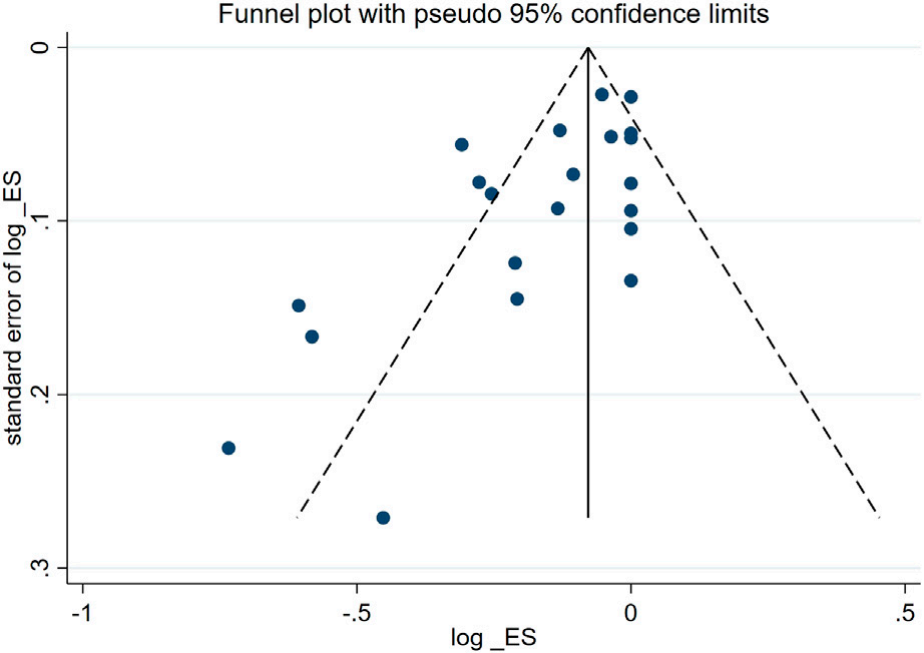
The funnel diagram of included studies.

The median MINORS score was 13 for the twenty-one non-comparative studies (ranging from 6 to 16). Evaluation results demonstrated the high quality of the included research (Table 3). According to the sensitivity analysis of ORR, the effect size of the outcome index did not change considerably after any of the studies were excluded. As shown in the funnel diagram no evidence of potential publication bias was revealed for the overall response, proving that the findings of our meta-analysis were robust and consistent. (Figure 2). The studies with a significant risk of bias were left in due to the limited number of included research.

**TABLE 3:** The scores of MINORS.

No	Study	Clearly stated aim	Inclusion of consecutive patients	Prospective collection of data	Endpoints appropriate to the aim of the study	Unbiased assessment of the study endpoint	Follow-up period appropriate to the aim of the study	Loss to follow up less than 5%	Prospective calculation of the study size	MINORS score
1	Lin Y	2	0	2	2	2	2	2	0	12
2	Jennifer N. Brudno	2	2	2	2	2	2	2	2	16
3	Hao S	2	0	2	2	2	2	2	0	12
4	Alsina M	2	2	2	2	1	2	2	0	13
5	Wan-Hong Zhao	2	2	2	2	2	2	2	0	14
6	Sham Mailankody	2	0	2	2	0	0	0	0	6
7	Kumar SK	2	2	2	2	2	0	2	0	12
8	Sham Mailankody	2	0	2	2	2	0	2	0	10
9	Di Wang	2	2	2	2	2	0	2	0	12
10	Shwetha H. Manjunath	2	2	2	2	0	2	2	0	12
11	Damian J. Green	2	0	2	2	2	0	2	0	10
12	Jesus G Berdeja MD	2	2	2	2	2	1	2	0	13
13	Nikhil C. Munshi	2	2	2	2	2	2	2	0	14
14	XiaoYuan, Zhang	2	2	2	2	2	2	2	2	16
15	Juan Du	2	2	2	1	2	2	2	2	15
16	Xiaoyan Qu	2	2	2	2	2	2	2	0	14
17	Chen W	2	2	2	2	2	2	2	0	14
18	Costello CL	2	2	2	1	1	2	2	0	12
19	Frigault MJ	2	2	2	2	2	2	2	0	14
20	Hu Y	2	2	2	1	1	2	2	2	14
21	Chunrui Li	2	2	2	2	2	2	2	2	16

### 3.3 Effectiveness outcomes

**FIGURE 3:**
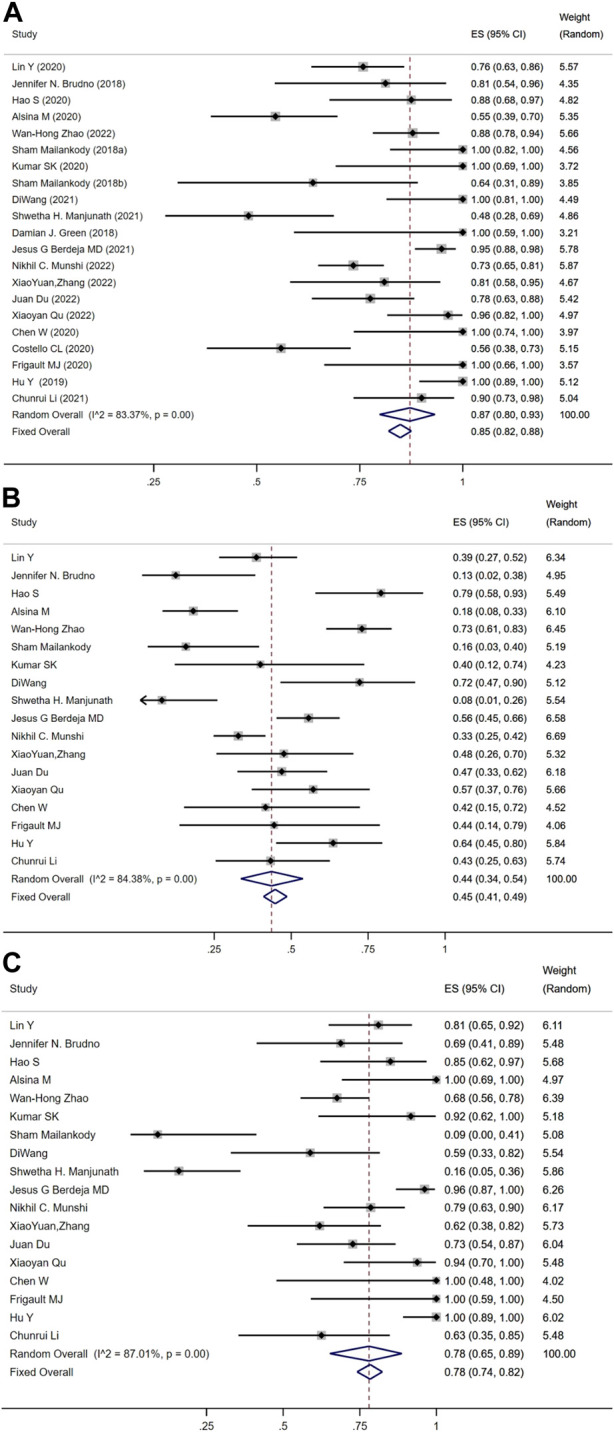
Pooled rate of **(A)** objective response, **(B)** complete response, and **(C)** minimal residual disease negativity among RRMM.

The meta-analysis based on twenty-one included studies that evaluated the rate of favorable outcomes to anti-BCMA CAR-T-cell treatment in RRMM patients. ORR was reported in 751 patients from 21 studies, and the combined ORR was 87% (95% CI: 80%–93%; Figure 3A). CRR was reported in 699 patients from 18 studies, and the pooled CRR was 44% (95% CI: 34%–54%; Figure 3B). The combined MRD negativity rate among responders was 78% (95% CI: 65%–89%) among eighteen trials that assessed the minimal residual disease (Figure 3C). There were 28% (95% CI: 17%–41%), 28% (95% CI: 13%–44%), 23% (95% CI: 17%–30%), and 18% (95% CI: 13%–24%) for the sCR, CR, VGPR, and PR, respectively. The median progression-free survival (PFS) was 8.77 months (95% CI: 7.48–10.06), the median overall survival (OS) was 18.87 months (95% CI: 17.20–20.54) and the median duration of response (DOR) was 10.32 months (95% CI: 9.34–11.31).

### 3.4 Safety outcomes

**FIGURE 4:**
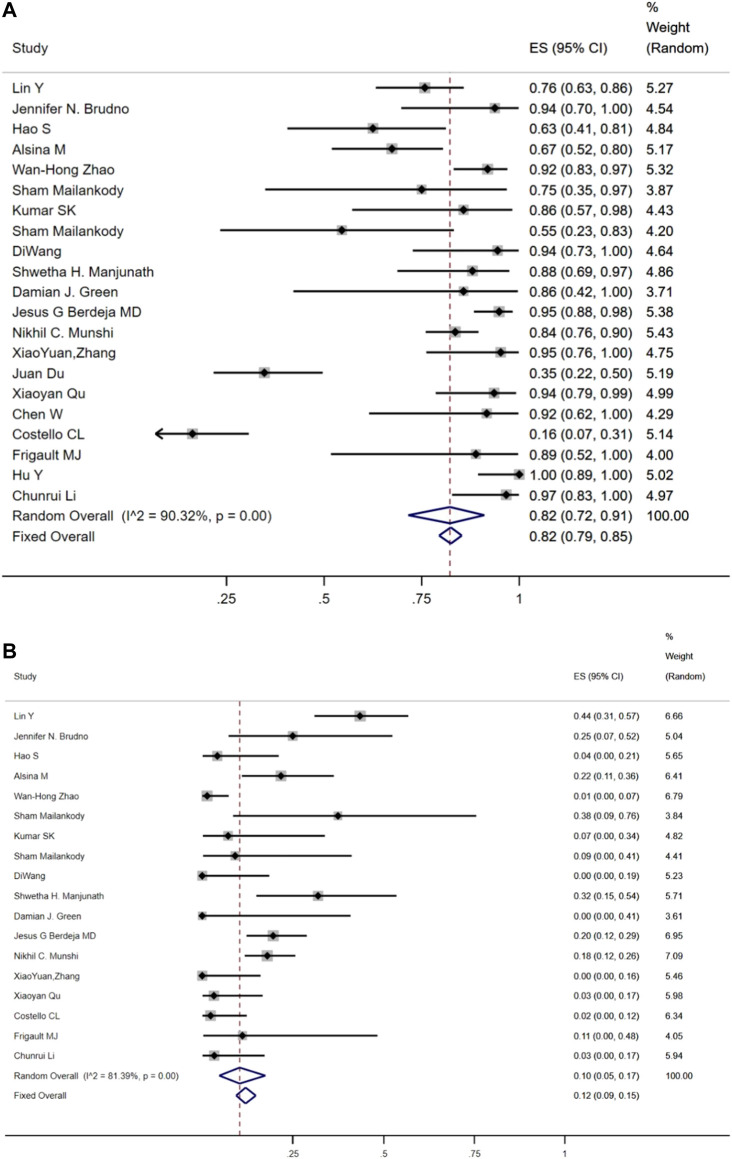
The pooled rate of **(A)** cytokine release syndrome (CRS) and **(B)** CAR-T-related encephalopathy syndrome (CRES) among RRMM.

The safety of anti-BCMA CAR-T-cell treatment in RRMM patients was assessed in this meta-analysis. CRS was the most commonly reported adverse event (AE). Twenty-one studies reported any CRS grade. Figure 4A shows that the overall incidence of CRS was 82% (95% CI: 72%–91%), and the combined incidence of grade≥3 CRS was 11% (95% CI: 6%–17%). Twelve studies reported the use of tocilizumab for CRS treatment and the pooled usage rate of tocilizumab was 46% (95% CI: 33%–59%). Any grade CRES was recorded in eighteen studies. Figure 4B shows that the cumulative incidence of CRES was 10% (95% CI: 5%–17%), while the cumulative incidence of grade≥3 CRES was 2% (95% CI: 0%–5%). The most frequent grade≥3 adverse events (AE) associated with CAR-T treatment was hematologic toxicity, which included neutropenia (86%, 95% CI: 76%–94%), anemia (66%, 95% CI: 50%–81%), thrombocytopenia (62%, 95% CI: 49%–75%), leukopenia (83%, 95% CI: 66%–96%) and lymphopenia (70%, 95% CI: 45%–90%). The pooled incidences of infection and fever were 39% (95% CI: 21%–58%) and 70% (95% CI: 40%–93%), respectively.

### 3.5 Recurrence outcomes

**FIGURE 5:**
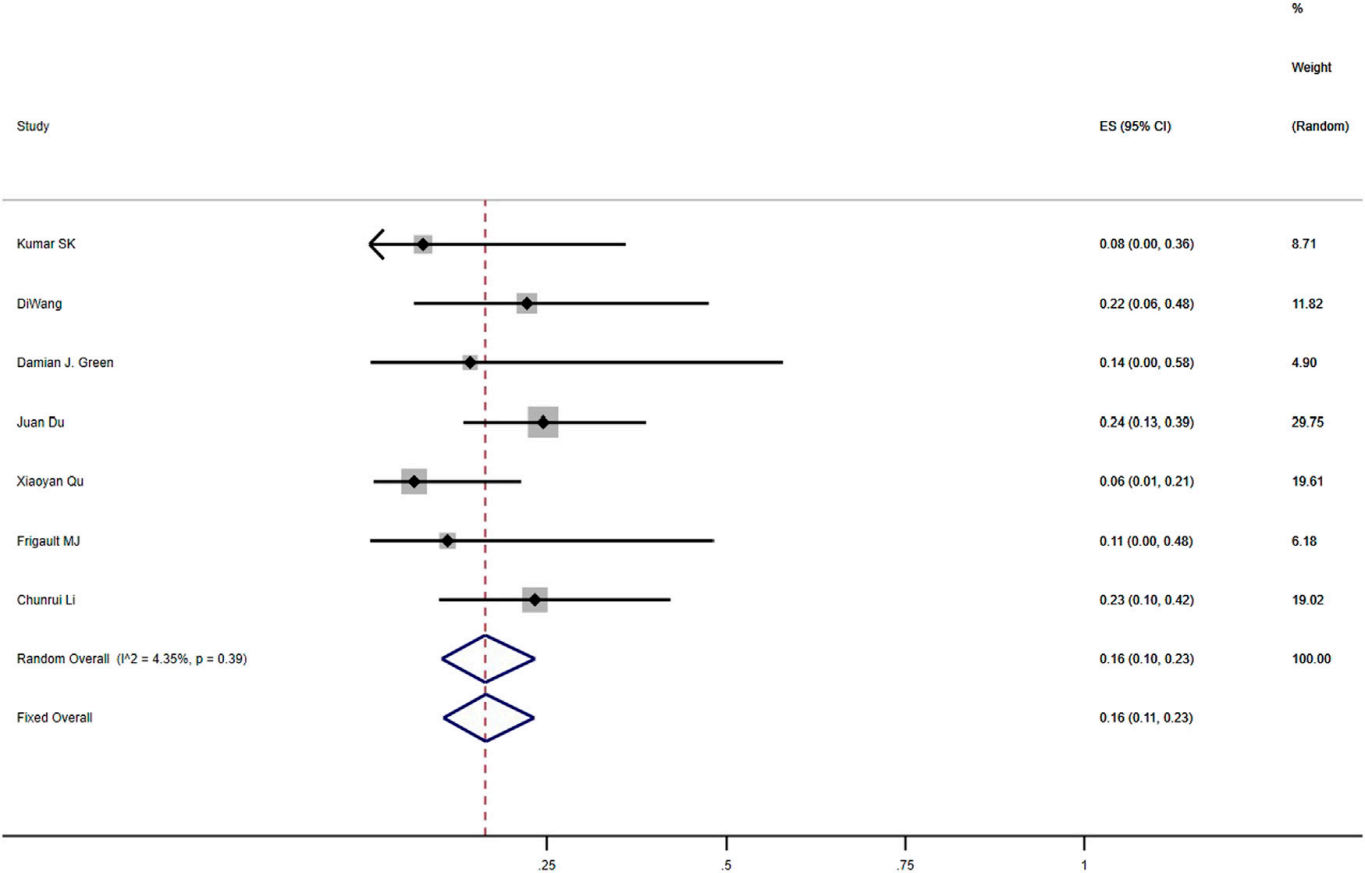
The pooled rate of recurrence within 1 year among RRMM.

The meta-analysis based on seven included studies evaluated the recurrence rate of anti-BCMA CAR-T-cell treatment in patients with RRMM (Green et al., 2018; Frigault et al., 2020; Kumar et al., 2020; Li et al., 2021; Wang et al., 2021; Du et al., 2022; Qu et al., 2022). The pooled recurrence rate within 1 year was 16% (95% CI: 10%–23%; Figure 5).

### 3.6 Subgroup analysis outcomes

We used subgroup analysis to investigate the pertinent factors that possibly affect the effectiveness and safety of anti-BCMA CAR-T-cell treatment in patients with RRMM including mean age of patients, CAR-T cell infusion dosage, CAR structures (antigen-recognition domain origin, costimulatory molecule, loading), midpoint from diagnosis, lines of prior therapy, prior ASCT (%), high-risk cytogenetics (%), extramedullary disease (%), the proportion of patients with ECOG score ≥3 level (%), the proportion of patients with ISS score ≥3 level (%) and the proportion of patients with mAb exposed (%). The results are shown in Table 4.

**TABLE 4:** Subgroup analysis results of overall response and cytokine-release syndrome rate.

Subgroups	Overall response rate			Cytokine-release syndrome rate		
	No. Of trials	ORR (95% CI)	P for difference	No. Of trials	CRS (95% CI)	P for difference
Mean age (years)			0.016			0.074
≥55	15	0.84 (0.78; 0.92)		15	0.83 (0.77; 0.91)	
<55	4	0.96 (0.90; 1.03)		4	0.92 (0.86; 0.98)	
Dose			0.045			—
high dose group ≥200 × 10^6cells or 5 × 10^6 cells/kg	12	0.82 (0.75; 0.90)		—	—	
low dose group <200 × 10^6cells or 5 × 10^6 cells/kg	18	0.92 (0.87; 0.98)		—	—	
Antigen-recognition domain origin			0.010			0.018
Human	11	0.91 (0.83; 0.99)		11	0.83 (0.73; 0.94)	
Murine	6	0.76 (0.69; 0.85)		6	0.77 (0.66; 0.90)	
Llama	2	0.92 (0.86; 0.99)		2	0.94 (0.90; 0.98)	
Costimulatory molecule			0.351			0.677
4-1BB	17	0.88 (0.82; 0.94)		17	0.86 (0.80; 0.92)	
others	4	0.84 (0.76; 0.92)		4	0.82 (0.65; 1.02)	
Loading			0.066			0.347
Lentiviral	7	0.92 (0.84; 1.00)		7	0.93 [0.87; 0.99]	
Retrovirus	2	0.78 ((0.68; 0.91)		2	0.58 (0.22; 1.54)	
Median time from diagnosis (years)			0.263			0.220
≥4	6	0.84 (0.75; 0.94)		6	0.88 (0.82; 0.94)	
<4	5	0.91 (0.83; 1.00)		5	0.75 (0.58; 0.96)	
Lines of prior treatment			0.011			0.369
≥8	3	0.98 (0.90; 1.07)		3	0.91 (0.77; 1.06)	
<8	17	0.85 (0.79; 0.91)		17	0.84 (0.78; 0.90)	
Prior ASCT (%)			0.068			0.775
≥75	7	0.78 (0.68; 0.90)		7	0.85 (0.77; 0.93)	
<75	11	0.90 (0.85; 0.97)		11	0.83 (0.73; 0.93)	
High-risk cytogenetics (%)			0.783			0.116
≥48	10	0.88 (0.80; 0.98)		10	0.90 (0.86; 0.95)	
<48	9	0.86 (0.79; 0.94)		9	0.82 (0.73; 0.91)	
Extramedullary disease (%)			0.489			0.360
≥29	6	0.88 (0.80; 0.97)		6	0.88 (0.82; 0.96)	
<29	6	0.83 (0.72; 0.95)		6	0.81 (0.69; 0.96)	
ECOG≥3 level (%)			0.001			0.06
≥25	3	0.78 (0.70; 0.86)		3	0.58 (0.36; 0.96)	
<25	3	0.94 (0.89; 0.98)		3	0.94 (0.90; 0.98)	
ISS≥3 level (%)			0.046			0.481
≥28	5	0.84 (0.77; 0.91)		5	0.90 (0.83; 0.97)	
<28	4	0.94 (0.87; 1.01)		4	0.83 (0.70; 1.00)	
mAb exposed (%)			0.263			0.143
≥39	6	0.79 (0.68; 0.93)		6	0.86 (0.78; 0.96)	
<39	5	0.88 (0.80; 0.97)		5	0.70 (0.55; 0.91)	

**FIGURE 6:**
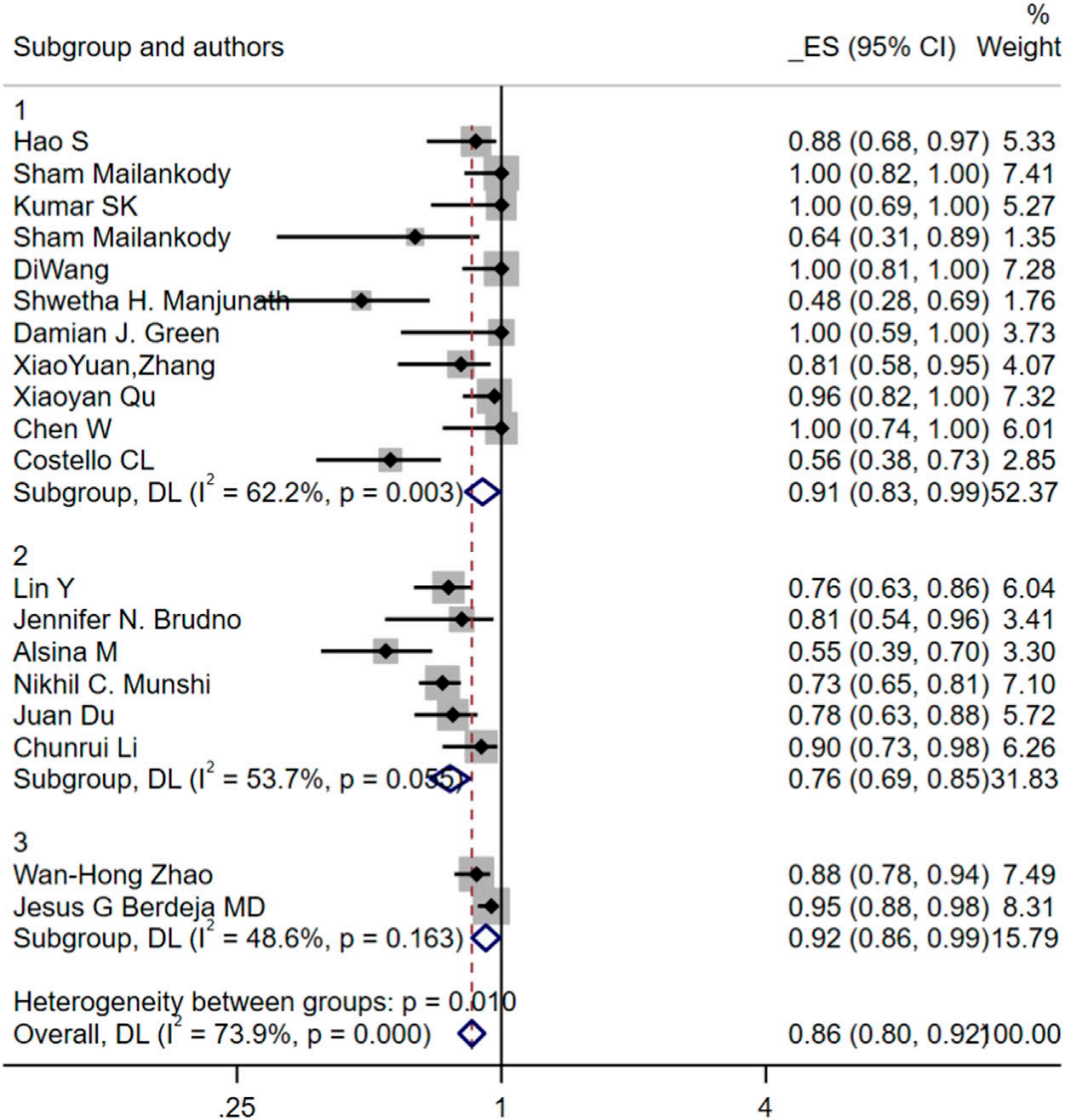
Subgroup analyses of objective response rate by antigen-recognition domain origin (Human vs. Murine vs. Llama) among RRMM.

Subgroup analysis conducted with ORR showed that the ORR in younger patients was higher than in older patients (96% vs. 84%, *p* = 0.016). The ORR subgroup analysis also revealed that patients with better disease status had a considerably greater ORR than others. A substantially greater ORR was attained with patients who received ECOG scores <3 level or ISS score <3 level compared to patients who received worse disease status (94% vs. 78%, *p* = 0.001; 94% vs. 84%, *p* = 0.046). Compared to the patients who receive prior ASCT≥75%, a significantly higher ORR was obtained with patients who receive prior ASCT<75% (90% vs. 78%, *p* = 0.068). Regarding lines of prior treatment, the ORR obtained by lines ≥8 was higher than lines <8 (98% vs. 85%, *p* = 0.011). Subgroup analysis of Antigen-recognition domain origin (Human, Murine, Llama) suggested that there were notable variations in ORR across these three groups. The highest ORR was found in the Llama group, followed by the Human group and Murine group (92% vs. 91% vs. 76%, *p* = 0.010; Figure 6). Subgroup analyses were also performed based on CAR-T cell-infused dose. The enrolled patients were separated into the high dose group (≥200 × 10^6cells or 5 × 10^6 cells/kg) and the low dose group (<200 × 10^6cells or 5 × 10^6 cells/kg) to probe into the correlation between CAR-T infused dose and ORR. Compared to the high-dosage group, the low-dosage group achieved a better ORR (82% vs. 92%, *p* = 0.045). Since seventeen of the twenty-one included studies added 4-1BB costimulatory molecules, ORR subgroup analysis of the costimulatory domain confirmed that CAR-T therapy with 4-1BB in the CAR construct obtained higher ORR than other costimulatory molecules (88% vs. 84%, *p* = 0.351).

However, subgroup analysis of other factors performed with ORR suggested no significant differences. Additional details are shown in Table 4.

**FIGURE 7:**
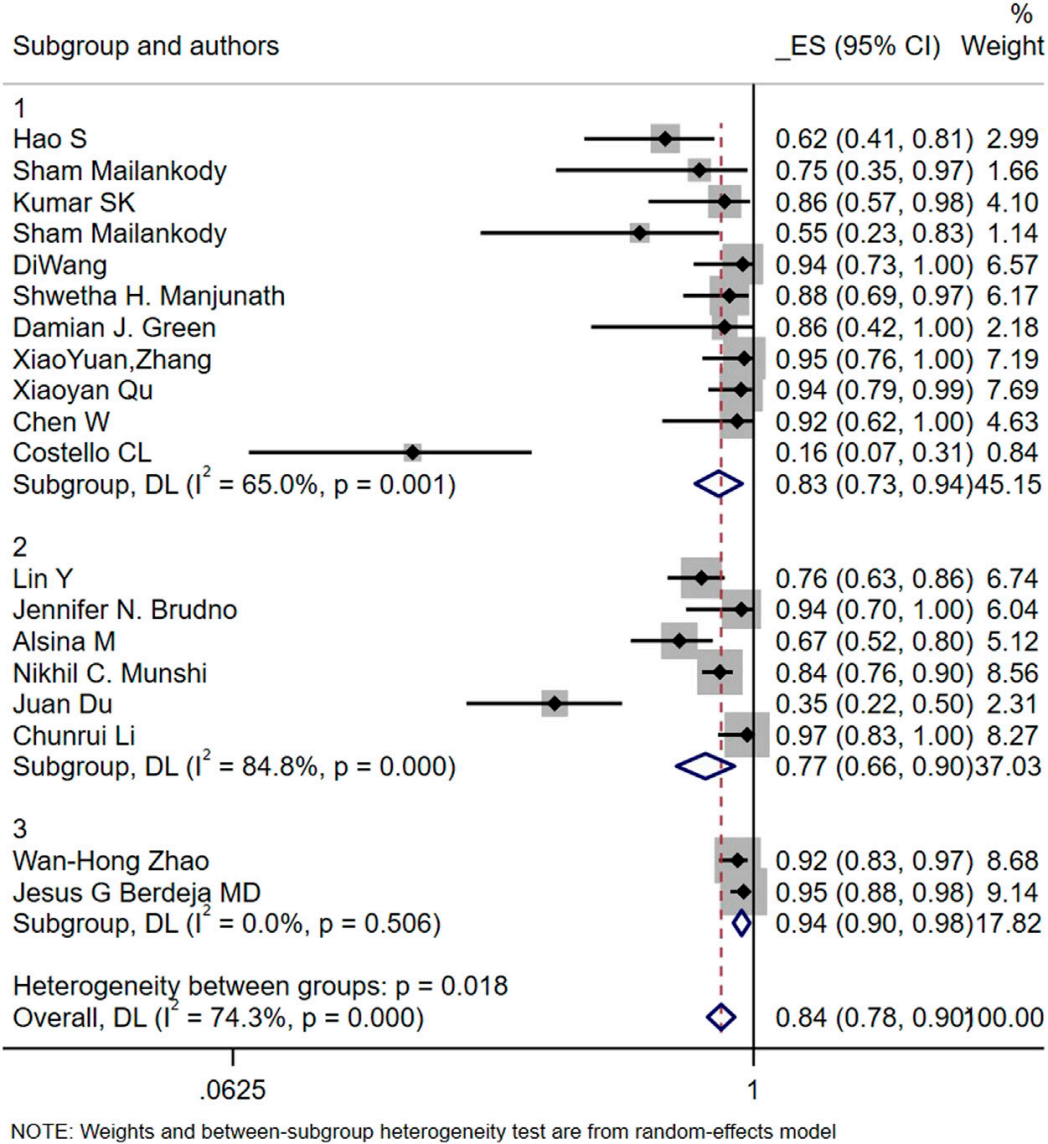
Subgroup analyses of cytokine release syndrome (CRS) by Antigen-recognition domain origin (Human vs. Murine vs. Llama).

Subgroup analysis conducted with any-grade CRS in terms of Antigen-recognition domain origin suggested that the highest risk of CRS was found in the Llama group, followed by the Human group and Murine group (94% vs. 83% vs. 77%, interaction *p* = 0.018; Figure 7). However, the difference in the other subgroup analyses of CRS was not statistically significant.

## 4 Discussion

Modern cancer treatment has already made the switch from traditional chemotherapy to certain immune-based therapeutic approaches. CAR-T treatment, which has undergone substantial development to promote personalized clinical cancer immunotherapy, has shown to be an effective state-of-the-art therapy. This meta-analysis showed that anti-BCMA CAR-T treatment delivered excellent benefits with a manageable safety profile in RRMM patients, looking at 21 prospective trials comprising 761 participants. To improve the effectiveness and safety of a new generation of CAR-T treatment, the findings of this research can serve as a guide for design and optimization. We divided the focus of anti-BCMA CAR-T treatment in relapsed or refractory multiple myeloma into the following parts.

### 4.1 Pre-treatment before CAR-T therapy

Nineteen of twenty-one included studies used cyclophosphamide/fludarabine (Cy-Flu) combination therapy as a lymphodepletion regimen before CAR-T treatment. Subgroup analyses were not performed in this paper due to different doses. However, up to now, opinions on the effect of conditioning scheme on CAR-T treatment varies a lot according to different studies. Xiang et al. (2020) suggested that the effectiveness of CAR-T treatment appeared to be independent of the conditioning scheme, as the combination of Cy-Flu showed similar cell dynamics to that of cyclophosphamide alone; while Di et al. speculated that higher doses of cyclophosphamide might offer potential benefits on response rate and cell persistence by reducing tumor load and enhancing lymphocytosis before CAR-T infusion. But infectious complications and long-term cytopenia may be related to high doses of cyclophosphamide (Wang et al., 2021). New clinical studies are supposed to be designed to directly contrast various cyclophosphamide lymphodepletion regimen dosages to evaluate these two theories.

### 4.2 Applicable population for CAR-T therapy

The results of our meta-analysis provide a reference for relapsed or refractory multiple myeloma patient selection for anti-BCMA CAR-T treatment. Subgroup analysis of ORR by characteristics of the included patients showed that patients with a younger age and a better disease status tended to obtain better efficacy. Notably, compared to the proportion of prior autologous stem cell transplants (ASCT)≥75%, a higher ORR was observed with a higher proportion of prior ASCT<75%, which could be explained as apheresis products of fewer pretreated MM patients containing more available and stronger T cells, leading to better clinical outcomes (Dancy et al., 2018).

### 4.3 Enhance the effectiveness of CAR-T therapy

CAR typically comprises an intracellular domain with co-stimulation and signaling components, a transmembrane domain, and an extracellular antigen-recognition domain (Hao et al., 2020). We performed a subgroup study of ORR based on the antigen-recognition domain origin of the CAR, and the findings revealed that the Llama group had the best effectiveness. However, the prevalence of CRS in the Llama group was noticeably greater, which constrained its potential for use. Patients who received CAR-T cells armored with humanized ScFv had the lowest incidence of CRS and reasonably high rates of remission. The effectiveness and safety profiles of the CAR-T cells generated from murine were comparatively subpar. As a result of the ORR and CRS subgroup analysis of Antigen-recognition domain origin (Human, Murine, Llama), our thorough investigations revealed that humanized CAR-T cells were superior to those produced from Llamas and murine.

Our findings demonstrate that there are inherent limitations in the use of murine scFv-based CARs. The host *versus* graft (HvG) reaction can be brought on by immunogenic epitopes that are present in non-native scFvs (Huang et al., 2020). The therapeutic index of CAR-T cells was constrained and the repeated dosage was resisted due to immunogenicity against CAR-T cells. The mentioned limitations of murine scFvs can be overcome through antibody humanization (Khan et al., 2022). The first method is to replace murine scFv with a fully human binding domain. To avoid the risks associated with possessing protein sequences of non-human origin, Lam et al. have implemented a complete switch from scFvs originating from murine to scFvs containing fully human binding sequences. They created an anti-BCMA CAR (FHVH33-CD8BBξ) with a fully human heavy-chain variable domain (FHVH) (Mikkilineni et al., 2021). In this study, FHVH33-CD8BBξ showed considerable promise, with reduced immunogenicity and toxicity, greater persistence, and a better clinical outcome when compared to murine anti-BCMA CAR (11D5-3) (Xiao-Yuan et al., 2022). The other method is to humanize Murine scFv (Wagner et al., 2021). Murine CDR sequences are grafted onto the human framework region, thus reducing the foreignness in CAR design without loss of its binding properties. Zhao Y et al. (2022) demonstrated the superiority of humanized selective CAR in recurrent/refractory acute B-lymphocytic leukemia patients. Humanized selective CD19-specific CAR-T cells were then used to treat patients who had relapsed after receiving murine-based CAR-T cell treatments. The repeated dose of murine-based CAR-T treatments proved to be ineffective. In contrast, subsequent humanized selective CAR-T treatments were effective in all patients, achieving complete remission. Conclusions from this trial show that humanized selective CAR-T cells had a lower immunogenicity risk, greater therapeutic effectiveness, and enhanced persistence.

Currently, the majority of CAR-T cells use scFvs as their targeting domains. These scFvs have some disadvantages, including the anti-idiotypic responses against the CAR targeting domain (due to the linker peptide or the murine origin of the scFv), and scFv aggregation (tonic signaling) resulting in antigen-independent CAR-T exhaustion (Safarzadeh Kozani et al., 2022). Nanobodies may therefore be a feasible alternative to scFvs for CAR-T cell antigen recognition domains. On the one hand, nanobodies might not be able to aggregate on the surface of T cells because of their monomeric structure. They could consequently help prevent premature T cell activation and exhaustion (Han et al., 2021). On the other hand, the risk of immunogenicity produced by VHH is lower since nanobodies lack linker peptides in scFv. It is worth mentioning that, all of the FDA-approved CAR-T products were CAR-Ts with scFv-based targeting domains. The first VHH-based CAR-T product, ciltacabtagene autoleucel, had encouraging clinical results (Nasiri et al., 2023). Designing and optimizing costimulatory domains in CAR is an essential step to improve the performance of T cells in response to antigens (Mikkilineni and Kochenderfer, 2017). Typically, the costimulatory molecules originate from either the CD28 receptor family (CD28, ICOS) or the tumor necrosis factor receptor family (4-1BB, OX40, CD27) (Sadelain et al., 2013; van der Stegen et al., 2015; Stoiber et al., 2019). Our subgroup analysis of ORR by costimulatory domains confirmed that the 4-1BB domain produced higher cytokine productivity and anti-tumor activity than other costimulatory molecules.

### 4.4 Improve the durability of CAR-T therapy

One of the independent prognostic factors for MM patients is MRD negativity. However, the high combined MRD-negative rate of 78% in responders in this study fails to explain the high relapse rate of 16% within 1 year. Our research confirms the limited prognostic value of a single-time point MRD evaluation. In the design of subsequent clinical trials, the dynamic change of MRD status during maintenance should be used as an endpoint to evaluate the prognostic effect (Paiva, et al., 2023). Meanwhile, previous studies showed that the PFS, OS, and DOR time of MRD-positive patients within responders who responded to CAR-T therapy was significantly shorter than that of MRD-negative patients within responders (Kumar et al., 2016; Munshi et al., 2017; Paiva et al., 2017; Li et al., 2021). Therefore, longer follow-ups for MRD dynamics over time will be required to address whether CAR-T cells have the potential to induce long-lasting remission in RRMM (Du et al., 2022).

In terms of disease recurrence, one of the primary reasons is the limited effectiveness duration of CAR-T treatment (Marple et al., 2020). To assess the durability of CAR-T treatment, we pooled the expansion of available CAR-BCMA T cell data in the twenty-one included studies, and the results are shown in Table 5. The median time for CAR-BCMA T cells to show initiation expansion was 3.5 days (2–5 days), with peak expansion at 11.1 days (10–18 days) after infusion. And the median time for CAR-BCMA T cells to show persistence was 184 days (172–307.5 days), with the longest CAR-BCMA copies persistence at 341 days (308–550 days). To prolong CAR-T-cell persistence, studies demonstrated the feasibility of two approaches (Guo et al., 2022). Currently, two brand-new anti-BCMA CAR-T cell items, namely, P-BCMA-101 (autologous) and P-BCMA-ALLO1 (allogeneic) manufactured by a non-lentivirus transposon system called PiggyBac (PB) were reported (C. Costello et al., 2021). Research has shown that PB can keep more desired stem cell memory T cells (Tscm), whose percentage was significantly associated with both the manufacturing efficiency and the durability of CAR-T cells (Cohen et al., 2019; McLellan and Ali Hosseini Rad, 2019). Drug combinations have been established to solve the poor persistence of CAR-T treatment in addition to drastically increasing their structural quality (Shah and Fry, 2019). For instance, a combined application of CAR-T cell and NKTR-255, a recombinant human IL-15 receptor agonist, potentially increase the growth of Tscm subsets and memory CD8 T cells in tumor-specific T-cell colonies. (Cohen et al., 2019; McLellan and Ali Hosseini Rad, 2019).

**TABLE 5:** The additional measures of anti-BCMA CAR-T therapy.

No	Study	mPFS (months)	mOS (months)	mDOR (months)	CAR-BCMA T cell expansion time (days)	CAR-BCMA T cell reached peak value time (days)	CAR-BCMA T cell highest concentration (copies/µg genomic DNA)	Median CAR-BCMA T cell persistence time (days)	Longest CAR-BCMA copies persistence time (days)	Median CRS occurred time (days)	Median CRS resolved time (days)	Increase of endogenous marker
1	Lin Y	8.8	34.2	10.3	—	—	—	—	—	—	—	—
2	Jennifer N. Brudno	7.8	—	—	—	10	—	—	—	—	—	IFN-γ, IL-6, IL-10, GM-CSF, IL-15, IL-8, IL-4, TNF-α
3	Hao S	18.8	—	21.8	4	14	450000	172	341	2.5	6	—
4	Alsina M	—	—	11.9	—	—	—	—	—	3	—	—
5	Wan-Hong Zhao	18	36.1	23.3	2	18	450000	172	341	9	9	IL-6, IL-10, TNF-α, IL-2, IL-8
6	Sham Mailankody	—	—	—	—	—	—	—	—	9	4.5	—
7	Kumar SK	—	—	21.8	3	10	—	—	—	2	4	CRP, IL-6, IFN-γ, IL-8, IL-10
8	Sham Mailankody	—	—	3.5	—	—	90208	—	—	—	—	CRP, IFN-γ, IL-6
9	DiWang	13	—	10.8	—	12	—	307.5	308	2	8	Ferritin, IL-6
10	Shwetha H. Manjunath	—	9.3	—	—	11	27737	—	—	—	—	Ferritin, CRP
11	Damian J. Green	—	—	—	—	—	—	—	—	—	—	—
12	Jesus G Berdeja MD	—	—	—	—	12.7	—	—	—	7	4	—
13	Nikhil C. Munshi	8.8	19.4	10.7	—	11	231278	119	—	1	5	Ferritin, CRP, IL-6, IFN-γ, IL-8, IL-10
14	XiaoYuan, Zhang	7.9	19.4	—	—	14	261000	—	—	2	5	IL-2, IL-6, IL-10, IFN-γ, LDH, CRP, Ferritin, TNF-α
15	Juan Du	10	29	—	—	11	220453	196	—	3	8	IL-6, IFN-γ
16	Xiaoyan Qu	—	—	—	—	14	750061	—	—	7	5	IL-6, IFN-γ
17	Chen W	—	—	—	—	10	45469	—	—	6	7	CRP, IL-6, IFN-γ, IL-8, IL-10
18	Costello CL	—	—	—	5	18	—	—	550	—	—	IL-6
19	Frigault MJ	—	—	—	—	—	—	—	—	—	—	—
20	Hu Y	—	—	—	3	—	—	—	—	—	—	—
21	Chunrui Li	5.3	14.2	4.9	—	—	—	—	—	—	—	Ferritin, IL-6
	Overall	8.77	18.87	10.32	3.50	11.10	225865.50	184.0	341.0	4.80	4.50	CRP, Ferritin, IFN-γ, IL-6, IL-10, GM-CSF, IL-15, IL-2, IL-8, IL-4, TNF-α, LDH

### 4.5 Increase the safety of CAR-T therapy

Despite the excellent efficacy, toxicities after treatment limited the widespread utilization of anti-BCMA CAR-T treatment in RRMM (Miguel et al., 2013; Lonial et al., 2016; Chen et al., 2018). CAR-T toxicity can result from a multitude of causes, including CAR design, infused dosages, patient disease load, and so on (Brudno and Kochenderfer, 2019). The most common adverse effect after CAR-T infusion is a systemic inflammatory reaction known as CRS, which causes numerous additional disorders such as tachycardia, hypotension, and fever (L. Mikkilineni and Kochenderfer, 2017). The number of cytokines in serum has a direct impact on how severe CRS is. Except for summarizing the kinds of cytokine levels that were elevated in Table 5, we also counted the early appearance and peak time of cytokines. We found that thirteen of the twenty-one included articles reported plasma cytokines positively associated with CRS grade and summarized as follows: CRP, Ferritin, IFN-γ, IL-6, IL-10, GM-CSF, IL-15, IL-2, IL-8, IL-4, TNF-α, and LDH. The median CRS occurred time was 4.80 days (95% CI: 3.92–5.67) after infusion, with a median resolved time of 4.50 days (95% CI: 3.42–5.59). The findings of our study serve as a guide for the time of intervention for CRS toxicity after CAR-T cell infusion and will avoid serious consequences. Supportive care and immunosuppression with tocilizumab and corticosteroids are frequently used in the management of toxicity (Lee et al., 2014; Bonifantet al., 2016; Brudno and Kochenderfer, 2016). The result of 21 articles included suggested that the pooled usage rate of tocilizumab was 46% (95% CI: 33%–59%). However, it is crucial to emphasize that using steroids for longer than 5 days may have a negative impact on the PFS of CAR-T treatment to some extent (Duvalyan E, Lo M, and T., 2021). Consequently, further mechanistic understanding and new treatment strategies for these toxicities are needed to improve the efficacy-to-toxicity ratio of CAR-T treatment.

In terms of Antigen-recognition domain origin, the results of the subgroup analysis with CRS revealed that the human group has a higher risk of CRS than the murine group. The reason may be the direct relationship between the severity of toxicity and the persistence of infused cells. When compared to their murine counterparts, humanized CAR-T cells with higher persistence have a higher risk of toxicity (Safarzadeh Kozani et al., 2021). In addition, CAR-T cells have the potential to trigger humoral and cellular anti-CAR immune responses. Pre-existing antibodies that broadly recognize the scFvs of mouse immunoglobulins are called human anti-mouse antibodies (HAMAs). Antibodies directed towards human or humanized scFvs are known as anti-idiotype antibodies. There is yet no solid proof that such anti-CAR immune responses contribute to adverse events like CRS and CRES. Therefore, head-to-head clinical trials directly comparing the toxicity of mouse-derived and humanized scFvs are warranted.

### 4.6 The development trend of CAR-T treatment

In summary, our research indicates that the anti-BCMA CAR-T product has achieved breakthrough effectiveness in the treatment of multiple myeloma, but there are still limitations for improvement. In the future, researchers have the potential to design a series of advanced CAR-T treatment strategies to provide patients in more disease areas with higher effectiveness and safety. Firstly, to solve the problem of antigen escape during disease recurrence, bispecific, dual-target, and multi-target CAR-T were designed and developed. The results of previous studies have shown that LCAR-B38M and combined CD19/BCMA exhibit higher overall response rates and deeper responses compared to specific BCMA (Xiang et al., 2020). Secondly, it was discovered during the manufacturing of CAR-T cells that most of the antigen recognition domain of CAR was a single-chain variable fragment, which was difficult to effectively fold. This led to the development of nano-antibody CAR-T, which has a more straightforward structure, reduced immunogenicity, and greater stability (Li et al., 2022). Thirdly, due to prolonged drug induction therapy, individuals with multiple myeloma frequently have insufficient numbers and quality of T cells. As a result, the fifth-generation universal CAR-T can obtain unlimited allogeneic healthy cells in advance for mass production, which greatly reduces the time and economic cost of the preparation process. Gene editing techniques including CRISPR-Cas9, TALEN, ZFN, and others were employed to solve the allograft rejection caused by universal CAR-T (Dimitri et al., 2022). Finally, the most intriguing discovery shows that CAR-T therapy can potentially be utilized to treat solid tumors when combined with oncolytic viruses. Take the CD19 antigen as an example. The oncolytic virus is first genetically modified to express CD19 protein, which is then utilized to infect and tag tumor cells. Finally, the CD19-targeted CAR-T cells are employed to kill the labeled tumor cells. Precision medicine can be utilized to treat solid tumors owing to the combination of CAR-T therapy and oncolytic viruses (Park et al., 2020). With the continuous update and iteration of cell therapy and its combination with gene editing technology, the treatment of cancer, tumors, organ failure, and other fields will make significant strides in the coming decades.

### 4.7 Strengths and weaknesses

We acknowledge certain limitations of our study. Firstly, the quality of the included studies was assessed as having considerable risk of bias and statistical heterogeneity. On the one hand, all were early-phase studies without a control group that likely experienced selection bias. On the other hand, there may also be a risk of confounding biases due to variations in the baseline characteristics, performance status, or disease condition after different prior treatments. Secondly, with a limited sample size of the included studies, the estimate of subgroup analysis may underestimate or overstate the pooled proportions. Due to a paucity of information, we also did not assess the data on specific subgroups, such as prior therapies, BCMA expression, and CAR-T persistence. Thirdly, cytokine release syndrome and neurological toxicity were the most common toxicities of CAR-T therapy. It is challenging to compare the safety of different products because the evaluation and grading of these toxicities vary greatly between clinical trials and institutions.

Despite the limitations of our study, the following strengths of this systematic review and meta-analysis should be noted: First of all, the study thoroughly detailed the structure of the CAR-T products (including the costimulatory domains, extracellular antigen-recognition domains, and distinctive features). To visually illustrate the impact of CAR-T composition on outcome indicators, we first did a subgroup analysis in RRMM with CAR-T therapy. Additionally, we systematically evaluated eighteen efficacy and safety outcome measures. In contrast to previous studies, our research focuses on the duration of BCMA-CART cell expansion and the onset of toxic side effects for the first time, which facilitates prolonging CAR-T effects and developing optimal strategies for the management of toxicities. Furthermore, a random-effects model was applied and eleven subgroup analyses were conducted to reduce heterogeneity. Our research first offered the subgroup analysis of both efficacy and safety based on antigen-recognition domain origin, which provides sufficient evidence for designing a fully humanized construct for the following-generation CAR-T. Also, to harmonize the definitions and grading systems for CRS and neurotoxicity, we refer to ASTCT Consensus Grading for Cytokine Release Syndrome and Neurologic Toxicity Associated with Immune Effector Cell (Lee DW et al., 2019). This consensus proposes new definitions and grading for CRS and neurotoxicity that are objective, easy to apply, and ultimately more accurately categorize the severity of these toxicities. Our goal is to provide a uniform consensus grading system for CRS and neurotoxicity associated with immune effector cell therapies, for use across clinical trials and in the post-approval clinical setting. Finally, we offer appropriate improvement measures in response to the limitations of this research and the deficiencies of anti-BCMA CAR-T therapy in RRMM. These approaches will be of great benefit to the future product design, clinical trials, and clinical application of anti-BCMA CAR-T.

## 5 Conclusion

In conclusion, this meta-analysis offers compelling proof of the favorable effectiveness and safety of anti-BCMA CAR-T treatment in RRMM patients and reveals a number of patient-related and treatment-related influence factors. Our research could help with the development of CAR-T treatment regimens for the next-generation and the optimization of clinical applications.

## Data Availability

The original contributions presented in the study are included in the article/Supplementary Material, further inquiries can be directed to the corresponding authors.

## References

[B1] Alsina M. Shah N. Raje N. S. Jagannath S. Madduri D. Kaufman J. L. (2020). Updated results from the phase I CRB-402 study of anti-bcma CAR-T cell therapy bb21217 in patients with relapsed and refractory multiple myeloma: Correlation of expansion and duration of response with T cell phenotypes. Blood 136 (1), 25–26. 10.1182/blood-2020-140410

[B2] Berdeja J. G. Madduri D. Usmani S. Z. Jakubowiak A. Agha M. Cohen A. D. (2021). Ciltacabtagene autoleucel, a B-cell maturation antigen-directed chimeric antigen receptor T-cell therapy in patients with relapsed or refractory multiple myeloma (CARTITUDE-1): A phase 1b/2 open-label study. Lancet 398 (10297), 314–324. 10.1016/s0140-6736(21)00933-8 34175021

[B3] Bonifant C. L. Jackson H. J. Brentjens R. J. Curran K. J. (2016). Toxicity and management in CAR T-cell therapy. Mol. Ther. Oncolytics 3, 16011. 10.1038/mto.2016.11 27626062 PMC5008265

[B4] Brudno J. N. Kochenderfer J. N. (2019). Recent advances in CAR T-cell toxicity: Mechanisms, manifestations and management. Blood Rev. 34, 45–55. 10.1016/j.blre.2018.11.002 30528964 PMC6628697

[B5] Brudno J. N. Kochenderfer J. N. (2016). Toxicities of chimeric antigen receptor T cells: Recognition and management. Blood 127 (26), 3321–3330. 10.1182/blood-2016-04-703751 27207799 PMC4929924

[B6] Brudno J. N. Maric I. Hartman S. D. Rose J. J. Wang M. Lam N. (2018). T cells genetically modified to express an anti-B-cell maturation antigen chimeric antigen receptor cause remissions of poor-prognosis relapsed multiple myeloma. J. Clin. Oncol. 36 (22), 2267–2280. 10.1200/JCO.2018.77.8084 29812997 PMC6067798

[B7] Chen C. Siegel D. Gutierrez M. Jacoby M. Hofmeister C. C. Gabrail N. (2018). Safety and efficacy of selinexor in relapsed or refractory multiple myeloma and Waldenstrom macroglobulinemia. Blood 131 (8), 855–863. 10.1182/blood-2017-08-797886 29203585

[B8] Chen H. Li M. Sanchez E. Soof C. Patil S. , (2017). Serum bcma may interfere with anti-bcma-CAR-transduced T cells or other anti-bcma antibody-based immunotherapy in multiple myeloma. Blood 130, 4413. 10.1182/blood.V130.Suppl_1.4413.4413

[B9] Chen W. Fu C. Cai Z. Li Z. Wang H. Yan L. (2020). Results from lummicar-1: A phase 1 study of fully human B-cell maturation antigen-specific CAR T cells (CT053) in Chinese subjects with relapsed and/or refractory multiple myeloma. Blood 136 (1), 49–50. 10.1182/blood-2020-140727

[B10] Cohen A. D. Garfall A. L. Stadtmauer E. A. Melenhorst J. J. Lacey S. F. Lancaster E. (2019). B cell maturation antigen-specific CAR T cells are clinically active in multiple myeloma. J. Clin. Invest. 129 (6), 2210–2221. 10.1172/JCI126397 30896447 PMC6546468

[B11] Costello C. Derman B. A. Kocoglu M. H. Deol A. Ali A. A. Gregory T. (2021). Clinical trials of BCMA-targeted CAR-T cells utilizing a novel non-viral transposon system. Blood 138 (1), 3858. 10.1182/blood-2021-151672

[B12] Costello C. L. Cohen A. D. Patel K. K. Ali S. S. Berdeja J. G. Shah N. (2020). Phase 1/2 study of the safety and response of P-BCMA-101 CAR-T cells in patients with relapsed/refractory (r/r) multiple myeloma (MM) (PRIME) with novel therapeutic strategies. Blood 136 (1), 29–30. 10.1182/blood-2020-142695

[B13] Dancy E. Garfall A. L. Cohen A. D. Fraietta J. A. Davis M. Levine B. L. (2018). Clini-cal predictors of T cell fitness for CAR T cell manufacturing and efficacy in multiple myeloma. Blood 132, 1886. 10.1182/blood-2018-99-115319

[B14] Davila M. L. Riviere I. Wang X. Bartido S. Park J. Curran K. (2014). Efficacy and toxicity management of 19-28z CAR T cell therapy in B cell acute lymphoblastic leukemia. Sci. Transl. Med. 6 (224), 224ra25. 10.1126/scitranslmed.3008226 PMC4684949 24553386

[B15] Dimitri A. Herbst F. Fraietta J. A. (2022). Engineering the next-generation of CAR T-cells with CRISPR-Cas9 gene editing. Mol. Cancer 21 (1), 78. 10.1186/s12943-022-01559-z 35303871 PMC8932053

[B16] Drent E. Groen R. W. Noort W. A. Themeli M. Lammerts van Bueren J. J. Parren P. W. H. I. (2016). 'Pre-clinical evaluation of CD38 chimeric antigen receptor engineered T cells for the treatment of multiple myeloma. Haematologica 101, 616–625. 10.3324/haematol.2015.137620 26858358 PMC5004365

[B17] Du J. Wei R. Jiang S. Jiang H. Li L. Qiang W. (2022). CAR-T cell therapy targeting B cell maturation antigen is effective for relapsed/refractory multiple myeloma, including cases with poor performance status. Am. J. Hematol. 97 (7), 933–941. 10.1002/ajh.26583 35488407

[B18] Duvalyan E. Lo M. Wolf J. L. Chung A. Arora S. , (2021). Impact of corticosteroids on efficacy of BCMA targeted CAR-T therapy in multiple myeloma. Blood 138, 1759. 10.1182/blood-2021-146678 37599633

[B19] Frigault M. J. Bishop M. R. (2020). Phase 1 study of CART-Ddbcma, a CAR-T therapy utilizing a novel synthetic binding domain for the treatment of subjects with relapsed and refractory multiple myeloma. Blood 2020, 136. 10.1200/jco.2021.39.15_suppl.8015 PMC9989524 35468618

[B20] Gagelmann N. Ayuk F. Atanackovic D. Kroger N. (2020). B cell maturation antigen-specific chimeric antigen receptor T cells for relapsed or refractory multiple myeloma: A meta-analysis. Eur. J. Haematol. 104 (4), 318–327. 10.1111/ejh.13380 31883150

[B21] Golubovskaya V. Zhou H. Li F. Berahovich R. Sun J. Valentine M. (2021). 'Novel CS1 CAR-T cells and bispecific CS1-BCMA CAR-T cells effectively target multiple myeloma. Biomedicines 9, 1422. 10.3390/biomedicines9101422 34680541 PMC8533376

[B22] Green D. J. Pont M. Sather B. D. Cowan A. J. Turtle C. J. Till B. G. (2018). Fully human bcma targeted chimeric antigen receptor T cells administered in a defined composition demonstrate potency at low doses in advanced stage high risk multiple myeloma. Blood 132 (1), 1011. 10.1182/blood-2018-99-117729

[B23] Guo R. Lu W. Zhang Y. Cao X. Jin X. Zhao M. (2022). Targeting BCMA to treat multiple myeloma: Updates from the 2021 ASH annual meeting. Front. Immunol. 13, 839097. 10.3389/fimmu.2022.839097 35320942 PMC8936073

[B24] Han L. Zhang J. S. Zhou J. Zhou K. S. Xu B. L. Li L. L. (2021). Single VHH-directed BCMA CAR-T cells cause remission of relapsed/refractory multiple myeloma. Leukemia 35 (10), 3002–3006. Epub 2021 May 24. PMID: 34031533; PMCID: PMC8478646. 10.1038/s41375-021-01269-3 34031533 PMC8478646

[B25] Hao S. Jin J. Jiang S. Li Z. Zhang W. Yang M. (2020). Two-year follow-up of investigator-initiated phase 1 trials of the safety and efficacy of fully human anti-bcma CAR T cells (CT053) in relapsed/refractory multiple myeloma. Blood 136 (1), 27–28. 10.1182/blood-2020-140156

[B26] Hu Y. Yanlei Z. Wei G. alex Hong C. Huang H. (2019). Potent anti-tumor activity of bcma CAR-T therapy against heavily treated multiple myeloma and dynamics of immune cell subsets using single-cell mass cytometry. Blood 134 (1), 1859. 10.1182/blood-2019-130341 31481482

[B27] Huang R. Li X. He Y. Zhu W. Gao L. Liu Y. (2020). Recent advances in CAR-T cell engineering. J. Hematol. Oncol. 13 (1), 86. 10.1186/s13045-020-00910-5 32616000 PMC7333410

[B28] Khan A. N. Chowdhury A. Karulkar A. Jaiswal A. K. Banik A. Asija S. (2022). Immunogenicity of CAR-T cell therapeutics: Evidence, mechanism and mitigation. Front. Immunol. 13, 886546. PMID: 35677038; PMCID: PMC9169153. 10.3389/fimmu.2022.886546 35677038 PMC9169153

[B29] Knobloch K. Yoon U. Vogt P. M. (2011). Preferred reporting items for systematic reviews and meta-analyses (PRISMA) statement and publication bias. J. Craniomaxillofac Surg. 39 (2), 91–92. 10.1016/j.jcms.2010.11.001 21145753

[B30] Kumar S. K. Baz R. C. Orlowski R. Z. Anderson L. D. Ma H. Shrewsbury A. (2020). Results from lummicar-2: A phase 1b/2 study of fully human B-cell maturation antigen-specific CAR T cells (CT053) in patients with relapsed and/or refractory multiple myeloma. Blood 136 (1), 28–29. 10.1182/blood-2020-139802

[B31] Kumar S. Paiva B. Anderson K. C. Durie B. Landgren O. Moreau P. (2016). International Myeloma Working Group consensus criteria for response and minimal residual disease assessment in multiple myeloma. Lancet Oncol. 17 (8), e328–e346. 10.1016/S1470-2045(16)30206-6 27511158

[B32] Lam N. Trinklein N. D. Buelow B. Patterson G. H. Ojha N. Kochenderfer J. N. (2020). Anti-BCMA chimeric antigen receptors with fully human heavy-chain-only antigen recognition domains. Nat. Commun. 11, 283. 10.1038/s41467-019-14119-9 31941907 PMC6962219

[B33] Laurent S. A. Hoffmann F. S. Kuhn P. H. Cheng Q. Chu Y. Schmidt-Supprian M. (2015). γ-Secretase directly sheds the survival receptor BCMA from plasma cells. Nat. Commun. 6, 7333. 10.1038/ncomms8333 26065893 PMC4490565

[B34] Lee D. W. Santomasso B. D. Locke F. L. Ghobadi A. Turtle C. J. Brudno J. N. (2019). ASTCT consensus grading for cytokine release syndrome and neurologic toxicity associated with immune effector cells. Biol. Blood Marrow Transpl. 25 (4), 625–638. Epub 2018 Dec 25. PMID: 30592986. 10.1016/j.bbmt.2018.12.758 PMC12180426 30592986

[B35] Lee D. W. Gardner R. Porter D. L. Louis C. U. Ahmed N. Jensen M. (2014). Current concepts in the diagnosis and management of cytokine release syndrome. Blood 124 (2), 188–195. 10.1182/blood-2014-05-552729 24876563 PMC4093680

[B36] Li C. Cao W. Que Y. Wang Q. Xiao Y. Gu C. (2021). A phase I study of anti-BCMA CAR T cell therapy in relapsed/refractory multiple myeloma and plasma cell leukemia. Clin. Transl. Med. 11 (3), e346. 10.1002/ctm2.346 33784005 PMC7943908

[B37] Li H. Zhong D. Luo H. Shi W. Xie S. Qiang H. (2022). Nanobody-based CAR T cells targeting intracellular tumor antigens. Biomed. Pharmacother. 156, 113919. 10.1016/j.biopha.2022.113919 36411612

[B38] Lin Y. Raje N. S. Berdeja J. G. Siegel D. S. Jagannath S. Madduri D. (2020). Idecabtagene vicleucel (ide-cel, bb2121), a BCMA-directed CAR T cell therapy, in patients with relapsed and refractory multiple myeloma: Updated results from phase 1 CRB-401 study. Blood 136 (1), 26–27. 10.1167/tvst.9.13.26

[B39] Lipe B. Vukas R. Mikhael J. (2016). The role of maintenance therapy in multiple myeloma. Blood Cancer J. 6 (10), e485. 10.1038/bcj.2016.89 27768093 PMC5098261

[B40] Lonial S. Weiss B. M. Usmani S. Z. Singhal S. Chari A. Bahlis N. J. (2016). Daratumumab monotherapy in patients with treatment-refractory multiple myeloma (SIRIUS): An open-label, randomised, phase 2 trial. Lancet 387 (10027), 1551–1560. 10.1016/S0140-6736(15)01120-4 26778538

[B41] Luanpitpong S. Poohadsuan J. Klaihmon P. Issaragrisil S. (2021). 'Selective cytotoxicity of single and dual anti-CD19 and anti-cd138 chimeric antigen receptor-natural killer cells against hematologic malignancies. J. Immunol. Res. 2021, 5562630. 10.1155/2021/5562630 34337077 PMC8289607

[B42] Madry C. Laabi Y. Callebaut I. Roussel J. Hatzoglou A. Le Coniat M. (1998). The characterization of murine BCMA gene defines it as a new member of the tumor necrosis factor receptor superfamily. Int. Immunol. 10 (11), 1693–1702. 10.1093/intimm/10.11.1693 9846698

[B43] Mailankody S. Ghosh A. Staehr M. Purdon T. J. Roshal M. Halton E. (2018). Clinical responses and pharmacokinetics of MCARH171, a human-derived bcma targeted CAR T cell therapy in relapsed/refractory multiple myeloma: Final results of a phase I clinical trial. Blood 132 (1), 959. 10.1182/blood-2018-99-119717

[B44] Mailankody S. Htut M. Lee K. P. Bensinger W. Devries T. Piasecki J. (2018). JCARH125, anti-BCMA CAR T-cell therapy for relapsed/refractory multiple myeloma: Initial proof of concept results from a phase 1/2 multicenter study (EVOLVE). Blood 132 (1), 957. 10.1182/blood-2018-99-113548

[B45] Manier S. Ingegnere T. Escure G. Prodhomme C. Nudel M. Mitra S. (2022). Current state and next-generation CAR-T cells in multiple myeloma. Blood Rev. 54, 100929. 10.1016/j.blre.2022.100929 35131139

[B46] Manjunath S. H. Cohen A. D. Lacey S. F. Davis M. M. Garfall A. L. Melenhorst J. J. (2021). The safety of bridging radiation with anti-BCMA CAR T-cell therapy for multiple myeloma. Clin. Cancer Res. 27 (23), 6580–6590. 10.1158/1078-0432.CCR-21-0308 34526365 PMC8639780

[B47] Marple A. H. Bonifant C. L. Shah N. N. (2020). Improving CAR T-cells: The next generation. Semin. Hematol. 57 (3), 115–121. 10.1053/j.seminhematol.2020.07.002 33256900

[B48] McLellan A. D. Ali Hosseini Rad S. M. (2019). Chimeric antigen receptor T cell persistence and memory cell formation. Immunol. Cell Biol. 97 (7), 664–674. 10.1111/imcb.12254 31009109

[B49] Miguel J. S. Weisel K. Moreau P. Lacy M. Song K. Delforge M. (2013). Pomalidomide plus low-dose dexamethasone versus high-dose dexamethasone alone for patients with relapsed and refractory multiple myeloma (MM-003): A randomised, open-label, phase 3 trial. Lancet Oncol. 14 (11), 1055–1066. 10.1016/S1470-2045(13)70380-2 24007748

[B50] Mikkilineni L. Kochenderfer J. N. (2017). Chimeric antigen receptor T-cell therapies for multiple myeloma. Blood 130 (24), 2594–2602. 10.1182/blood-2017-06-793869 28928126 PMC5731088

[B51] Mikkilineni L. Manasanch E. E. Natrakul D. Brudno J. N. Mann J. Goff S. L. (2021). Treatment of patients with T cells expressing a fully-human anti-BCMA CAR with a heavy-chain antigen-recognition domain caused high rates of sustained complete responses and relatively mild toxicity. Blood 138 (1), 3837. 10.1182/blood-2021-152688

[B52] Mueller K. P. Piscopo N. J. Forsberg M. H. Saraspe L. A. Das A. Russell B. (2022). 'Production and characterization of virus-free, CRISPR-CAR T cells capable of inducing solid tumor regression. J. Immunother. Cancer 10, e004446. 10.1136/jitc-2021-004446 36382633 PMC9454086

[B53] Munshi N. C. Anderson L. D. Jr. Shah N. Madduri D. Berdeja J. Lonial S. (2021). Idecabtagene vicleucel in relapsed and refractory multiple myeloma. N. Engl. J. Med. 384 (8), 705–716. 10.1056/NEJMoa2024850 33626253

[B54] Munshi N. C. Avet-Loiseau H. Rawstron A. C. Owen R. G. Child J. A. Thakurta A. (2017). Association of minimal residual disease with superior survival outcomes in patients with multiple myeloma: A meta-analysis. JAMA Oncol. 3 (1), 28–35. 10.1001/jamaoncol.2016.3160 27632282 PMC5943640

[B55] Nasiri F. Safarzadeh Kozani P. Rahbarizadeh F. (2023). T-cells engineered with a novel VHH-based chimeric antigen receptor against CD19 exhibit comparable tumoricidal efficacy to their FMC63-based counterparts. Front. Immunol. 14, 1063838. PMID: 36875091; PMCID: PMC9978144. 10.3389/fimmu.2023.1063838 36875091 PMC9978144

[B56] PaivaB. ManriqueI. DimopoulosM. A. GayF. MinC. K. ZweegmanS. (2023). MRD dynamics during maintenance for improved prognostication of 1280 patients with myeloma in the TOURMALINE-MM3 and -MM4 trials. Blood 141 (6), 579–591. PMID: 36130300. 10.1182/blood.2022016782 36130300

[B57] Paiva B. Puig N. Cedena M. T. de Jong B. G. Ruiz Y. Rapado I. (2017). Differentiation stage of myeloma plasma cells: Biological and clinical significance. Leukemia 31 (2), 382–392. 10.1038/leu.2016.211 27479184 PMC5439510

[B58] Park A. K. Fong Y. Kim S. I. Yang J. Murad J. P. Lu J. (2020). Effective combination immunotherapy using oncolytic viruses to deliver CAR targets to solid tumors. Sci. Transl. Med. 12 (559), eaaz1863. 10.1126/scitranslmed.aaz1863 32878978 PMC9126033

[B59] Pont M. J. Hill T. Cole G. O. Abbott J. J. Kelliher J. Salter A. I. (2019). γ-Secretase inhibition increases efficacy of BCMA-specific chimeric antigen receptor T cells in multiple myeloma. Blood 134 (19), 1585–1597. 10.1182/blood.2019000050 31558469 PMC6871311

[B60] Qu X. An G. Sui W. Wang T. Zhang X. Yang J. (2022). Phase 1 study of C-CAR088, a novel humanized anti-BCMA CAR T-cell therapy in relapsed/refractory multiple myeloma. J. Immunother. Cancer 10 (9), e005145. 10.1136/jitc-2022-005145 36100310 PMC9472147

[B61] Roex G. Timmers M. Wouters K. Campillo-Davo D. Flumens D. Schroyens W. (2020). Safety and clinical efficacy of BCMA CAR-T-cell therapy in multiple myeloma. J. Hematol. Oncol. 13 (1), 164. 10.1186/s13045-020-01001-1 33272302 PMC7713173

[B62] Sadelain M. Brentjens R. Riviere I. (2013). The basic principles of chimeric antigen receptor design. Cancer Discov. 3 (4), 388–398. 10.1158/2159-8290.CD-12-0548 23550147 PMC3667586

[B63] Safarzadeh Kozani P. Naseri A. Mirarefin S. M. J. Salem F. Nikbakht M. Evazi Bakhshi S. (2022). Nanobody-based CAR-T cells for cancer immunotherapy. Biomark. Res. 10 (1), 24. PMID: 35468841; PMCID: PMC9036779. 10.1186/s40364-022-00371-7 35468841 PMC9036779

[B64] Safarzadeh Kozani P. Safarzadeh Kozani P. O'Connor R. S. (2021). In like a lamb; out like a lion: Marching CAR T cells toward enhanced efficacy in B-all. Mol. Cancer Ther. 20 (7), 1223–1233. Epub 2021 Apr 26. PMID: 33903140; PMCID: PMC8285067. 10.1158/1535-7163.MCT-20-1089 33903140 PMC8285067

[B65] Schmidts A. Ormhøj M. Choi B. D. Taylor A. O. Bouffard A. A. Scarfò I. (2019). 'Rational design of a trimeric APRIL-based CAR-binding domain enables efficient targeting of multiple myeloma. Blood Adv. 3, 3248–3260. 10.1182/bloodadvances.2019000703 31698455 PMC6855119

[B66] Sevcikova S. Minarik J. Stork M. Jelinek T. Pour L. Hajek R. (2019). Extramedullary disease in multiple myeloma - controversies and future directions. Blood Rev. 36, 32–39. 10.1016/j.blre.2019.04.002 31005420

[B67] Shah N. N. Fry T. J. (2019). Mechanisms of resistance to CAR T cell therapy. Nat. Rev. Clin. Oncol. 16 (6), 372–385. 10.1038/s41571-019-0184-6 30837712 PMC8214555

[B68] Slim K. Nini E. Forestier D. Kwiatkowski F. Panis Y. Chipponi J. (2003). Methodological index for non-randomized studies (minors): Development and validation of a new instrument. ANZ J. Surg. 73 (9), 712–716. 10.1046/j.1445-2197.2003.02748.x 12956787

[B69] Stoiber S. Cadilha B. L. Benmebarek M. R. Lesch S. Endres S. Kobold S. (2019). Limitations in the design of chimeric antigen receptors for cancer therapy. Cells 8 (5), 472. 10.3390/cells8050472 31108883 PMC6562702

[B70] Sun Y. Fang M. Y. Liu Y. J. (2010). Immunophenotype characteristics in multiple myeloma cells and their significance. Zhongguo Shi Yan Xue Ye Xue Za Zhi 18, 381–384. 20416173

[B71] van der Stegen S. J. Hamieh M. Sadelain M. (2015). The pharmacology of second-generation chimeric antigen receptors. Nat. Rev. Drug Discov. 14 (7), 499–509. 10.1038/nrd4597 26129802 PMC6410718

[B72] Wagner D. L. Fritsche E. Pulsipher M. A. Ahmed N. Hamieh M. Hegde M. (2021). Immunogenicity of CAR T cells in cancer therapy. Nat. Rev. Clin. Oncol. 18 (6), 379–393. Epub 2021 Feb 25. PMID: 33633361; PMCID: PMC8923136. 10.1038/s41571-021-00476-2 33633361 PMC8923136

[B73] Wang D. Wang J. Hu G. Wang W. Cai H. Xiao Y. (2021). A phase 1 study of a novel fully human BCMA-targeting CAR (CT103A) in patients with relapsed/refractory multiple myeloma. Blood 137 (21), 2890–2901. 10.1182/blood.2020008936 33512480

[B74] Xiang X. He Q. Ou Y. Wang W. Wu Y. (2020). Efficacy and safety of CAR-modified T cell therapy in patients with relapsed or refractory multiple myeloma: A meta-analysis of prospective clinical trials. Front. Pharmacol. 11, 544754. 10.3389/fphar.2020.544754 33343342 PMC7744881

[B75] Xiao-Yuan Z. Han-Yi D. Dong-Xu G. (2022). Toxicity management and efficacy evaluation of BCMA-CART in the treatment of relapsed and refractory multiple myeloma. J. Exp. Hematol. 30 (2), 466–475. 10.19746/j.cnki.issn.1009-2137.2022.02.024 35395981

[B76] Yang Q. Li X. Zhang F. Yang Q. Zhou W. Liu J. (2021). Efficacy and safety of CAR-T therapy for relapse or refractory multiple myeloma: A systematic review and meta-analysis. Int. J. Med. Sci. 18 (8), 1786–1797. 10.7150/ijms.46811 33746596 PMC7976586

[B77] Zhang L. Shen X. Yu W. Li J. Zhang J. Zhang R. (2021). Comprehensive meta-analysis of anti-BCMA chimeric antigen receptor T-cell therapy in relapsed or refractory multiple myeloma. Ann. Med. 53 (1), 1547–1559. 10.1080/07853890.2021.1970218 34459681 PMC8409966

[B78] Zhao A. Zhao M. Qian W. Liang A. Li P. Liu H. (2022). 'Secondary myeloid neoplasms after CD19 CAR T therapy in patients with refractory/relapsed B-cell lymphoma: Case series and review of literature. Front. Immunol. 13, 1063986. 10.3389/fimmu.2022.1063986 36713414 PMC9880439

[B79] Zhao W. H. Wang B. Y. Chen L. J. Fu W. J. Xu J. Liu J. (2022). Four-year follow-up of LCAR-B38M in relapsed or refractory multiple myeloma: A phase 1, single-arm, open-label, multicenter study in China (LEGEND-2). J. Hematol. Oncol. 15 (1), 86. 10.1186/s13045-022-01301-8 35794616 PMC9261106

[B80] Zhao Y Y. Zhang J. Yang J. Wu H. Chen Y. Li N. (2022). Long-term safety and efficacy of CD19 humanized selective CAR-T therapy in B-all patients who have previously received murine-based CD19 CAR-T therapy. Front. Oncol. 12, 884782. PMID: 35800047; PMCID: PMC9253302. 10.3389/fonc.2022.884782 35800047 PMC9253302

