# Supplementary material for: Effectiveness and safety of anti-BCMA chimeric antigen receptor T-cell treatment in relapsed/refractory multiple myeloma: a comprehensive review and meta-analysis of prospective clinical trials
Source: Front Pharmacol. 2023 Jun 20;14:1149138. doi: 10.3389/fphar.2023.1149138 (PMC10318167; doi:10.3389/fphar.2023.1149138)

Supplemental Table 1: Search strategy

| ID | Search |
| --- | --- |
| #1 | **(((((((chimeric**[Title/Abstract]**) OR (chimera**[Title/Abstract]**)) OR (chimer**[Title/Abstract]**)) OR (chimerical**[Title/Abstract]**)) OR (chimerics**[Title/Abstract]**)) OR (chimerization**[Title/Abstract]**)) OR (chimerized**[Title/Abstract]**)) OR (chimers**[Title/Abstract]**)** |
| #2 | **(((((((((antigen**[Title/Abstract]**) OR (antigens**[Title/Abstract]**)) OR (antigene**[Title/Abstract]**)) OR (antigenes**[Title/Abstract]**)) OR (antigenic**[Title/Abstract]**)) OR (antigenically**[Title/Abstract]**)) OR (antigenicities**[Title/Abstract]**)) OR (antigenicity**[Title/Abstract]**)) OR (antigenized**[Title/Abstract]**)) OR (anti-gens**[Title/Abstract]**)** |
| #3 | **((receptor-modified**[Title/Abstract]**)) OR (receptor-transduced**[Title/Abstract]**)** |
| #4 | **(((((T lymphocyte**[Title/Abstract]**) OR (T cell**[Title/Abstract]**)) OR (T cells**[Title/Abstract]**)) OR (T lymphocytes**[Title/Abstract]**)) OR (Thymus Dependent Lymphocytes**[Title/Abstract]**)) OR (CAR-T**[Title/Abstract]**)** |
| #5 | #1 AND #2 AND #3 AND #4 |
| #6 | "Immunotherapy, Adoptive"[Mesh] |
| #7 | (((((((Chimeric Antigen Receptor Therapy[Title/Abstract]) OR (CAR T-Cell Therapy[Title/Abstract])) OR (CAR T Cell Therapy[Title/Abstract])) OR (CAR T-Cell Therapies[Title/Abstract])) OR (T-Cell Therapies, CAR[Title/Abstract])) OR (T-Cell Therapy, CAR[Title/Abstract])) OR (Therapies, CAR T-Cell[Title/Abstract])) OR (Therapy, CAR T-Cell[Title/Abstract]) |
| #8 | #5 OR #6 OR #7 |
| #9 | "Multiple Myeloma"[Mesh] |
| #10 | ((((((((((((((((((Multiple Myelomas[Title/Abstract]) OR (Myeloma*, Multiple[Title/Abstract])) OR (Myeloma, Plasma-Cell[Title/Abstract])) OR (Myeloma, Plasma Cell[Title/Abstract])) OR (Myelomas, Plasma-Cell[Title/Abstract])) OR (Plasma-Cell Myeloma[Title/Abstract])) OR (Plasma-Cell Myelomas[Title/Abstract])) OR (Myelomatosis[Title/Abstract])) OR (Myelomatoses[Title/Abstract])) OR (Plasma Cell Myeloma[Title/Abstract])) OR (Cell Myeloma, Plasma[Title/Abstract])) OR (Cell Myelomas, Plasma[Title/Abstract])) OR (Myelomas, Plasma Cell[Title/Abstract])) OR (Plasma Cell Myelomas[Title/Abstract])) OR (Kahler Disease[Title/Abstract])) OR (Disease, Kahler[Title/Abstract])) OR (Myeloma-Multiple[Title/Abstract])) OR (Myeloma Multiple[Title/Abstract])) OR (Myeloma-Multiples[Title/Abstract]) |
| #11 | #9 OR #10 |
| #12 | (clinical[Title/Abstract] AND trial[Title/Abstract]) OR clinical trials as topic[MeSH Terms] OR clinical trial[Publication Type] OR random*[Title/Abstract] OR random allocation[MeSH Terms] OR therapeutic use[MeSH Subheading] |
| #13 | #8 AND #11 AND #12 |

Supplemental Table 2: Abbreviation index

| Abbreviations | Full names |
| --- | --- |
| CAR-T | Chimeric antigen receptor T cells |
| B-ALL | B-lymphoblastic leukemia |
| r/r B-ALL | Refractory/recurrent acute B-lymphoblastic leukemia |
| DLBCL | Diffuse large B-cell lymphoma |
| R/R DLBCL | Refractory/Relapsed Diffuse large B-cell lymphoma |
| MM | Multiple myeloma |
| RRMM | Refractory/relapsed multiple myeloma |
| NHL | Non-Hodgkin Lymphoma |
| OR | Odds Ratio |
| RR | Relative Risk |
| 95％CI | 95％ Confidence Interval |
| ORR | Overall response rate |
| CRR | Complete remission rate |
| sCR | Stringent complete remission |
| CR | Complete response |
| VGPR | Very good partial response |
| PR | Partial response |
| PFS | Progression free survival |
| OS | Overall survival |
| RL | Relapse rate |
| CRS | Cytokine-release syndrome |
| CRES | CAR-T cell related encephalopathy syndrome |
| TLS | Tumor Lysis Syndrome |
| HSCT | Hematopoietic stem cell transplantation |
| scFv | Single-chain variable fragment |
| MHC | Major histocompatibility complex |
| MINORS | Methodological index for nonrandomized studies |
| NOS | The Newcastle-Ottawa scale |
| FDA | Food and Drug Administration |
| CNKI | China National Knowledge Infrastructure |

Supplemental Figure 1. Pooled rate of (a) strict complete response (sCR), (b) complete response (CR), (c) very good partial response (VGPR), (d) partial response (PR), (e) median progression-free survival (PFS), (f) median overall survival (OS), and (g) median duration of response (DOR) among RRMM.

**a.**


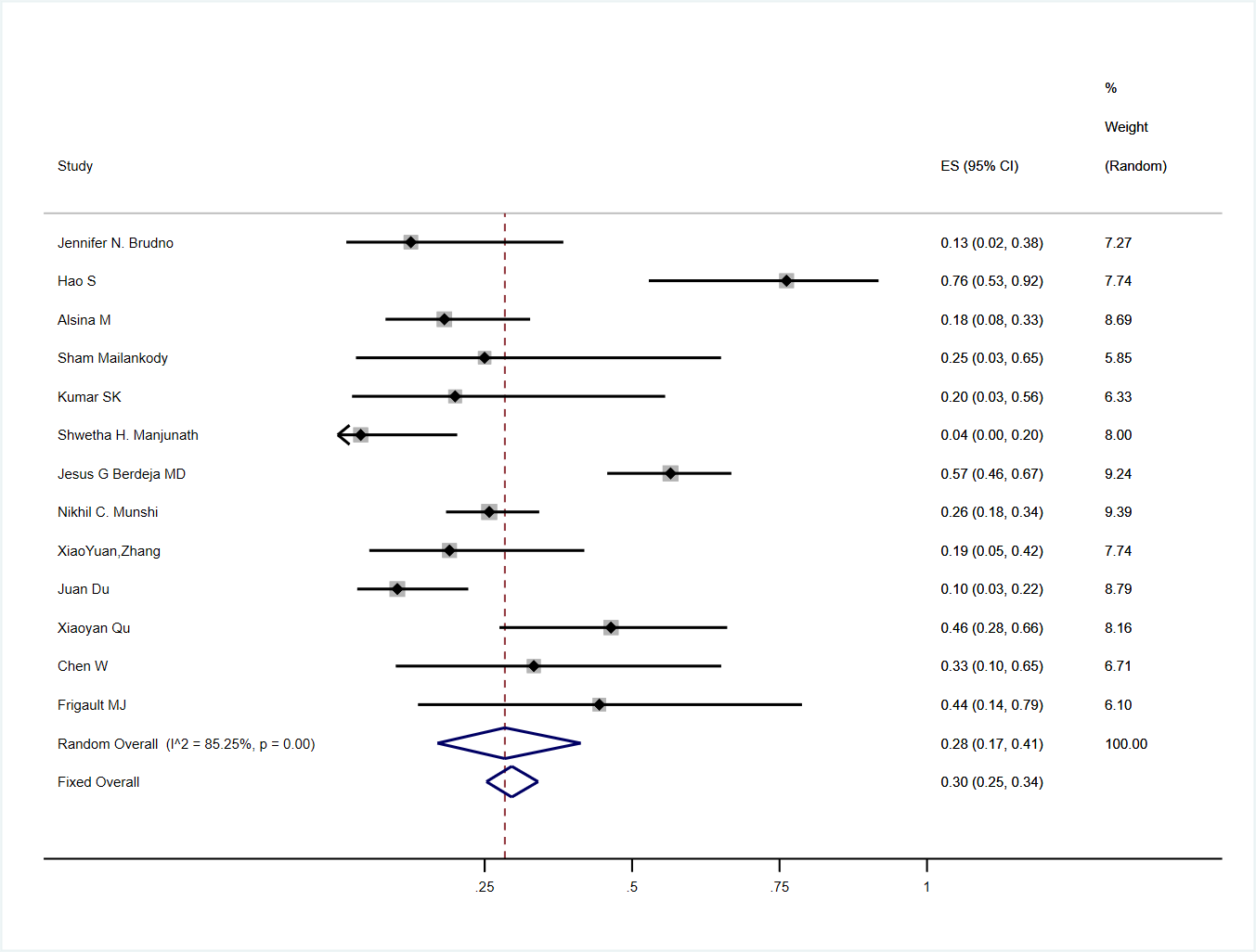


**b.**


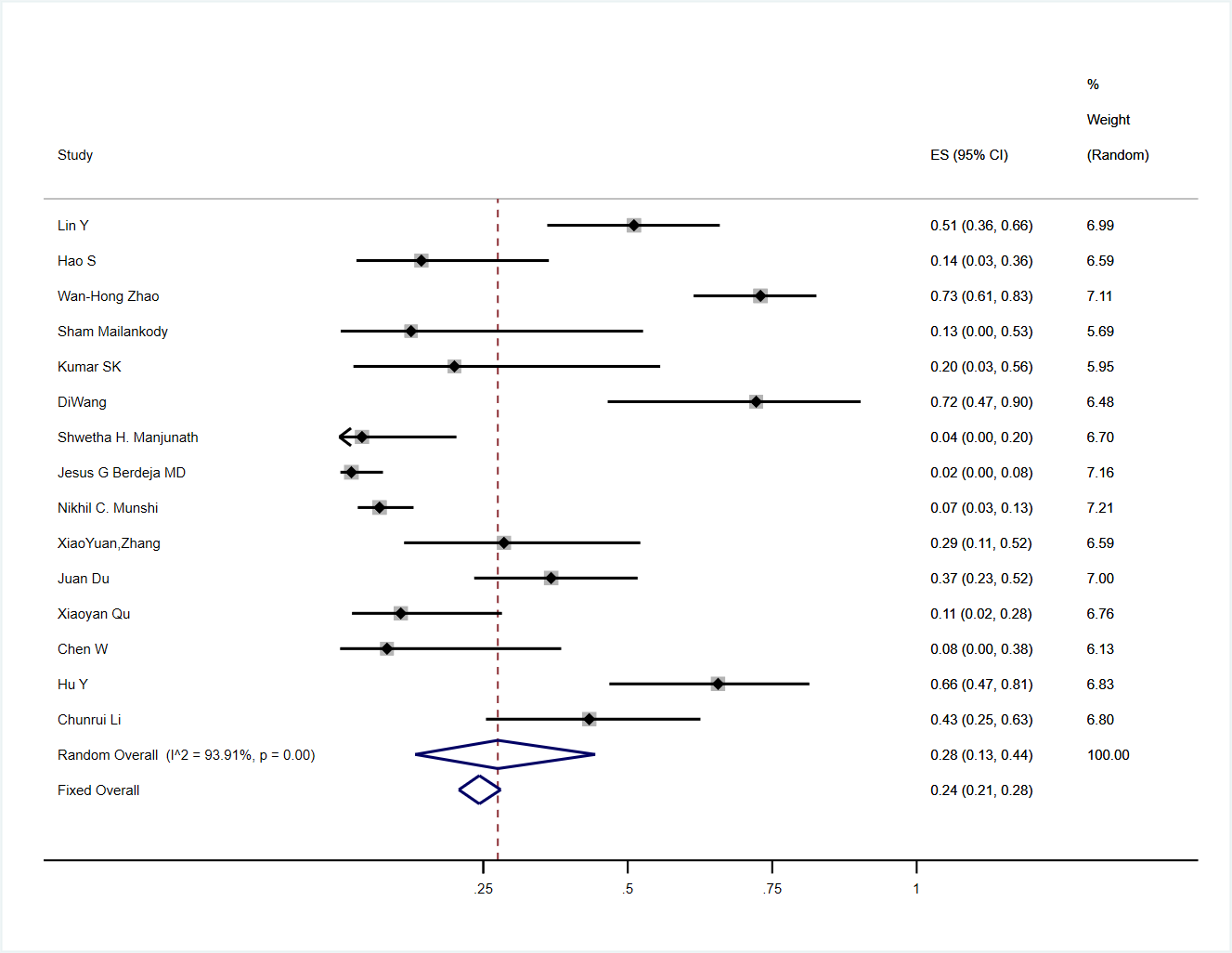


**c.**


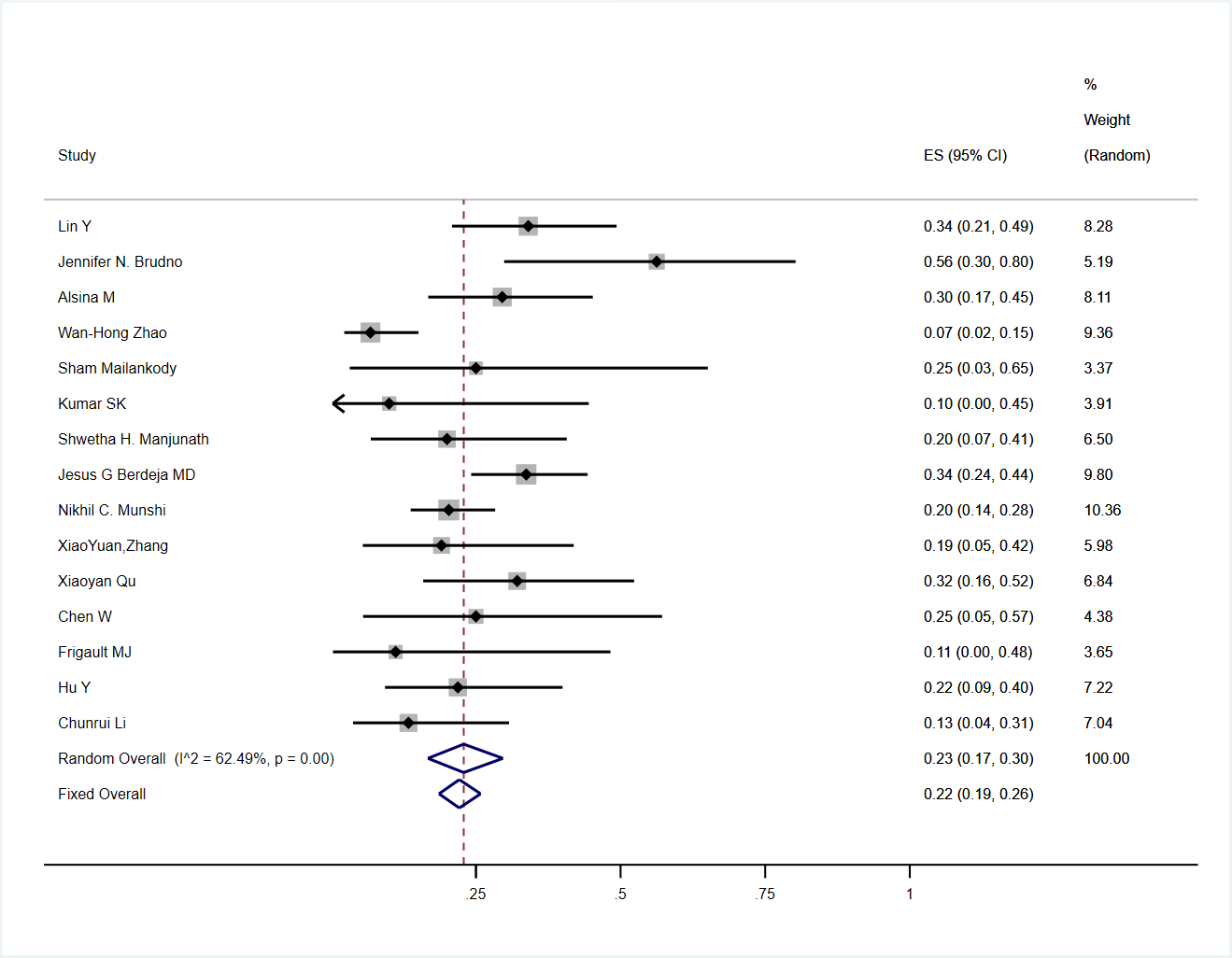


**d.**


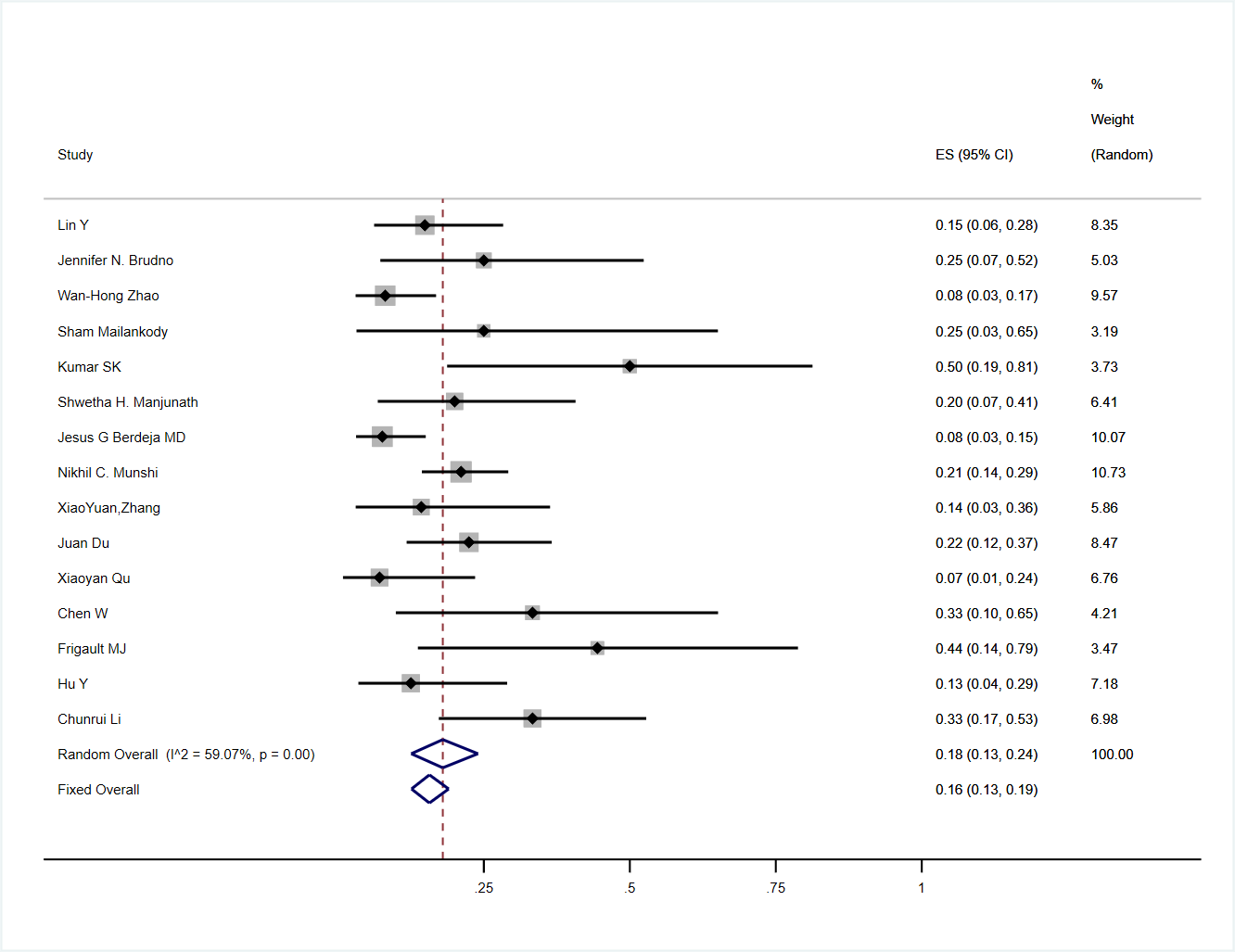


**e.**

**
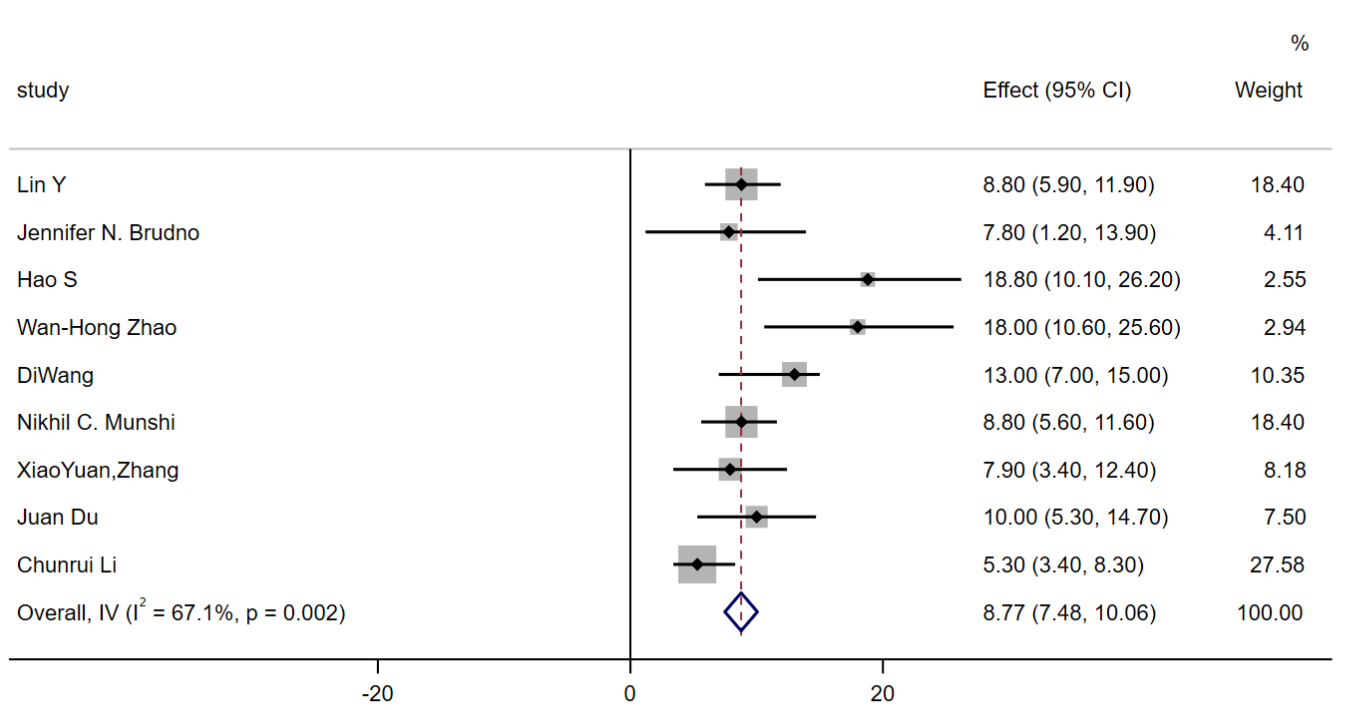
**

**f.**

**
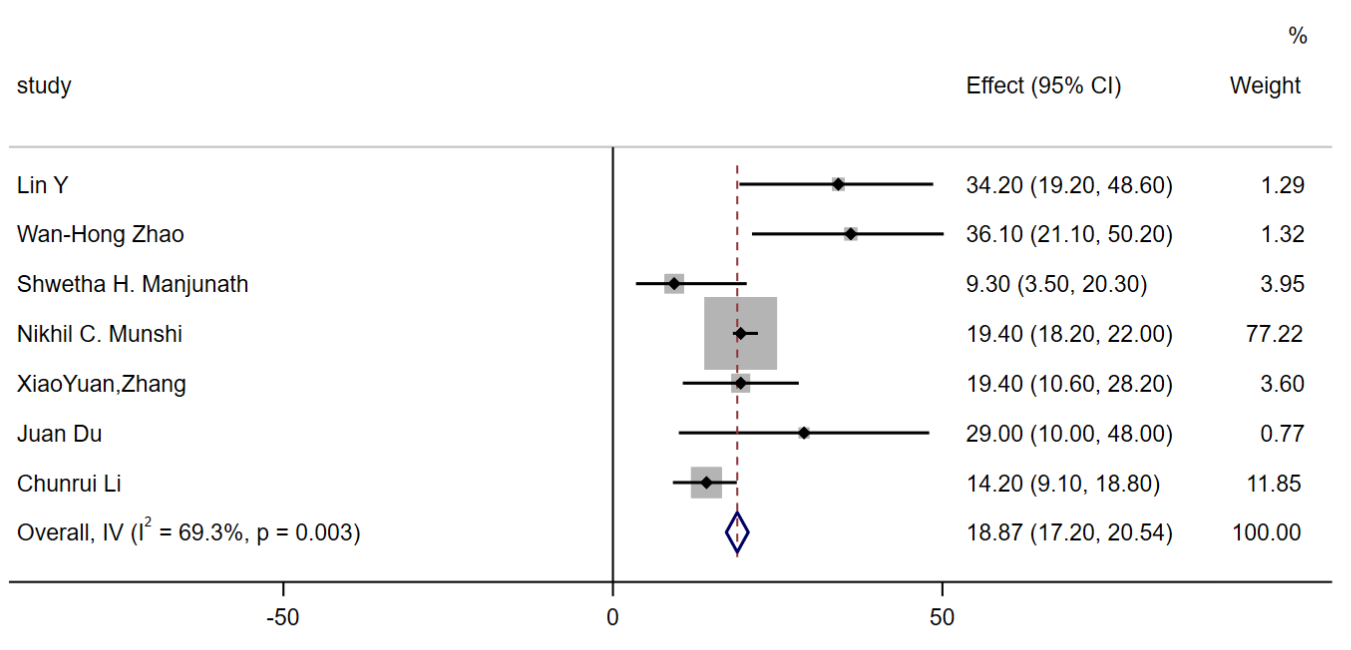
**

**g.**

**
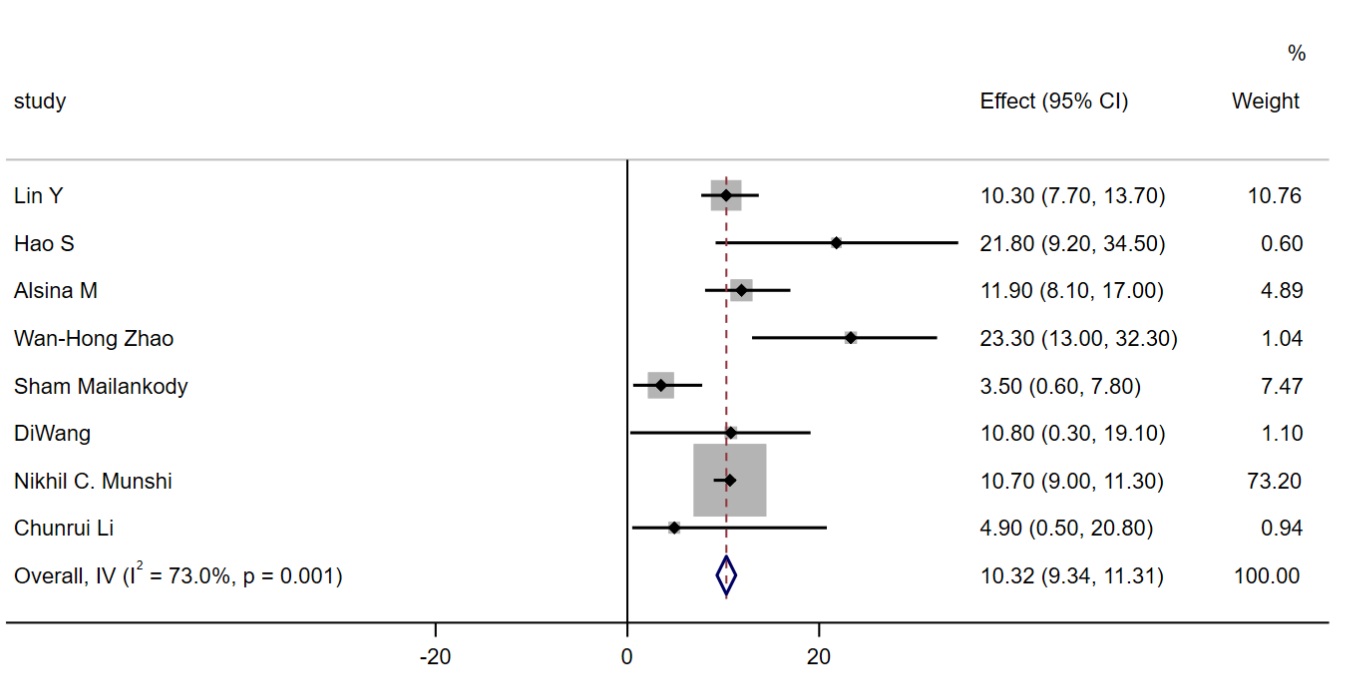
**

Supplemental Figure 2. Pooled rate of (a) grade≥3 cytokine release syndrome and (b) grade≥3 CAR-T-related encephalopathy syndrome among RRMM, (c) median CRS occurred time, (d) median CRS resolved time among RRMM, and (e) the pooled usage rate of tocilizumab.

**a.**


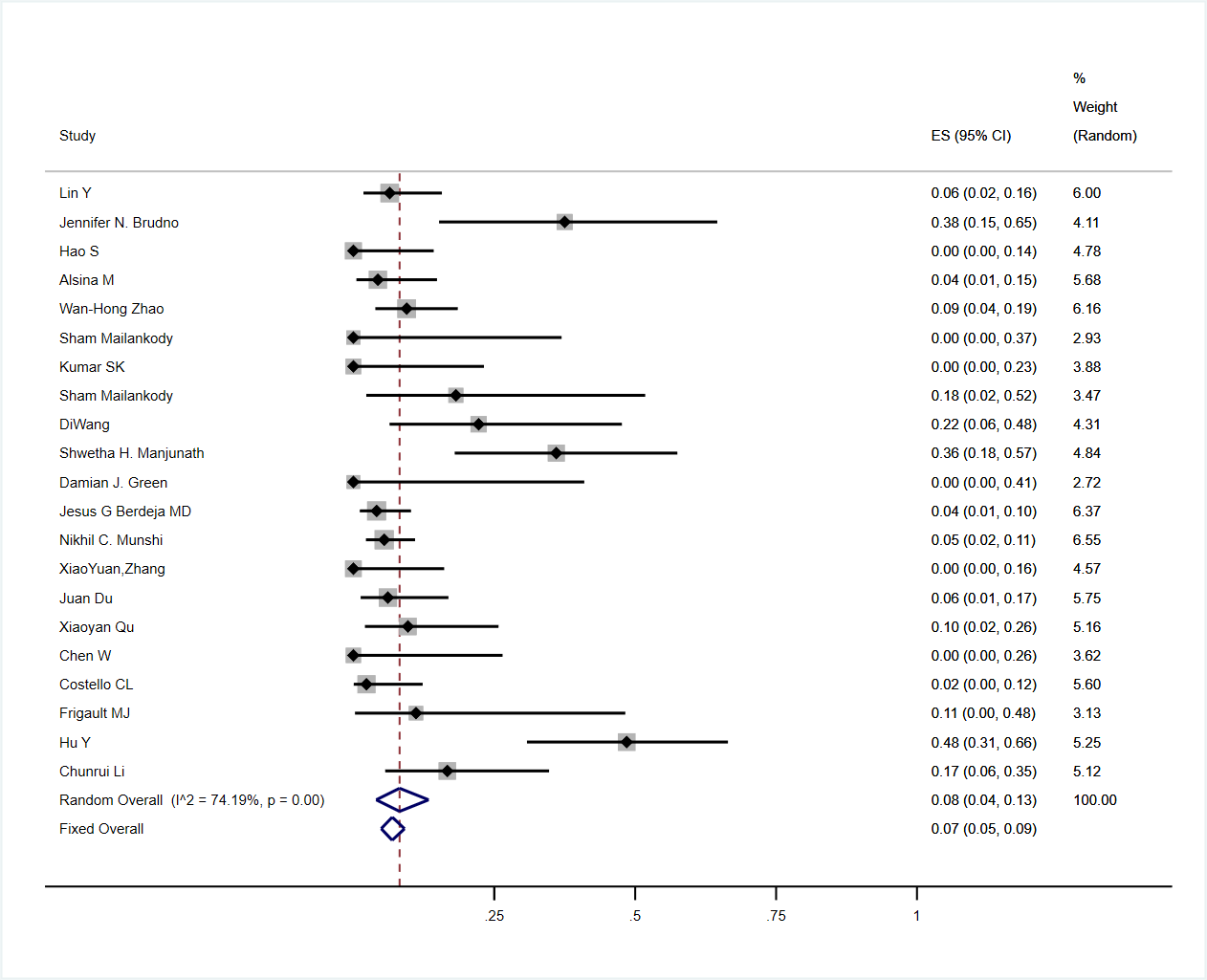


**b.**


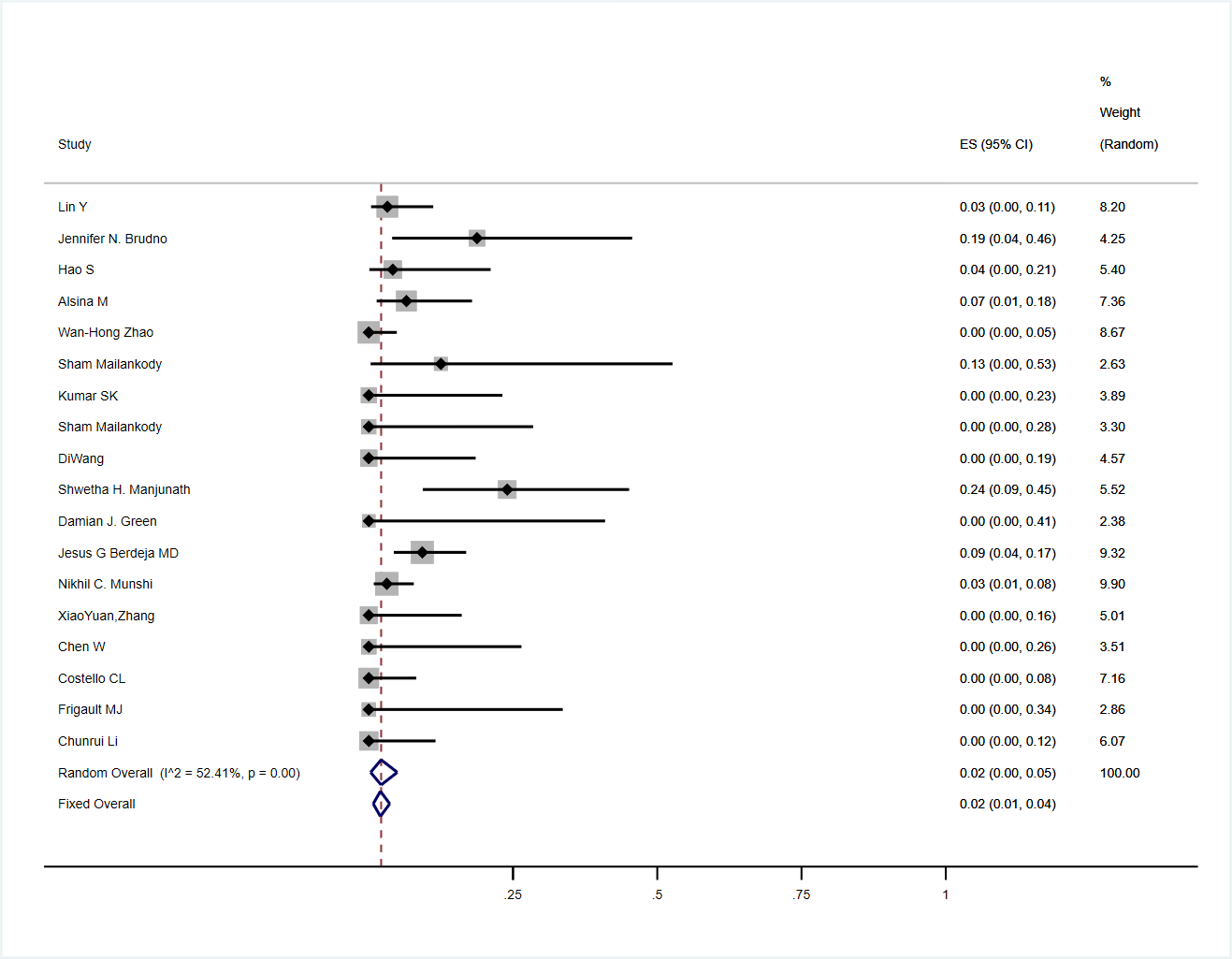


**c.**

**
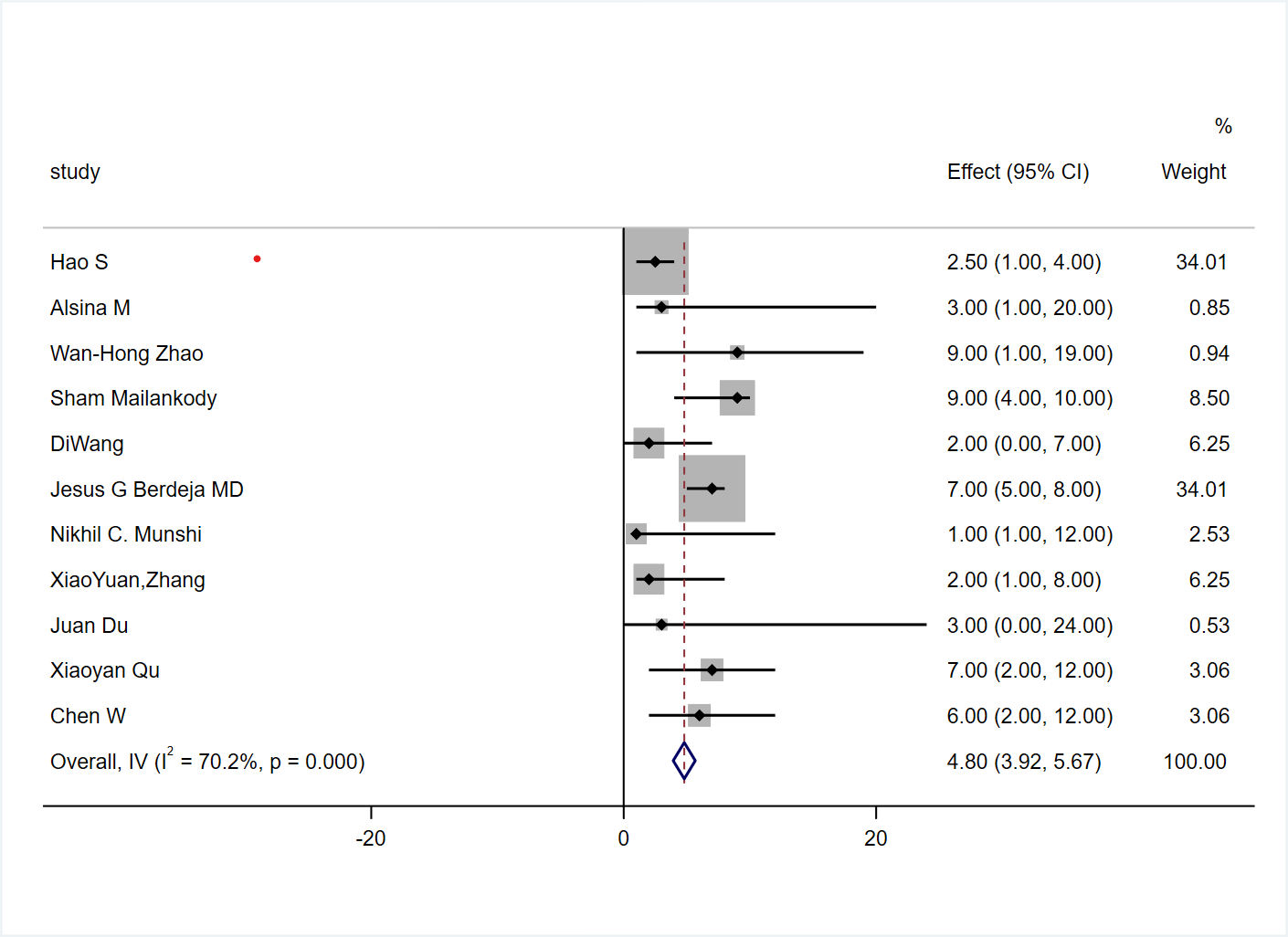
**

**d.**


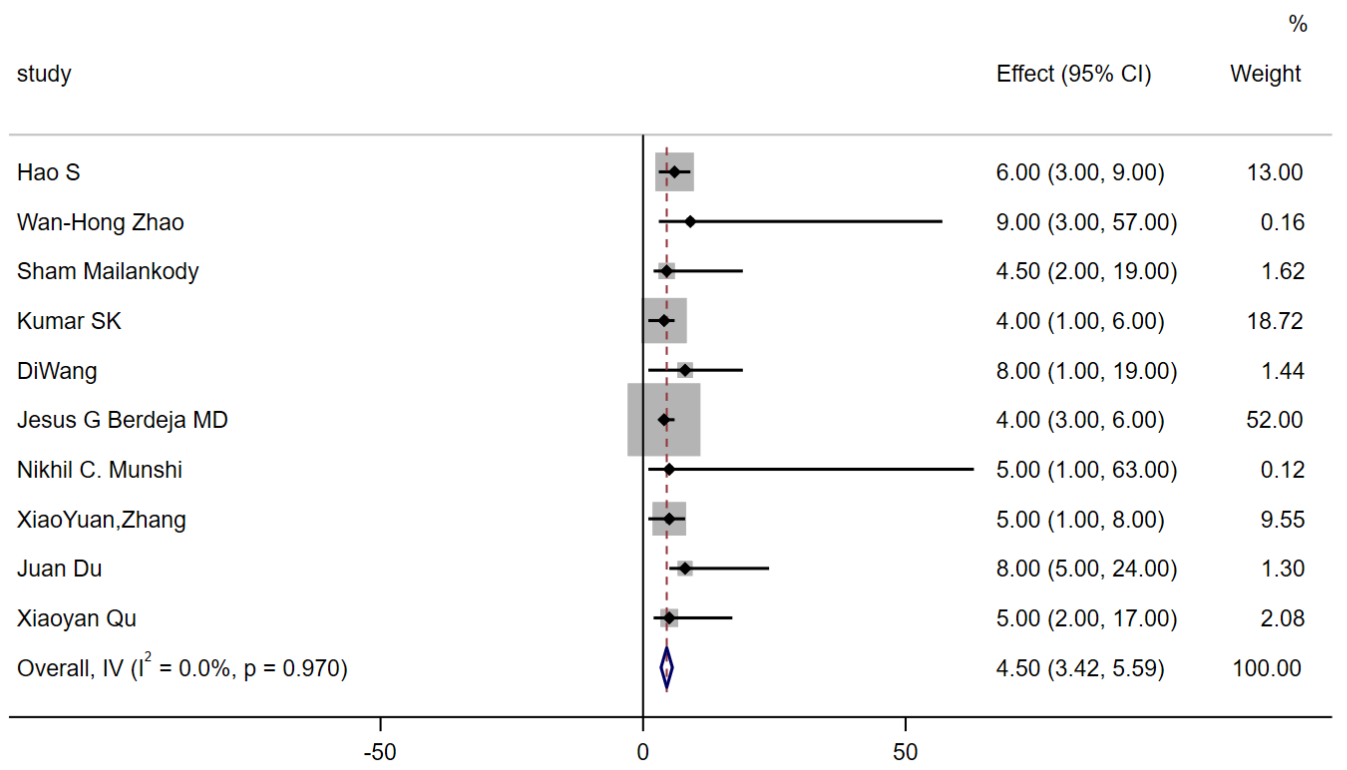


**e.**


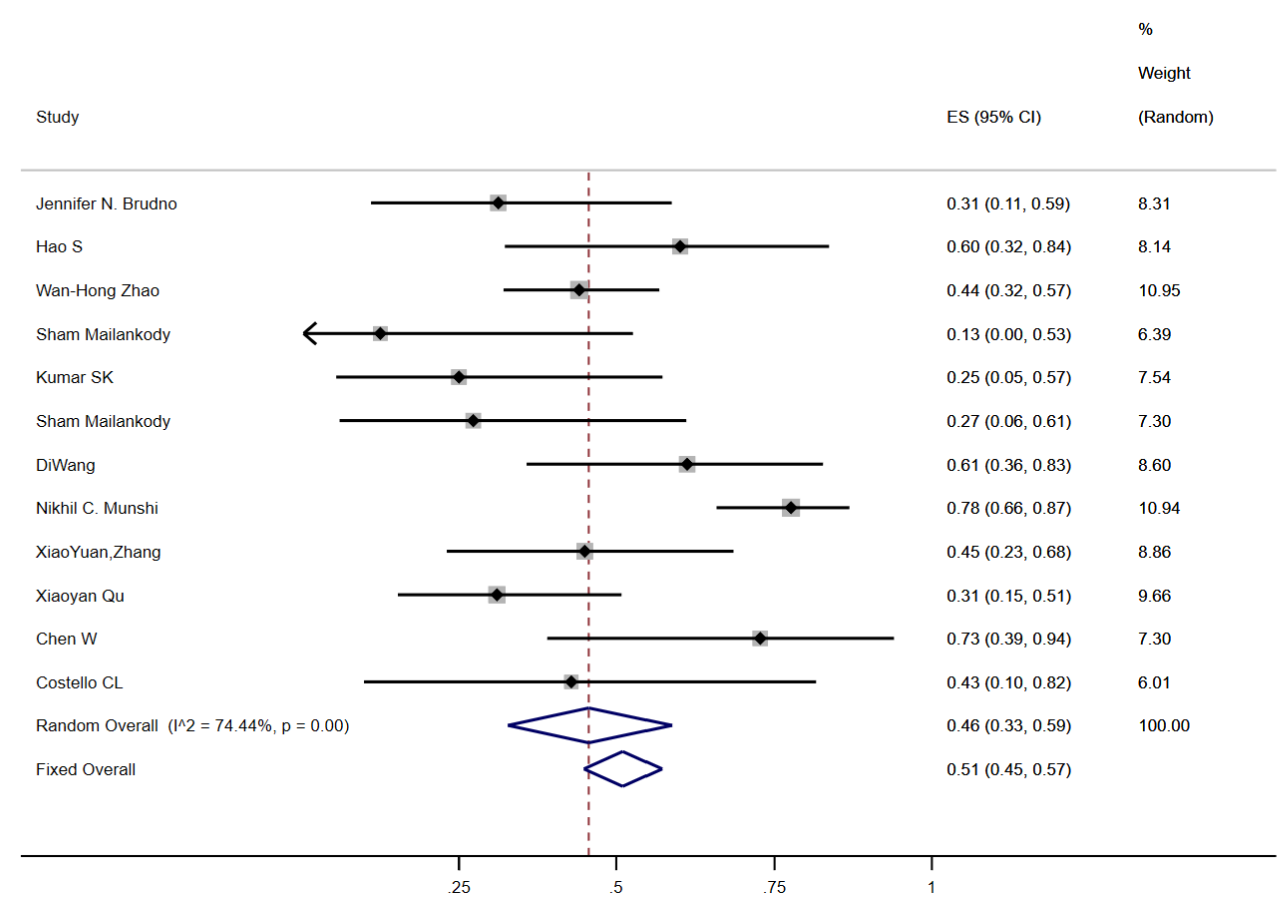


Supplemental Figure 3. Pooled rate of toxicity: (a) neutropenia, (b) anemia, (c) thrombocytopenia, (d) leukopenia, (e) lymphopenia (f) infection, and (g) fever among RRMM.

**a.**


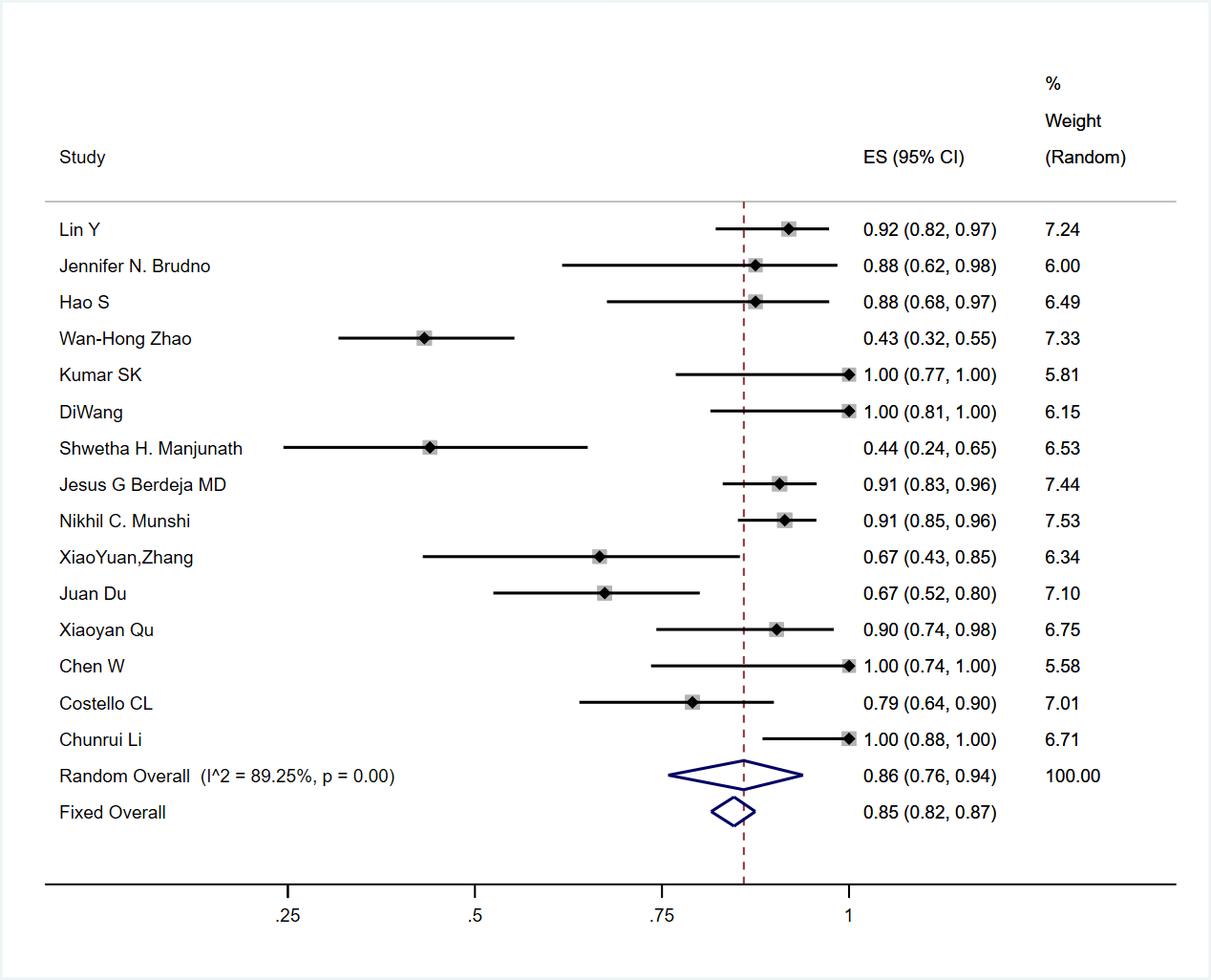


**b.**


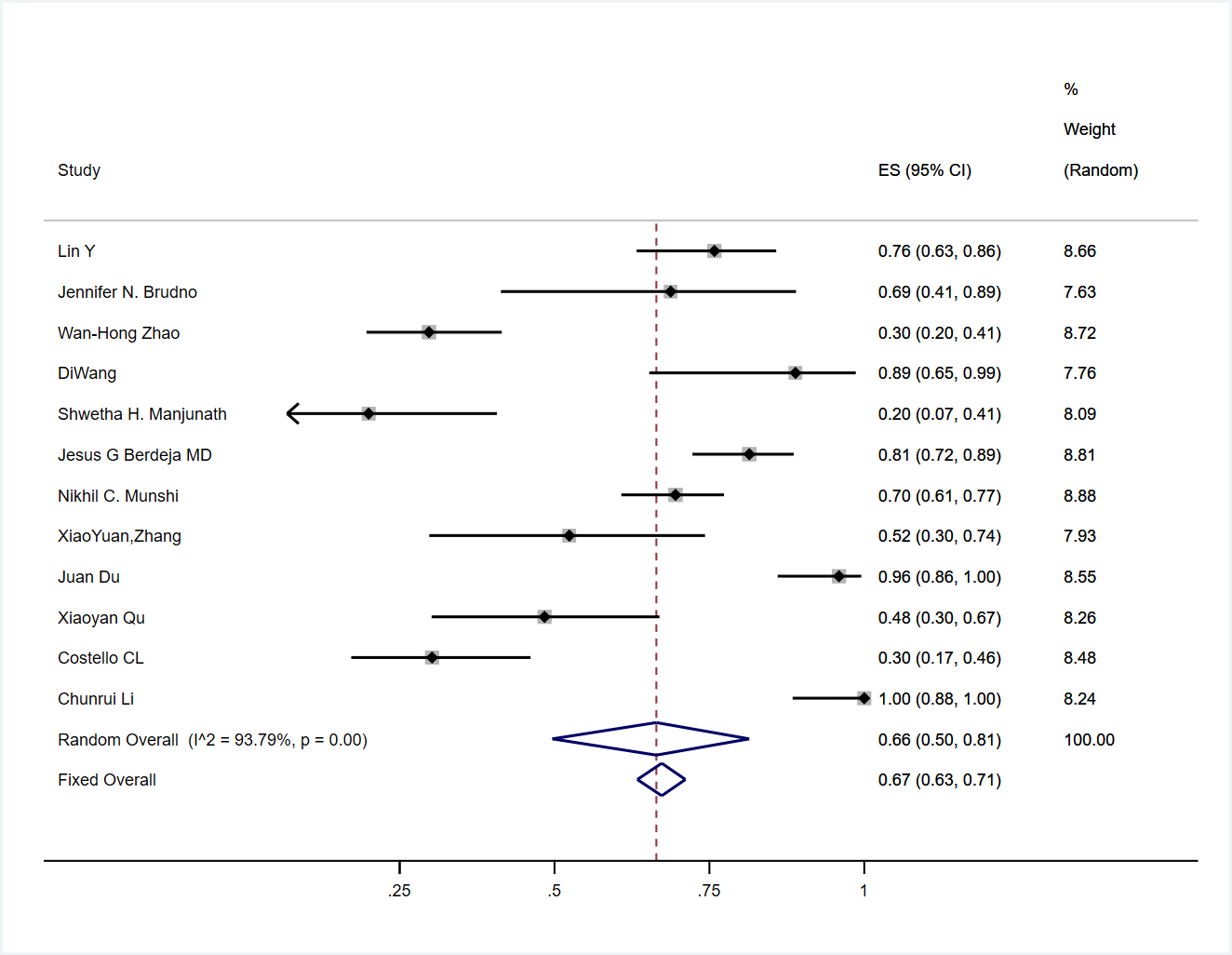


**c.**


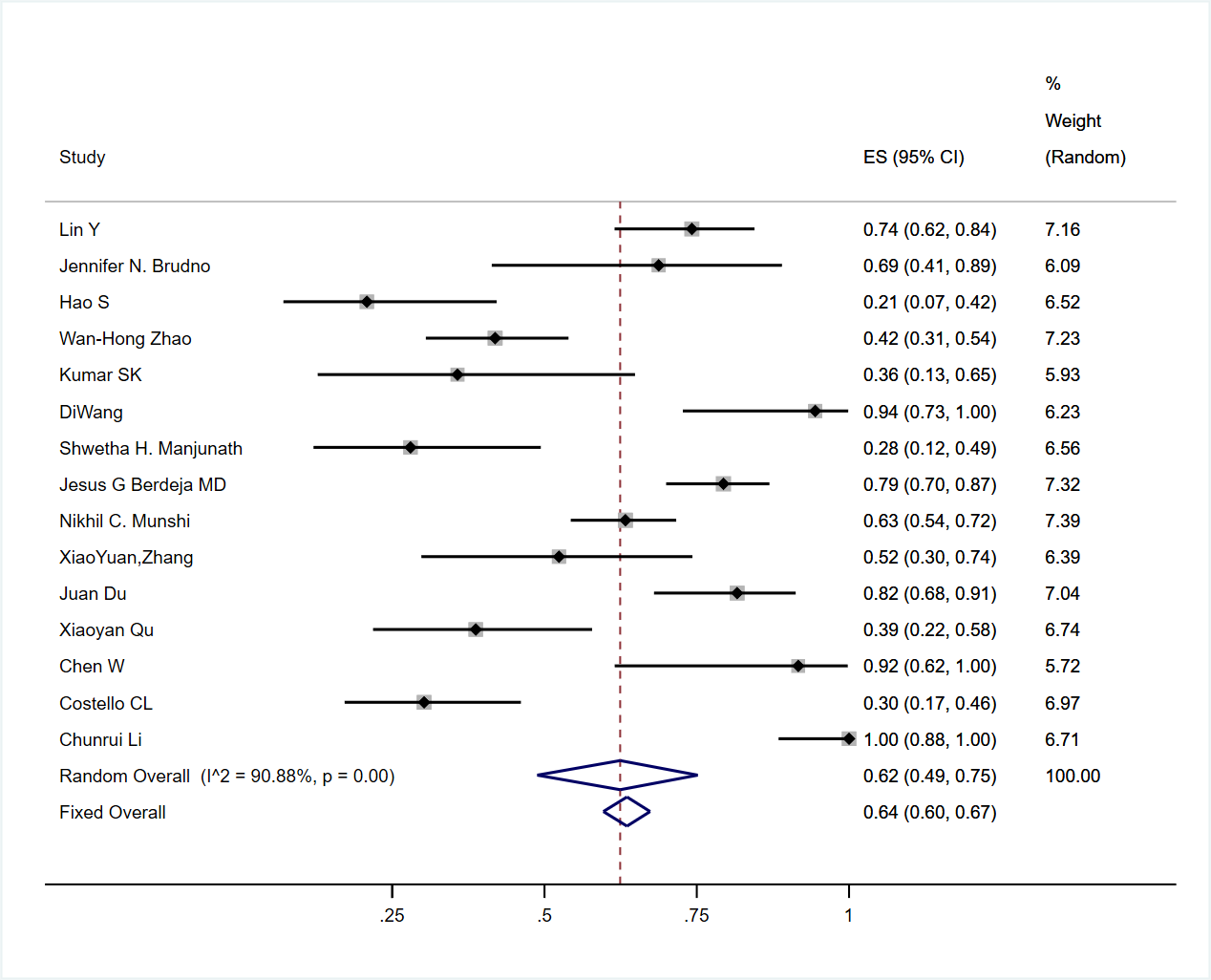


**d.**


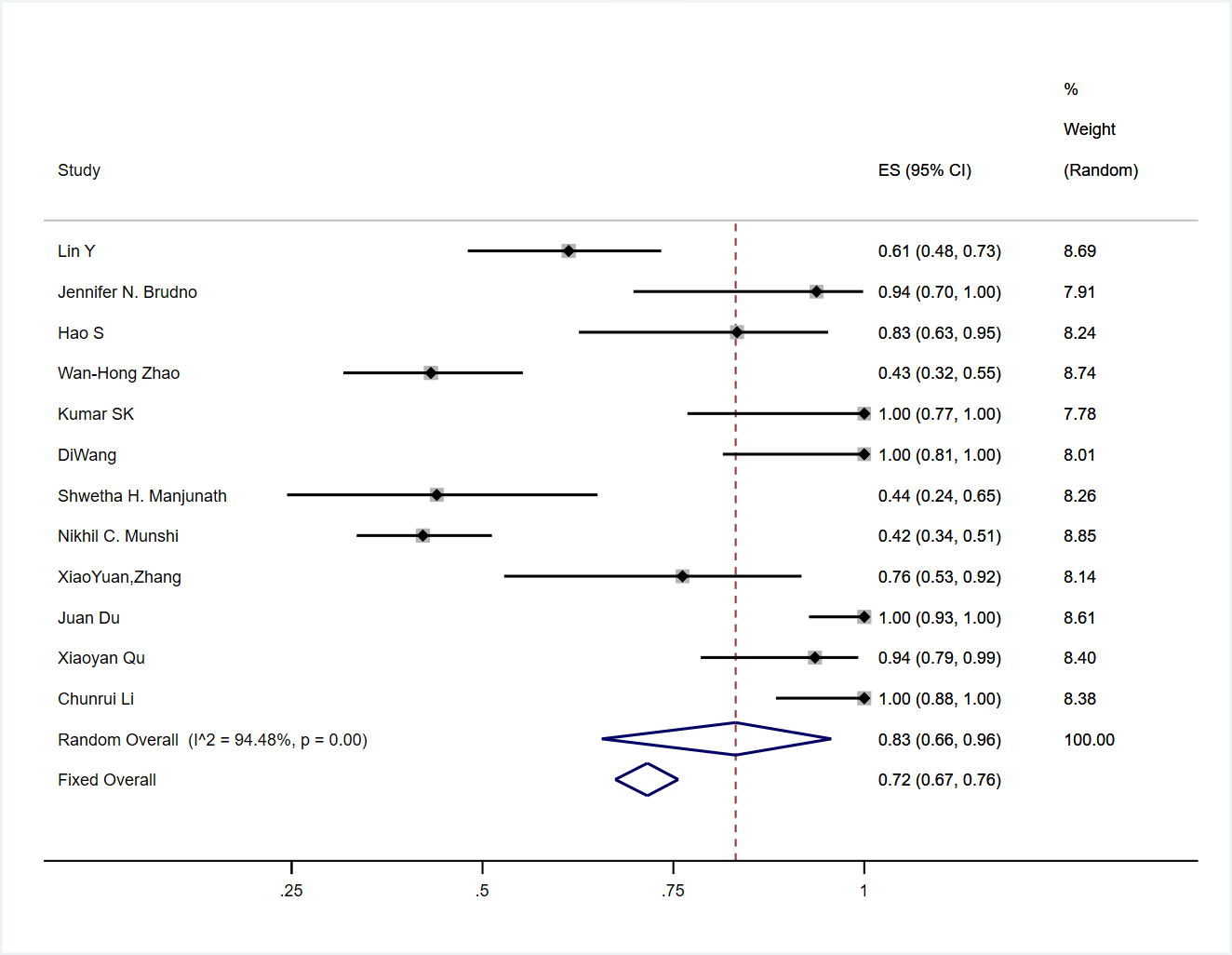


**e.**


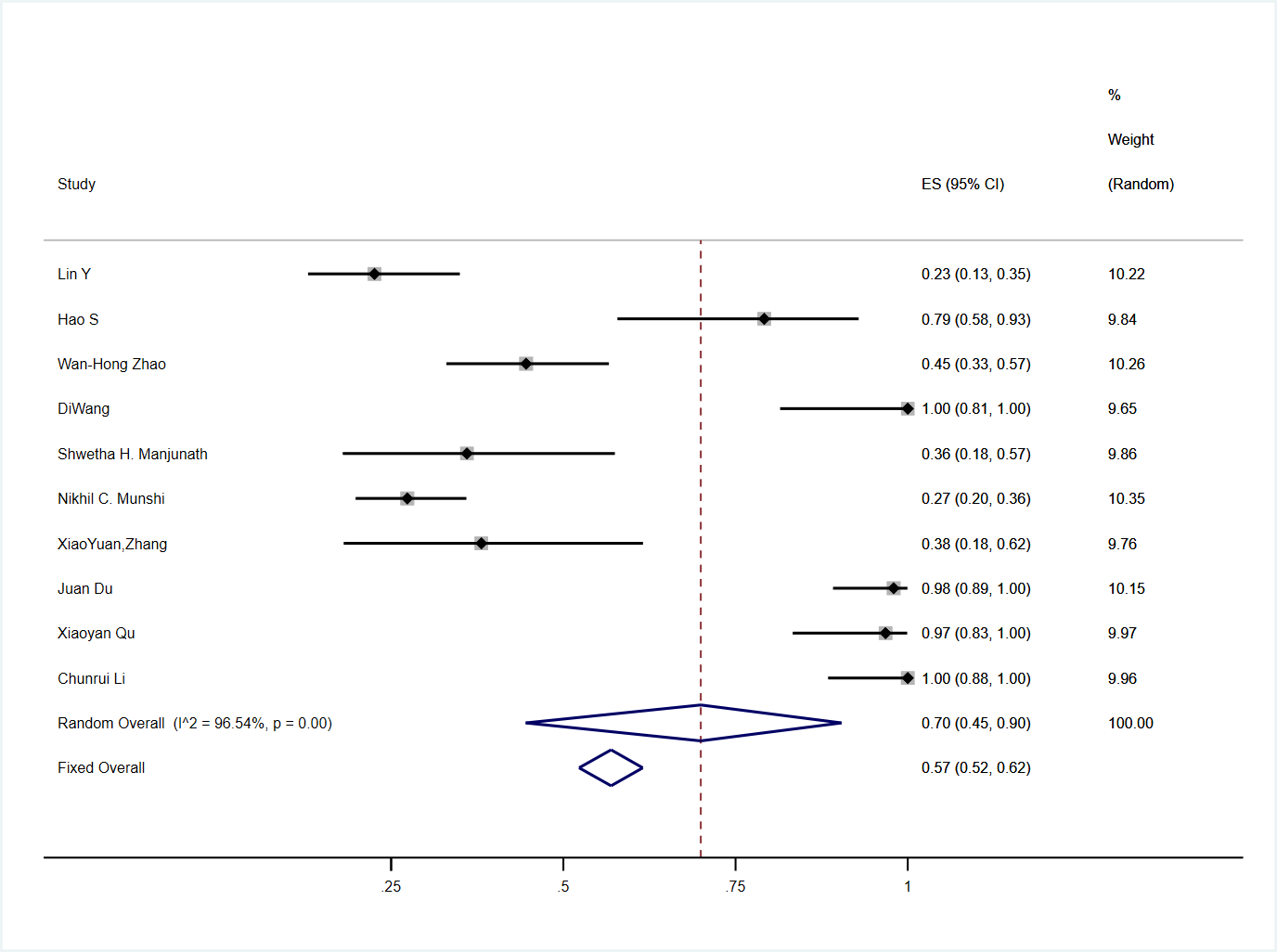


**f.**


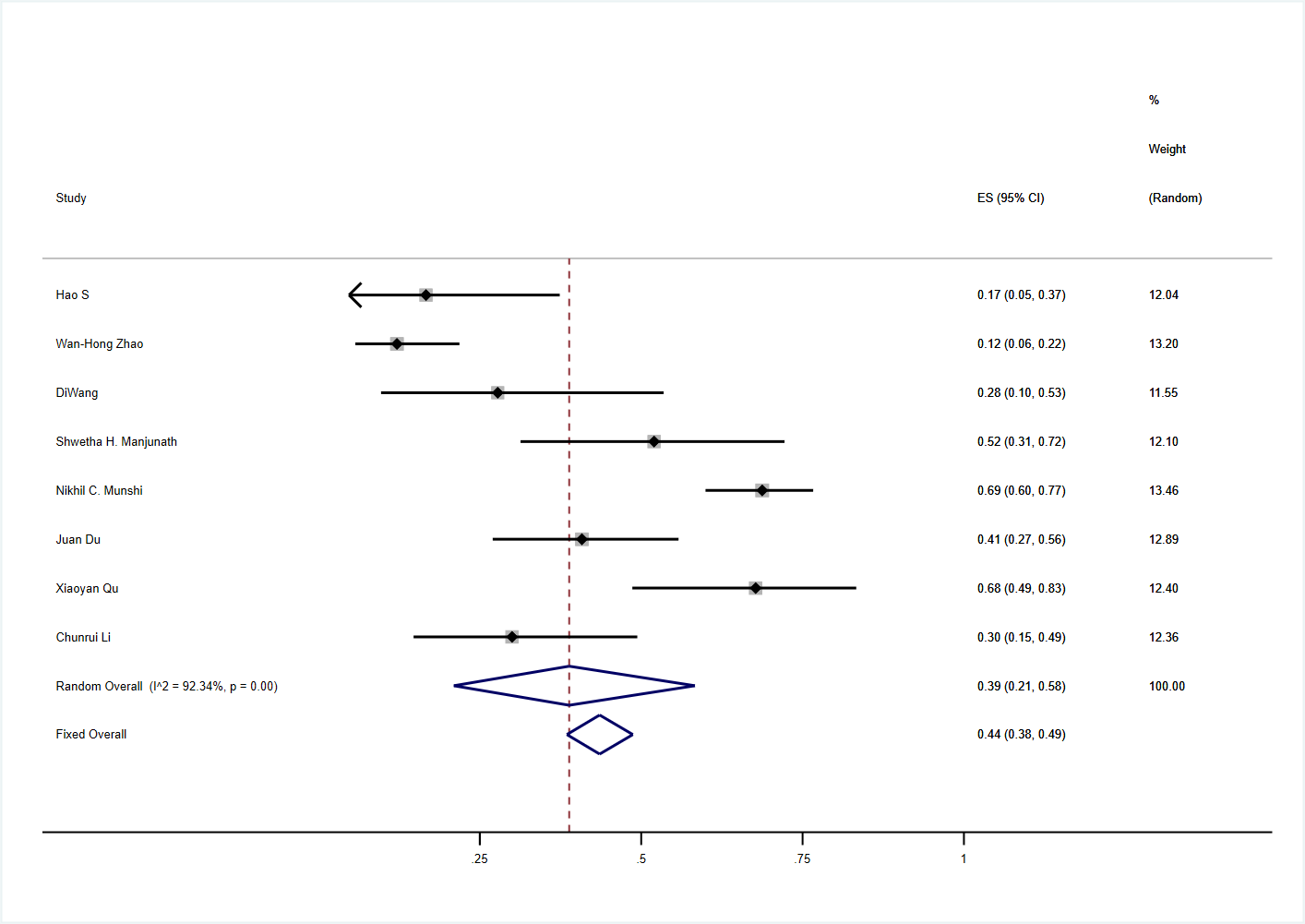


**g.**


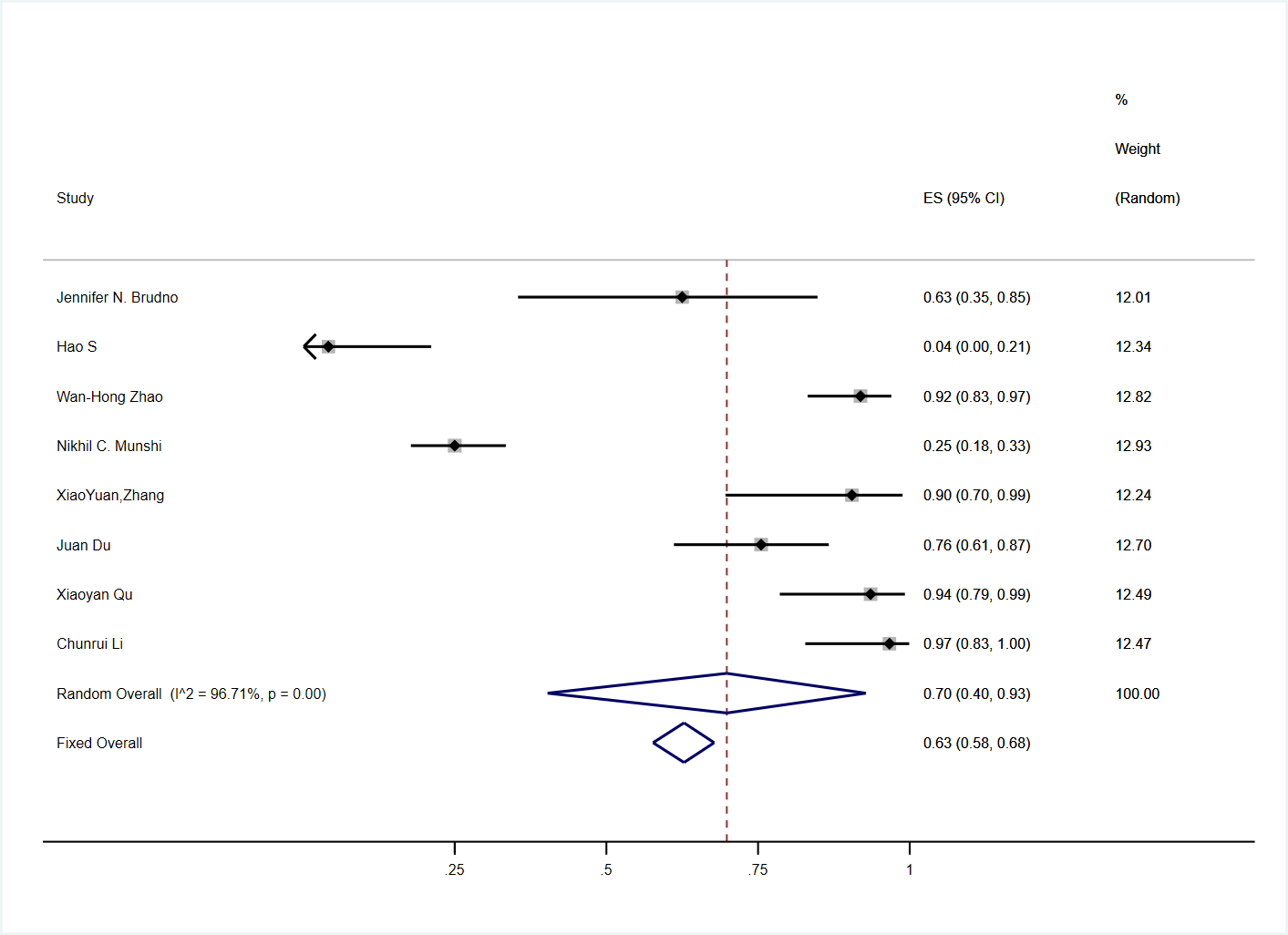


Supplemental Figure 4. Pooled (a) CAR-BCMA T cell expansion time and (b) CAR-BCMA T cell reached peak value time among RRMM.

**a.**


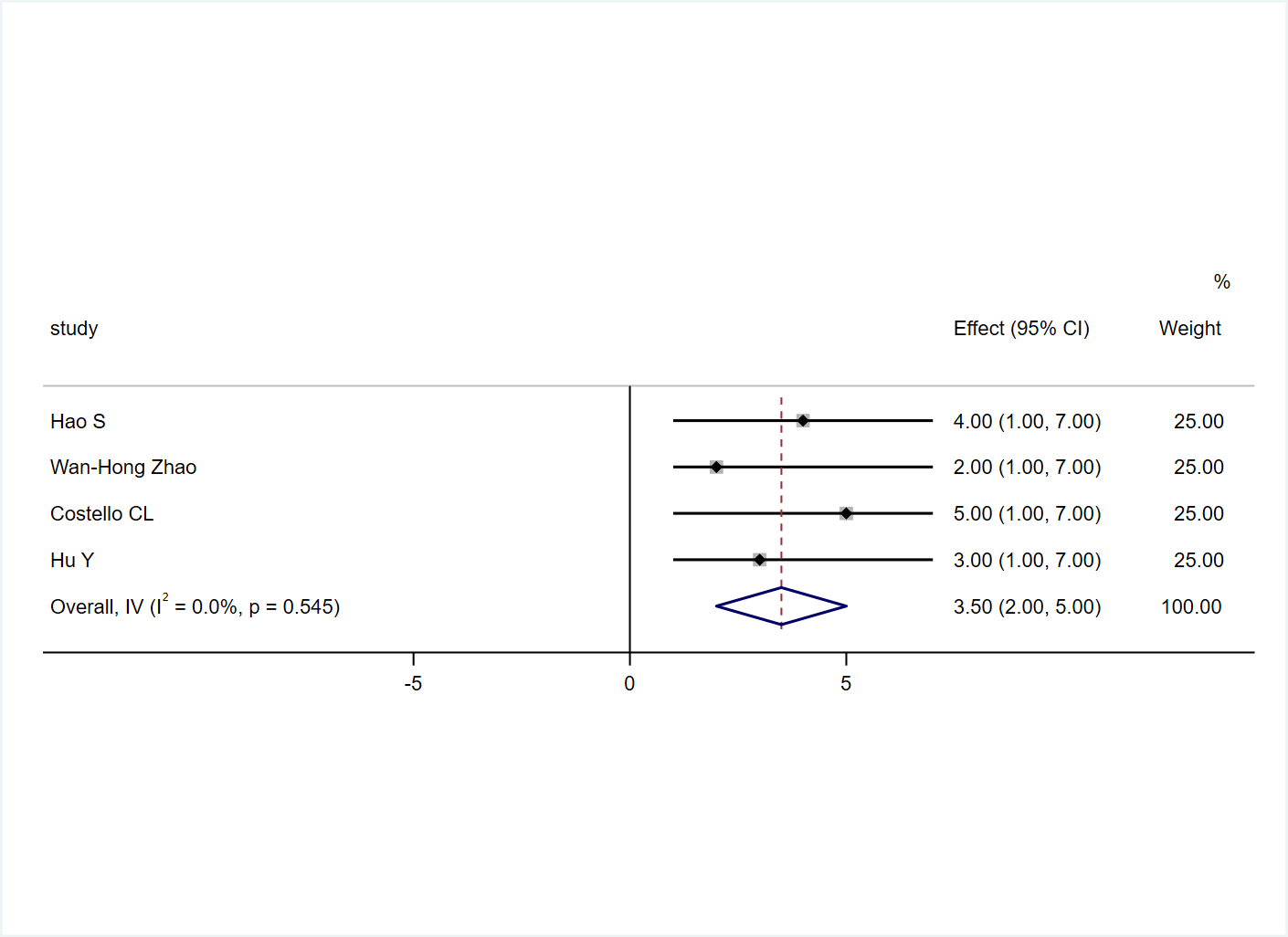


**b.**


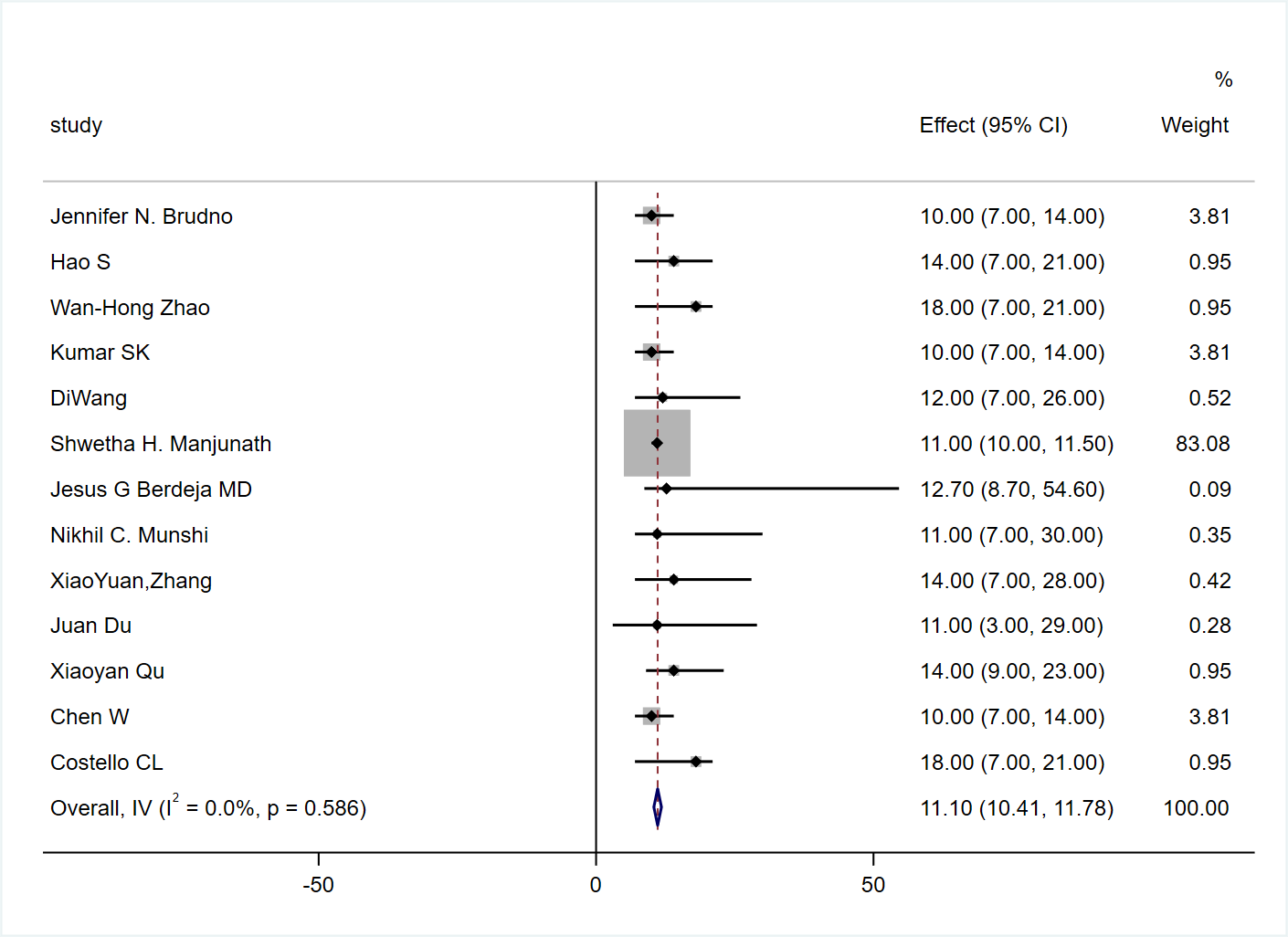


Supplemental Figure 5. Subgroup analyses of objective response rate by (a) age (＜55 vs. ≥55 years), (b) patient disease status: proportion of ECOG≥3 level (＜25% vs. ≥25% ), (c) patient disease status: proportion of ISS≥3 level (＜28% vs. ≥28% ), (d) proportion of previous ASCT (＜75% vs. ≥75% ), (e) lines of prior treatment (＜8 vs. ≥8), (f) dose (high dose group ≥200× 10^6cells or 5× 10^6 cells/kg vs. low dose group ＜200× 10^6cells or 5× 10^6 cells/kg), (g) costimulatory molecule (4-1BB vs. others), (h) loading (Lentiviral vs. Retrovirus), (i) median time from diagnosis (＜4 vs. ≥4 years)，(j) proportion of high-risk cytogenetics (＜48% vs. ≥48%), (k) proportion of extramedullary disease (＜29% vs. ≥29% ), (l) proportion of mAb exposed (＜39% vs. ≥39% ) among RRMM.

**a.**

**
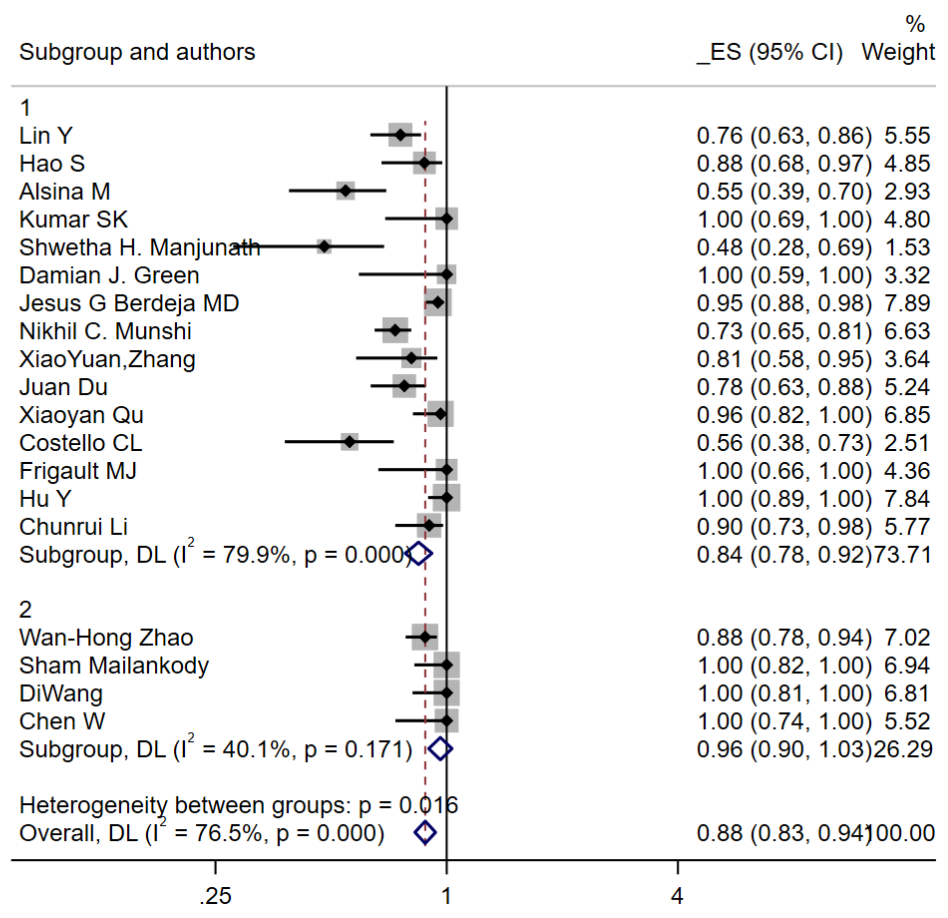
**

**b.**

**
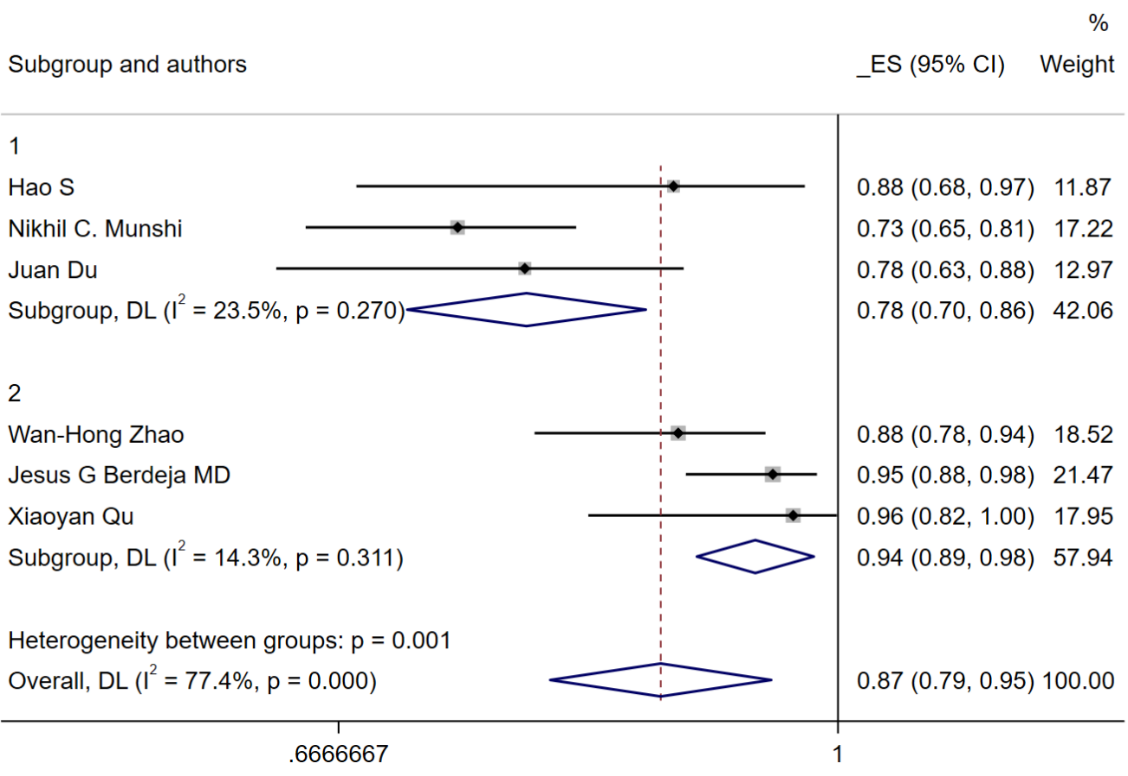
**

**c.**

**
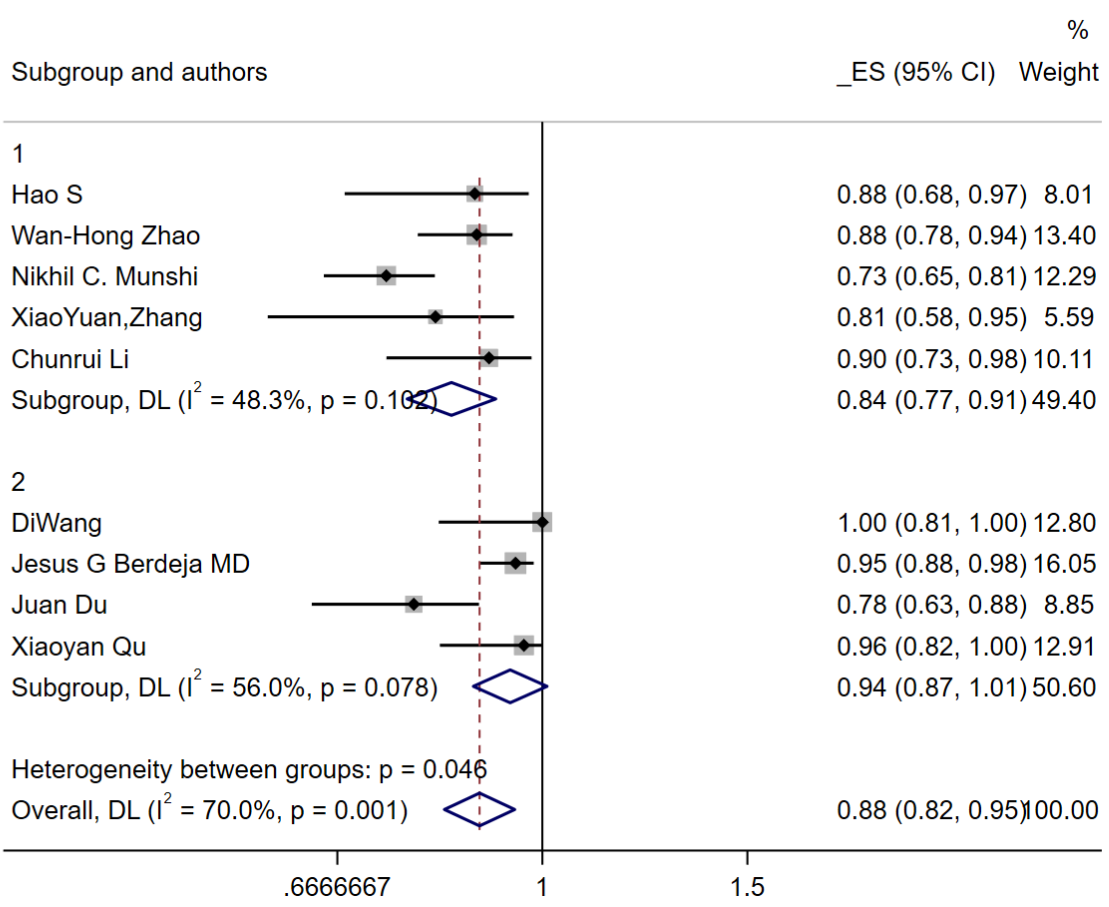
**

**d.**

**
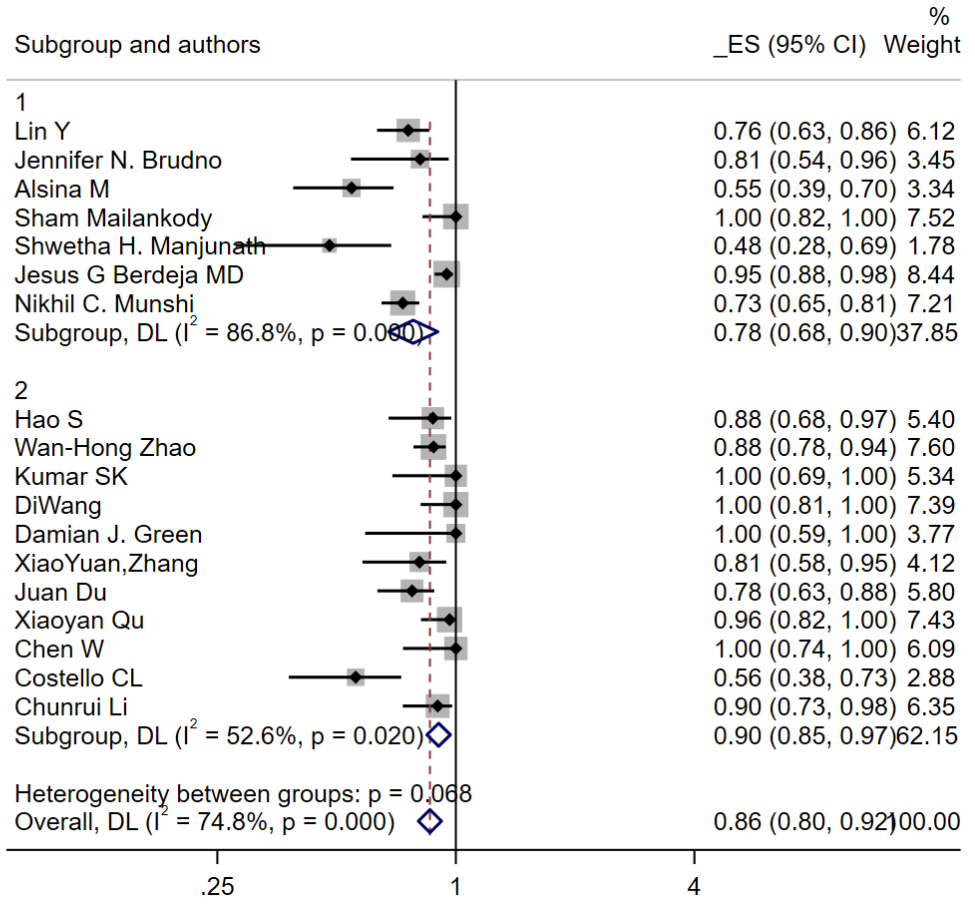
**

**e.**

**
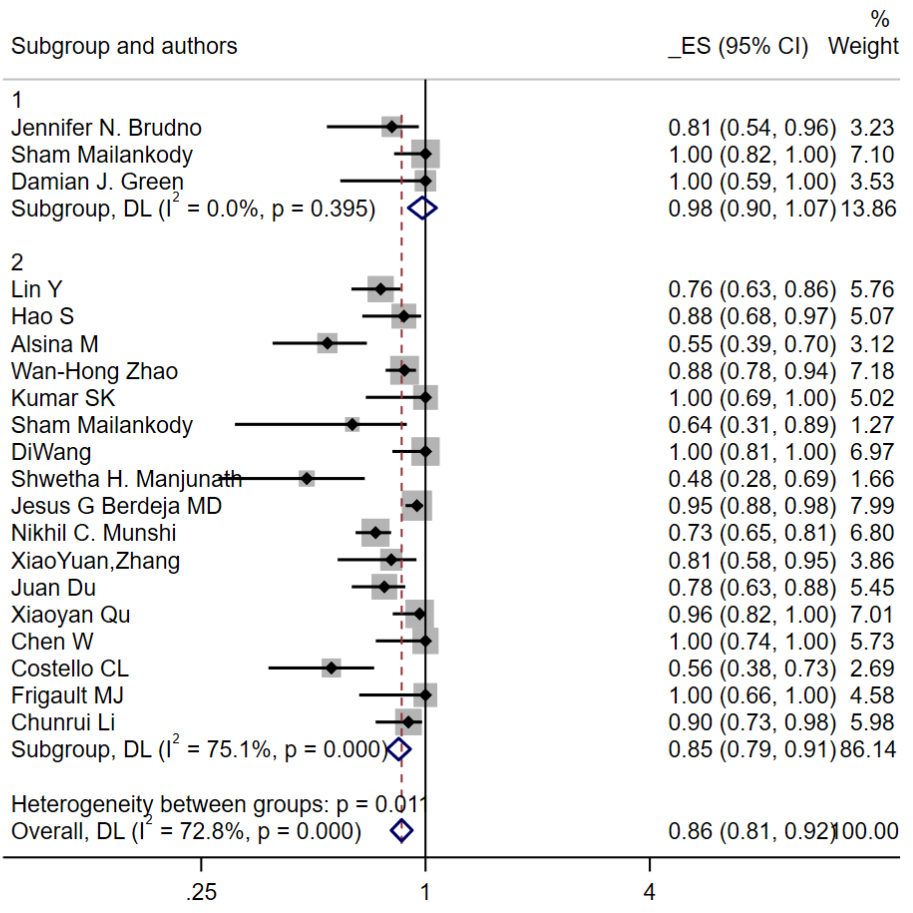
**

**f.**

**
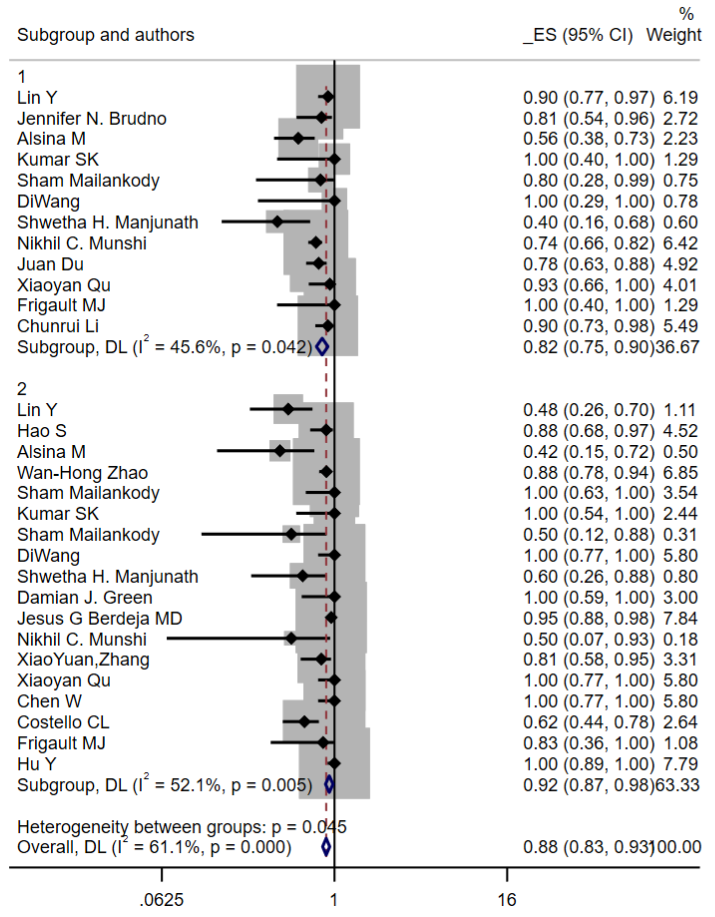
**

**g.**

**
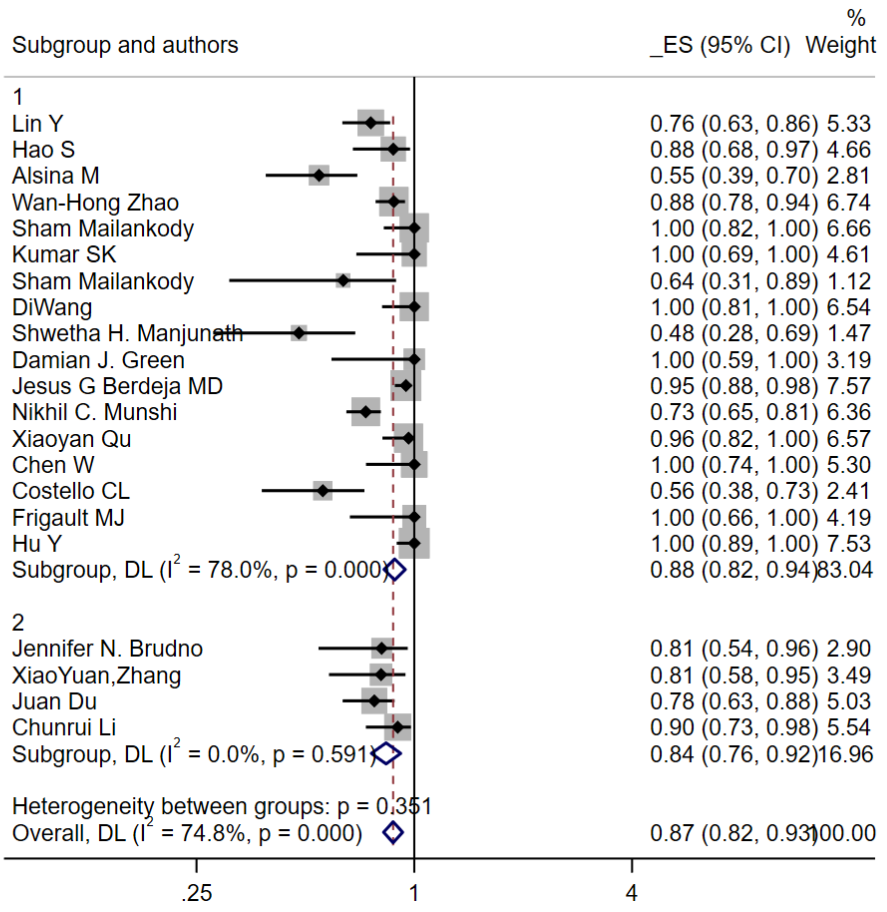
**

**h.**


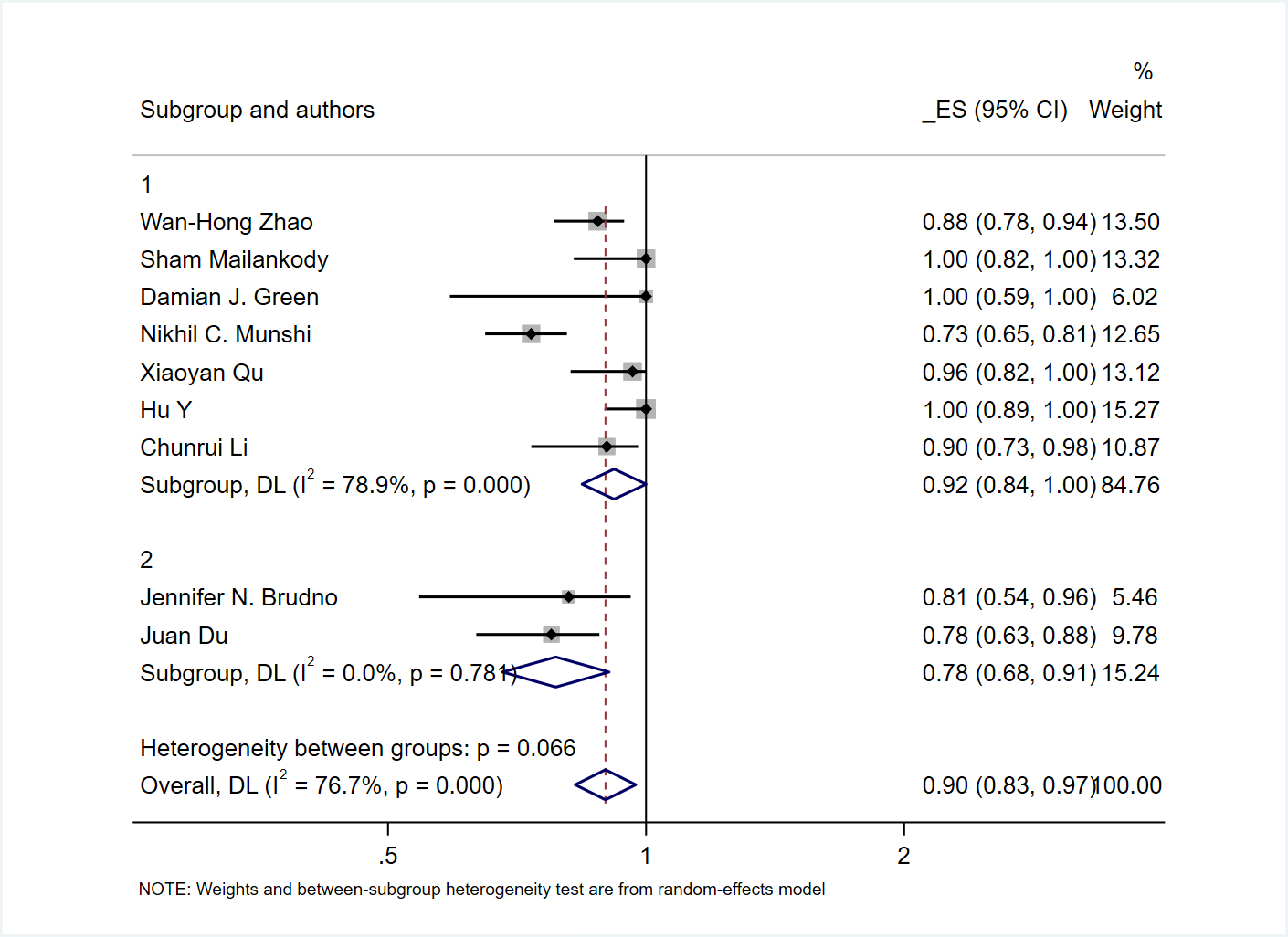


**i.**


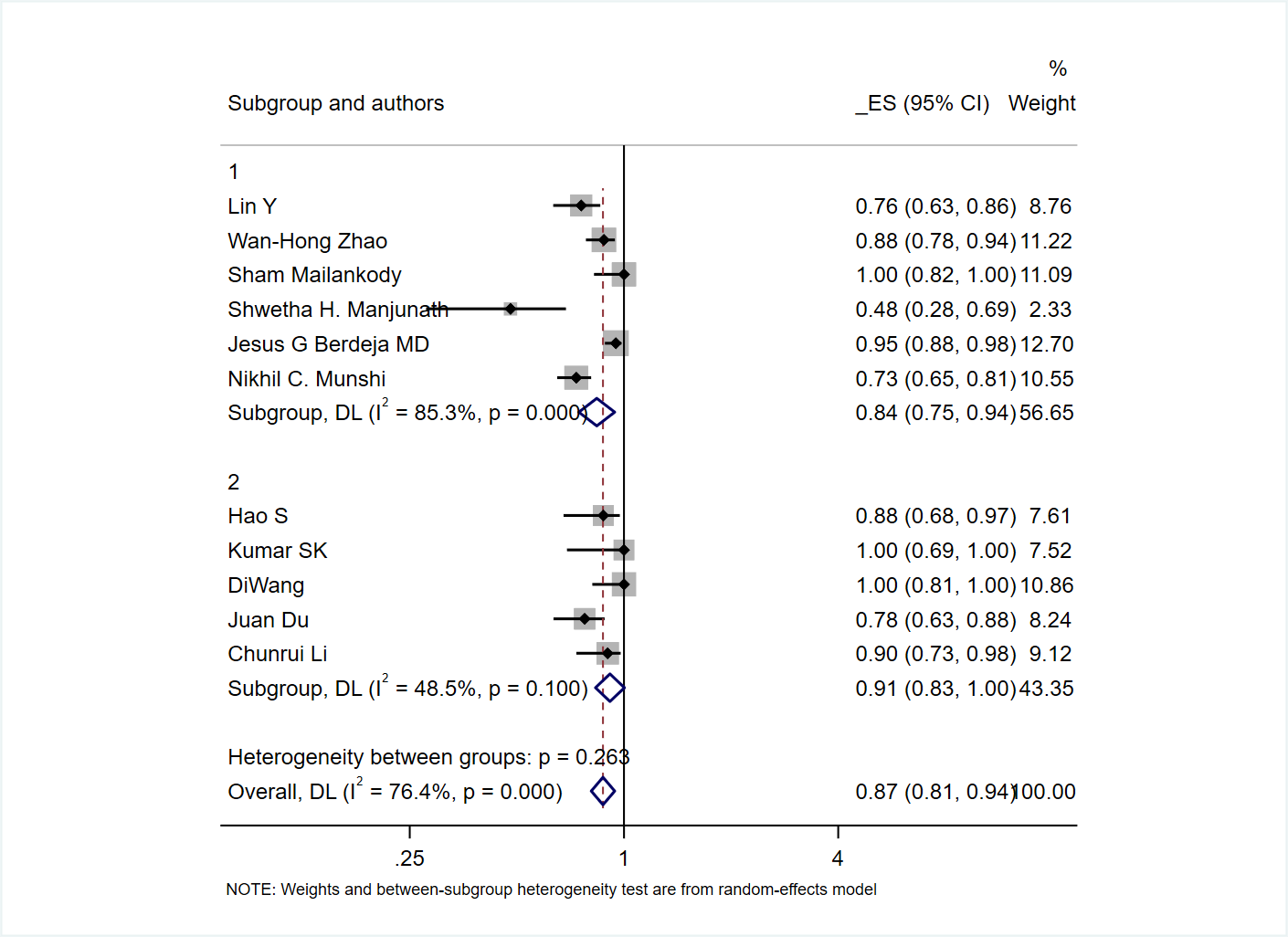


**j.**


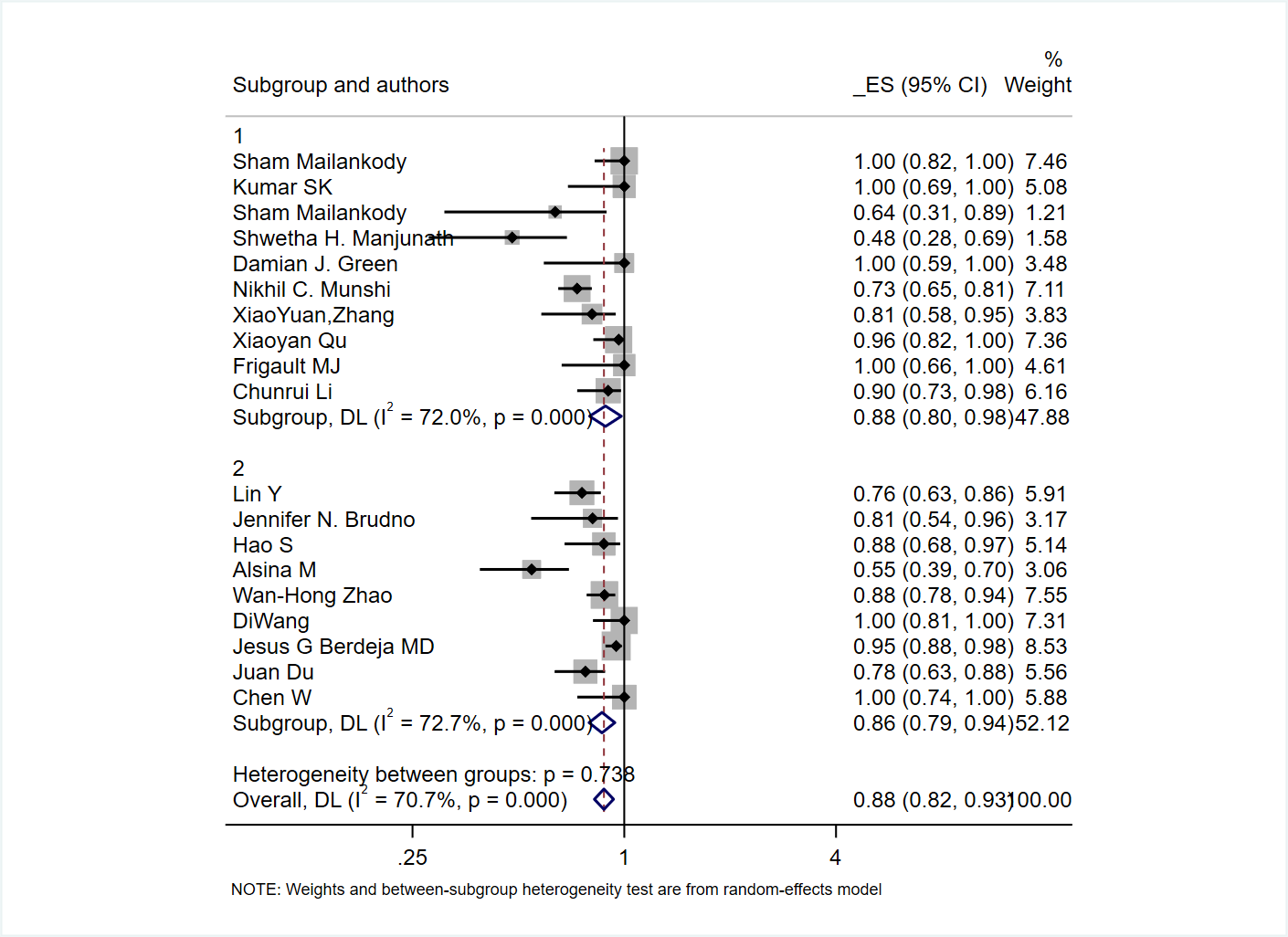


**k.**


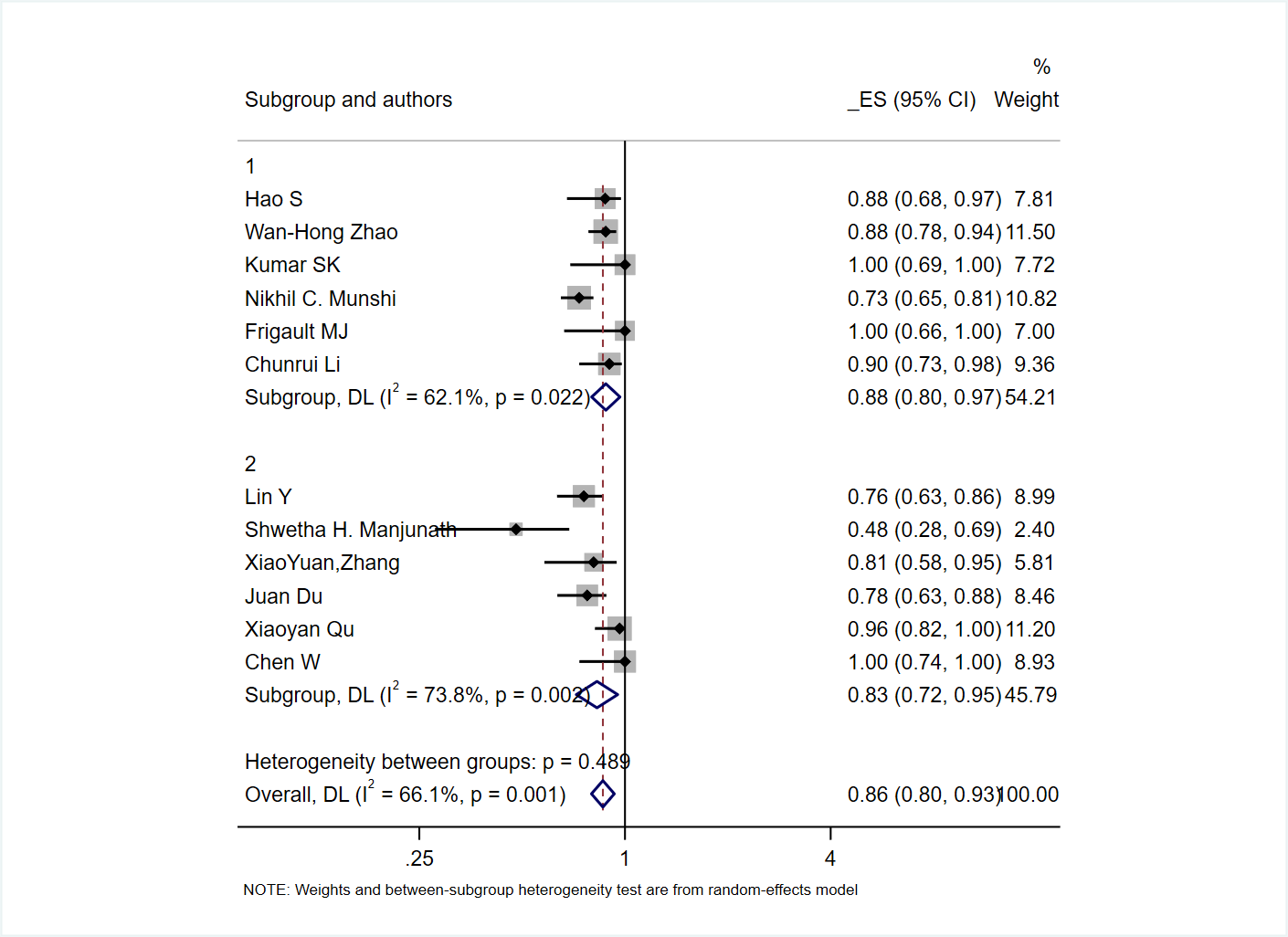


**l.**


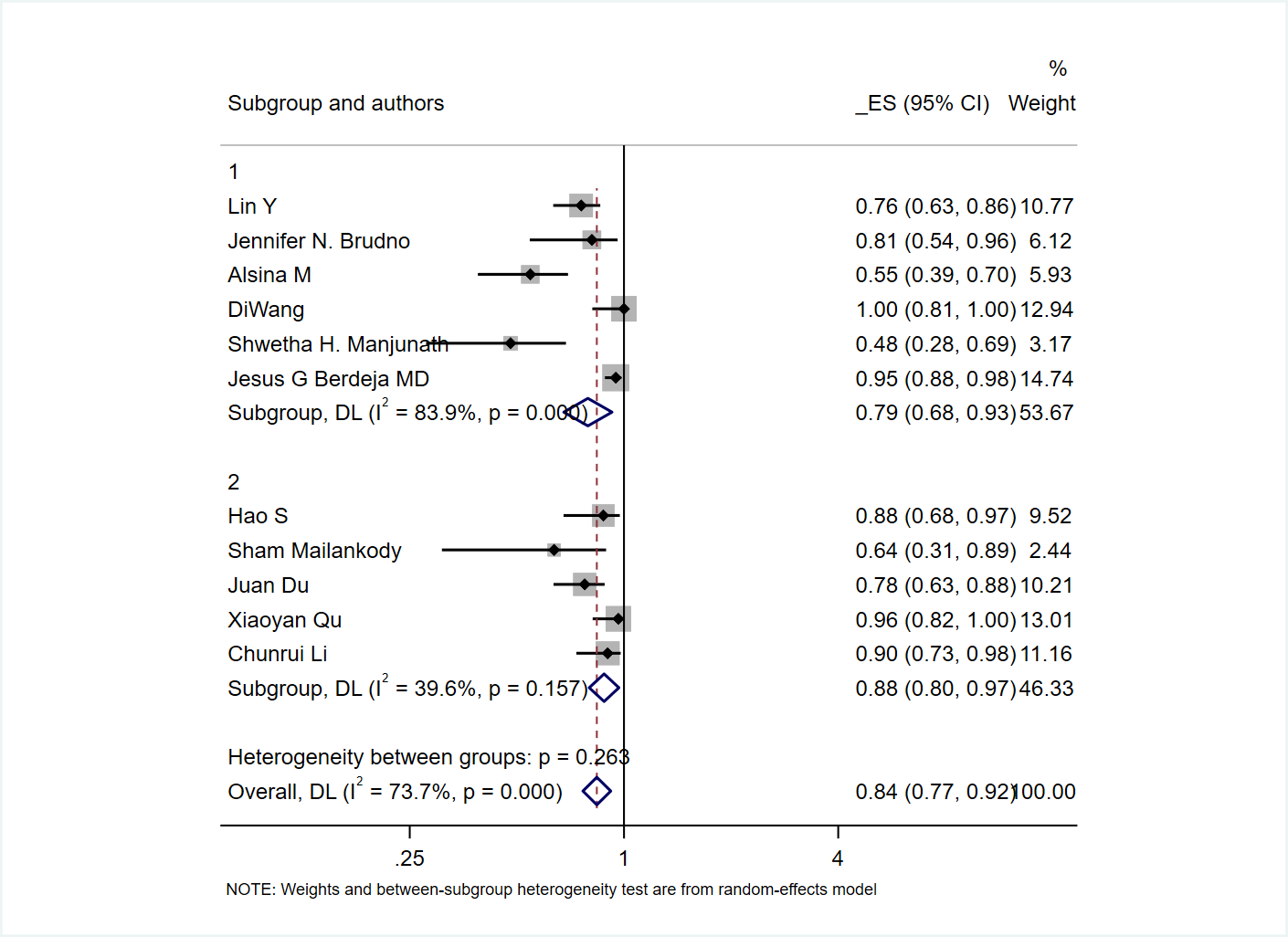


Supplemental Figure 6. Subgroup analyses of cytokine release syndrome (CRS) by (a) age (＜55 vs. ≥55 years), (b) patient disease status: proportion of ECOG≥3 level (＜25% vs. ≥25% ), (c) patient disease status: proportion of ISS≥3 level (＜28% vs. ≥28% ), (d) proportion of previous ASCT (＜75% vs. ≥75% ), (e) lines of prior treatment (＜8 vs. ≥8), (f) costimulatory molecule (4-1BB vs. others), (g) loading (Lentiviral vs. Retrovirus), (h) median time from diagnosis (＜4 vs. ≥4 years)，(i) proportion of high-risk cytogenetics (＜48% vs. ≥48%), (j) proportion of extramedullary disease (＜29% vs. ≥29% ), (k) proportion of mAb exposed (＜39% vs. ≥39% ) among RRMM.

**a.**


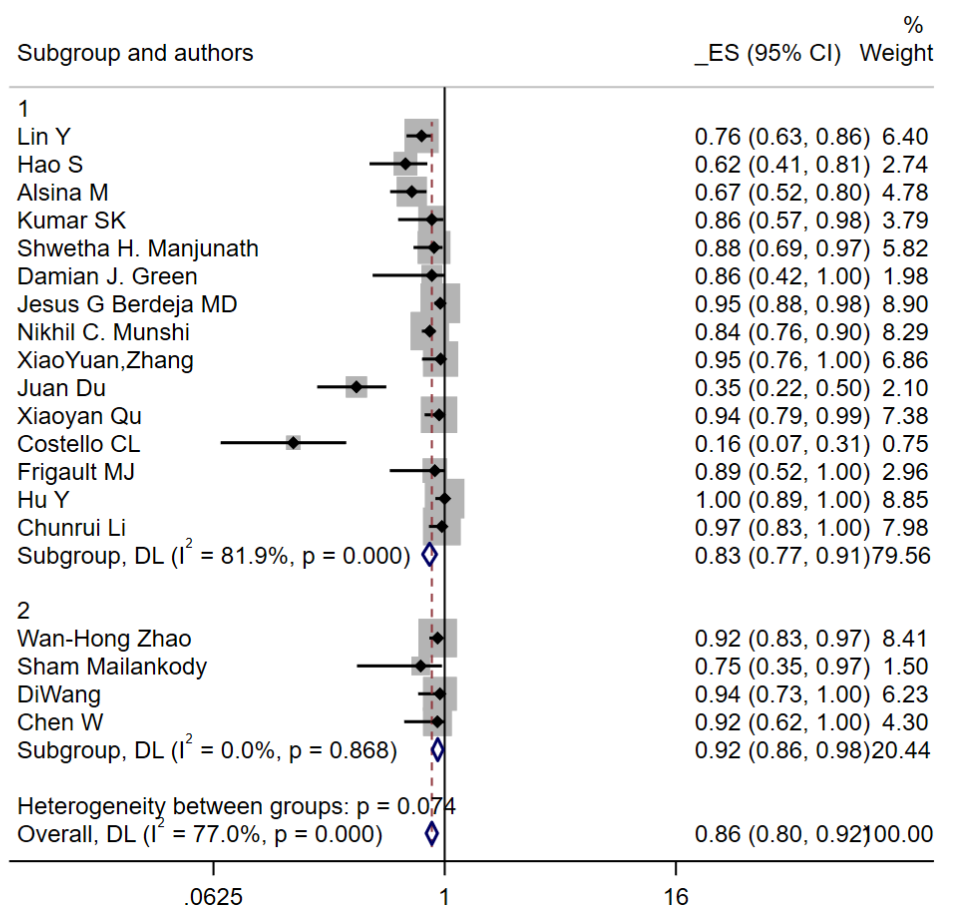


**b.**


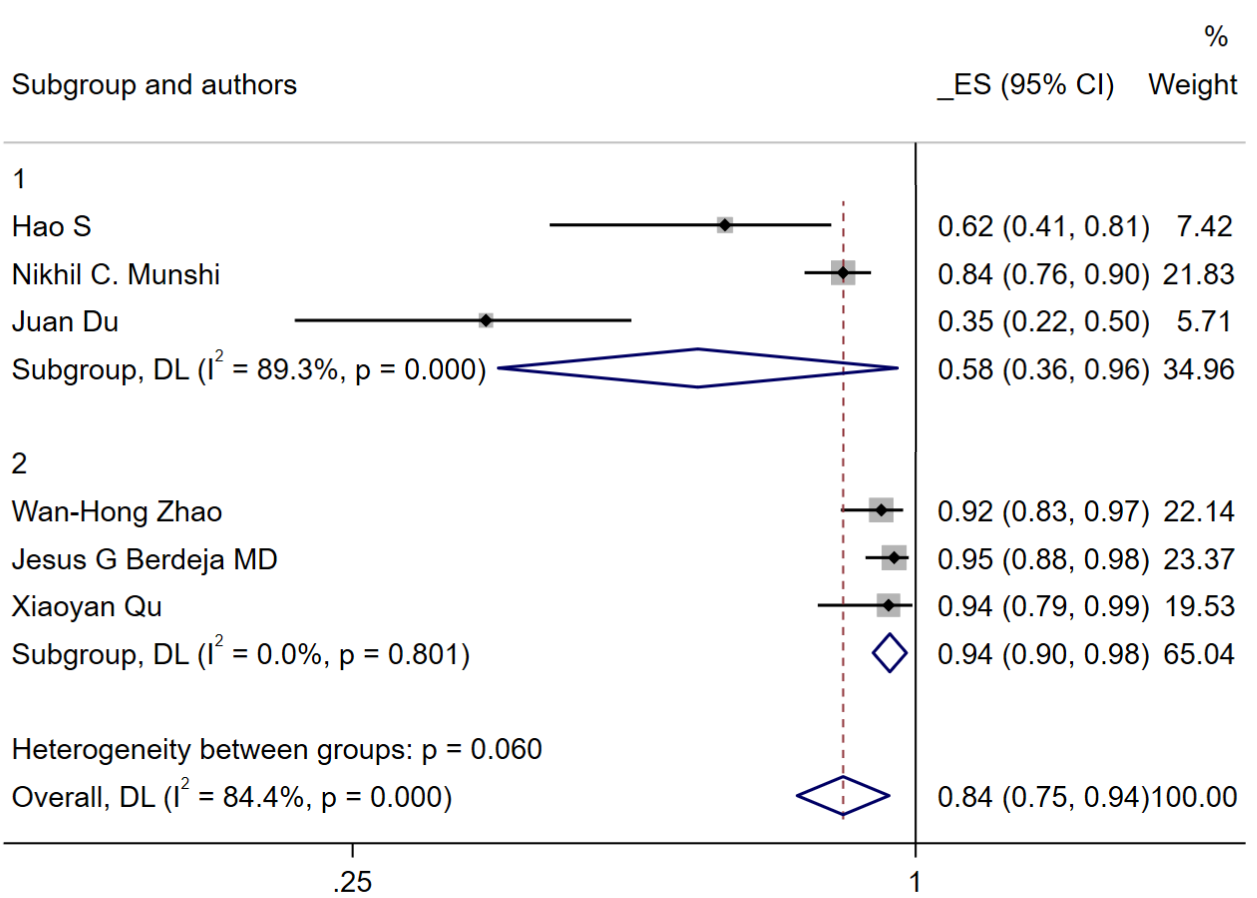


**c.**


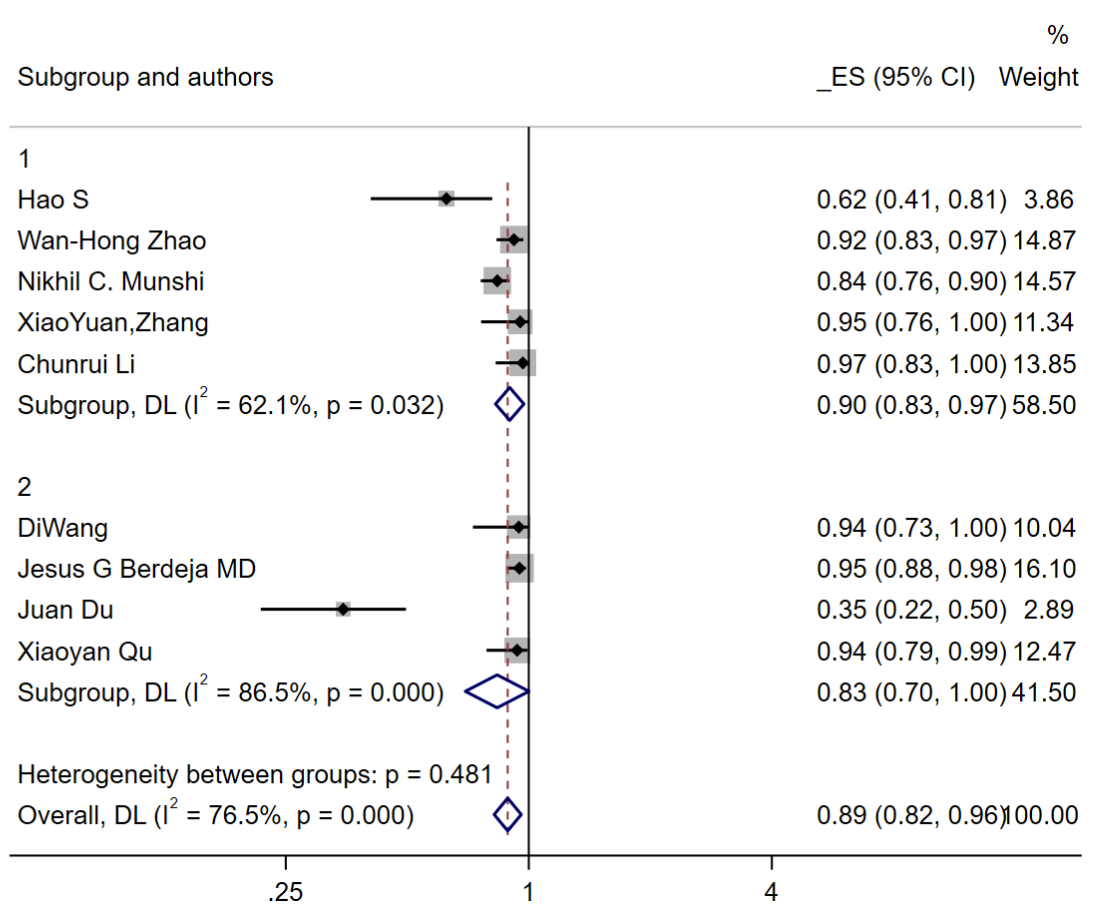


**d.**


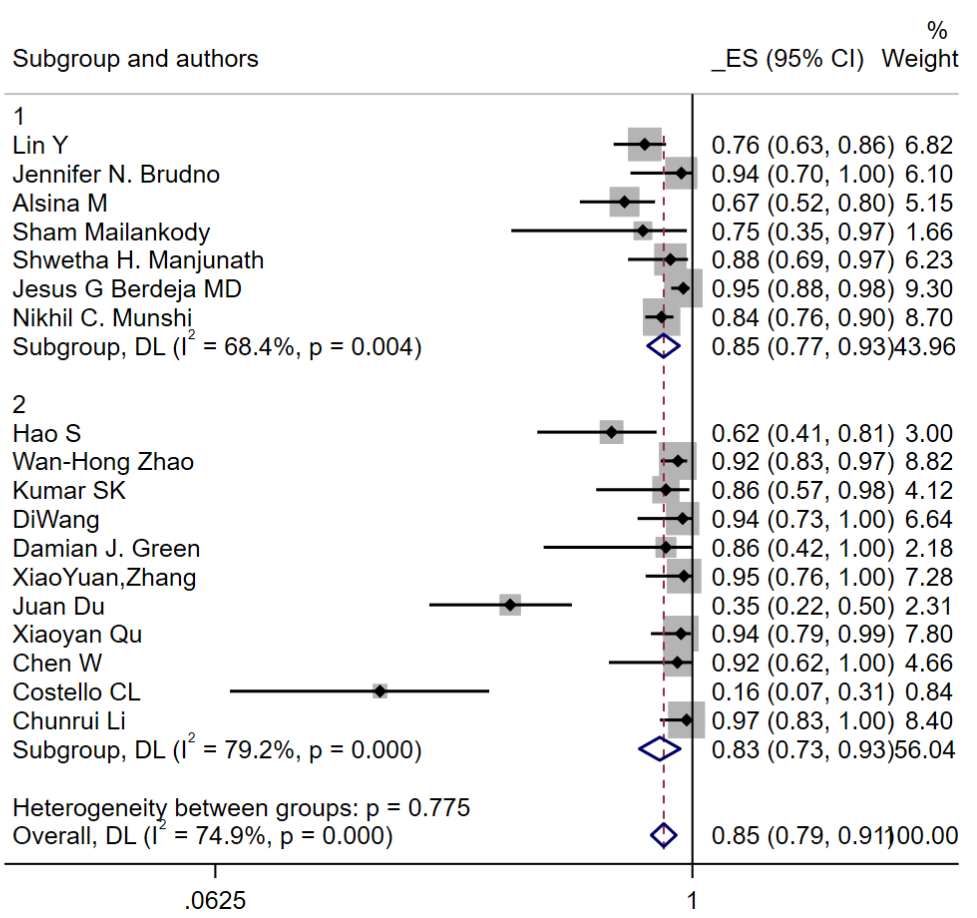


**e.**


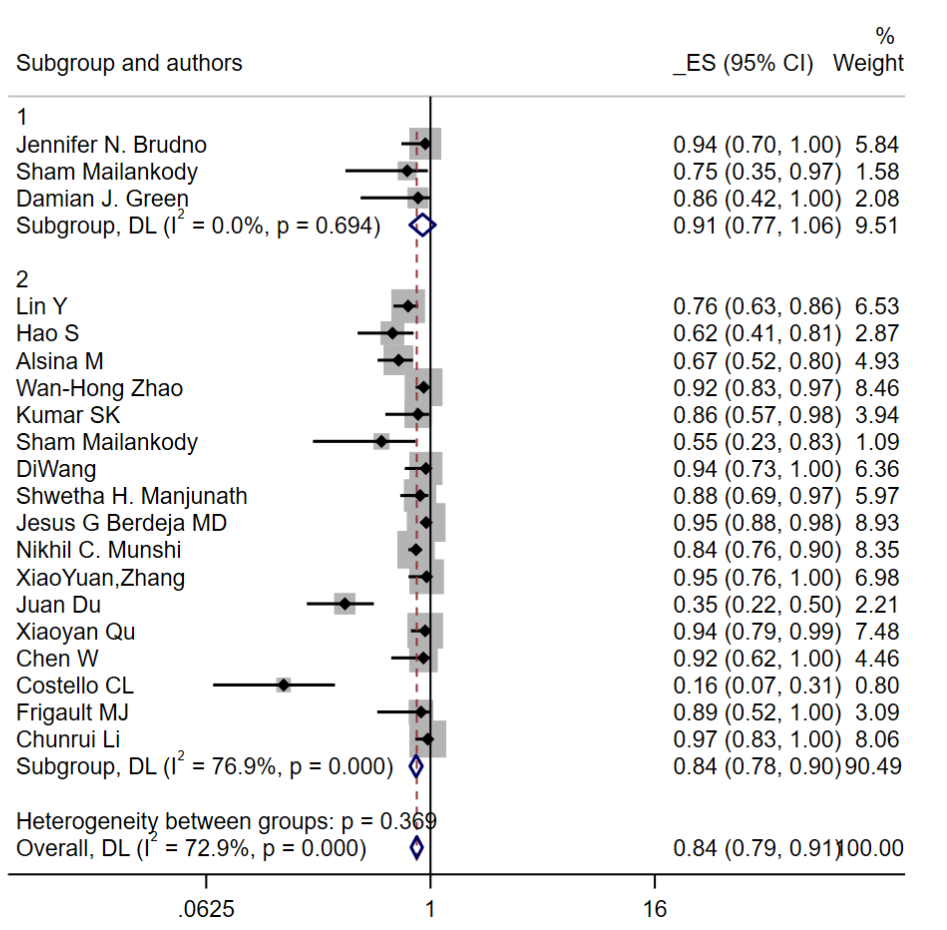


**f.**


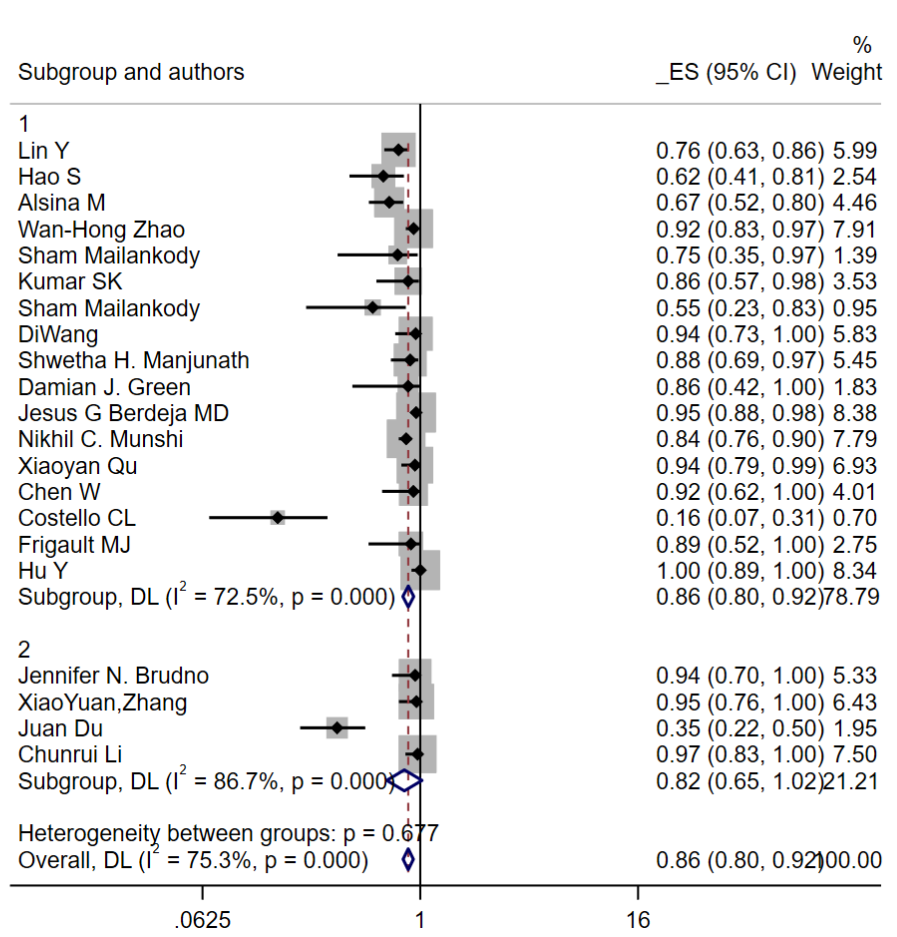


**g.**


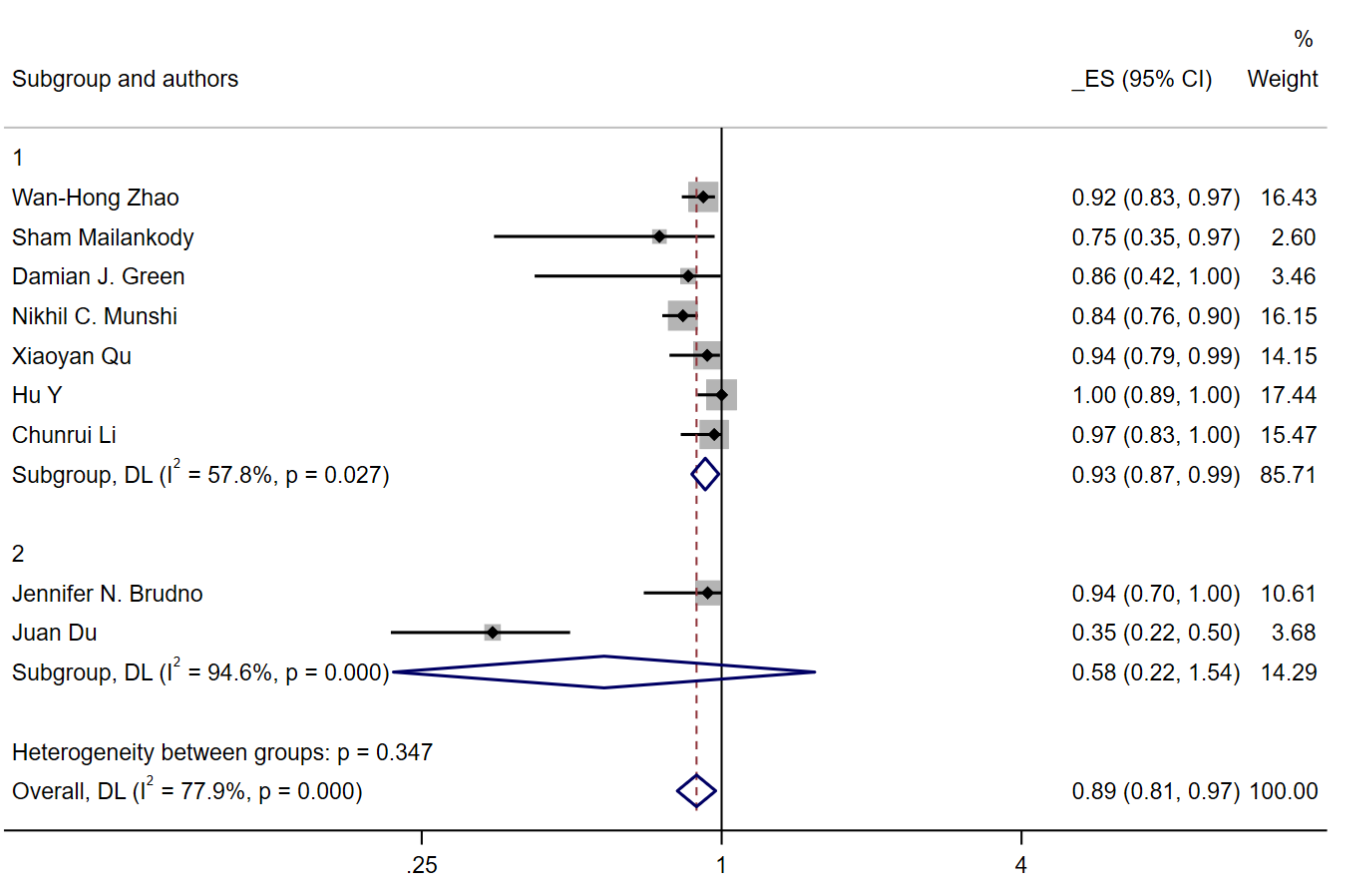


**h.**


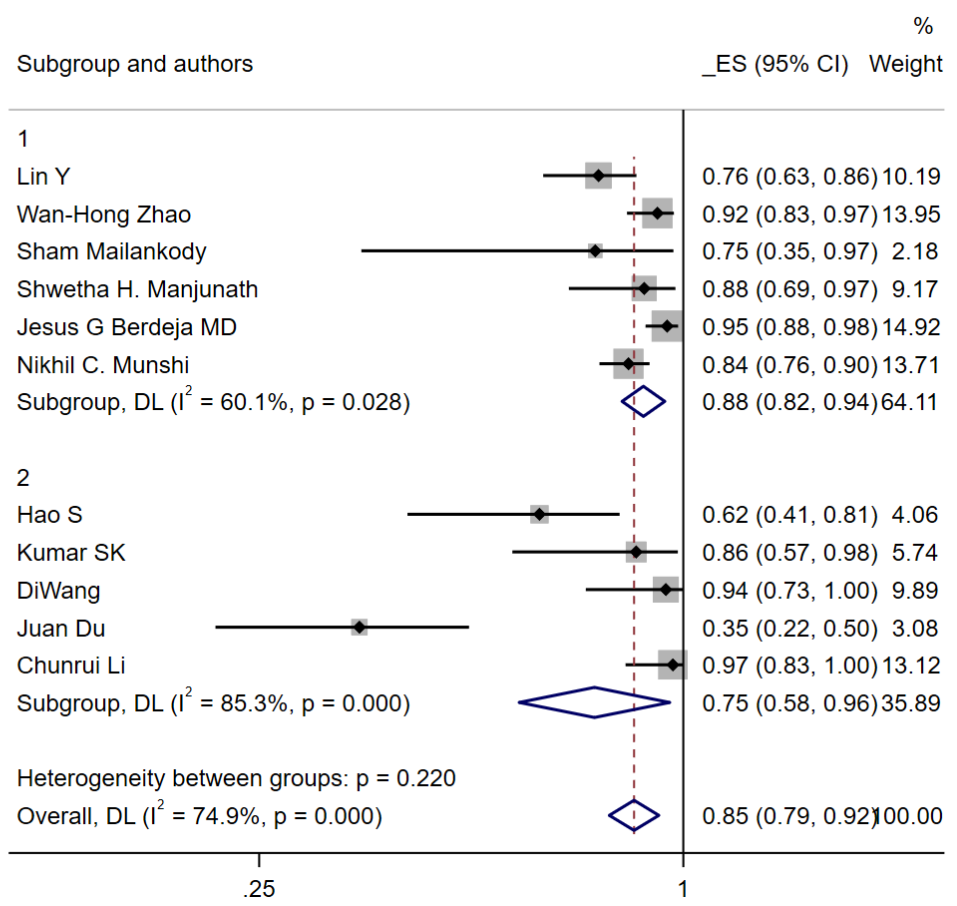


**i.**


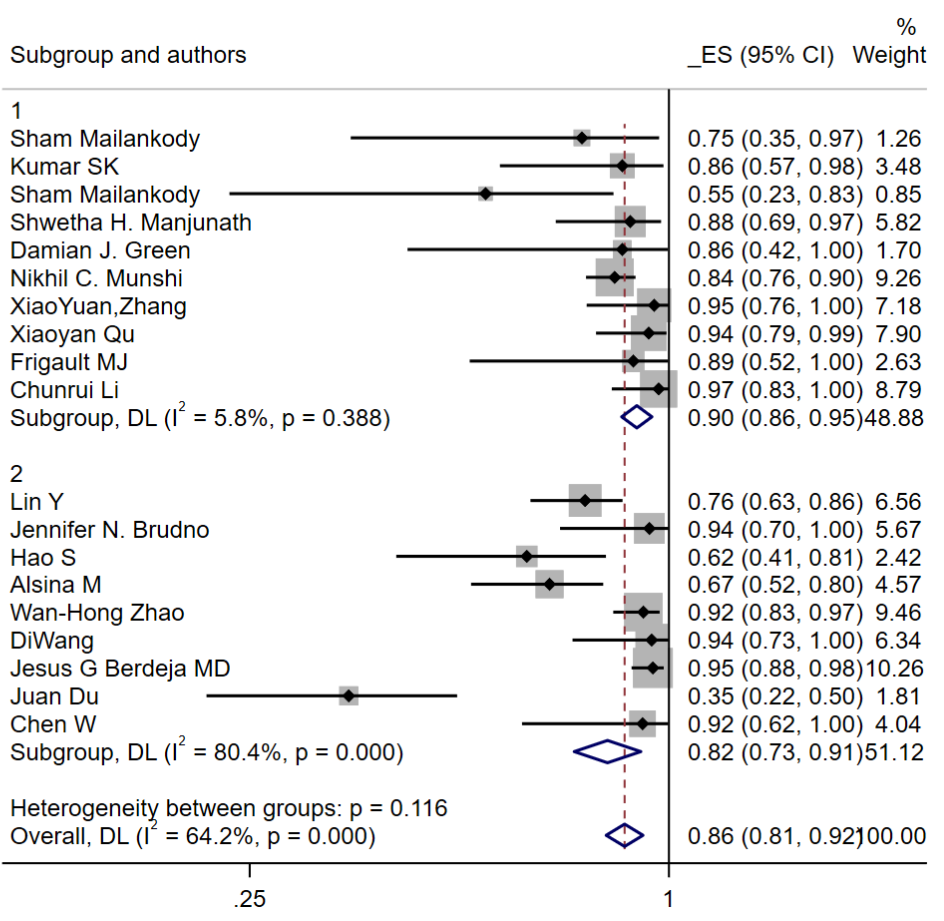


**j.**


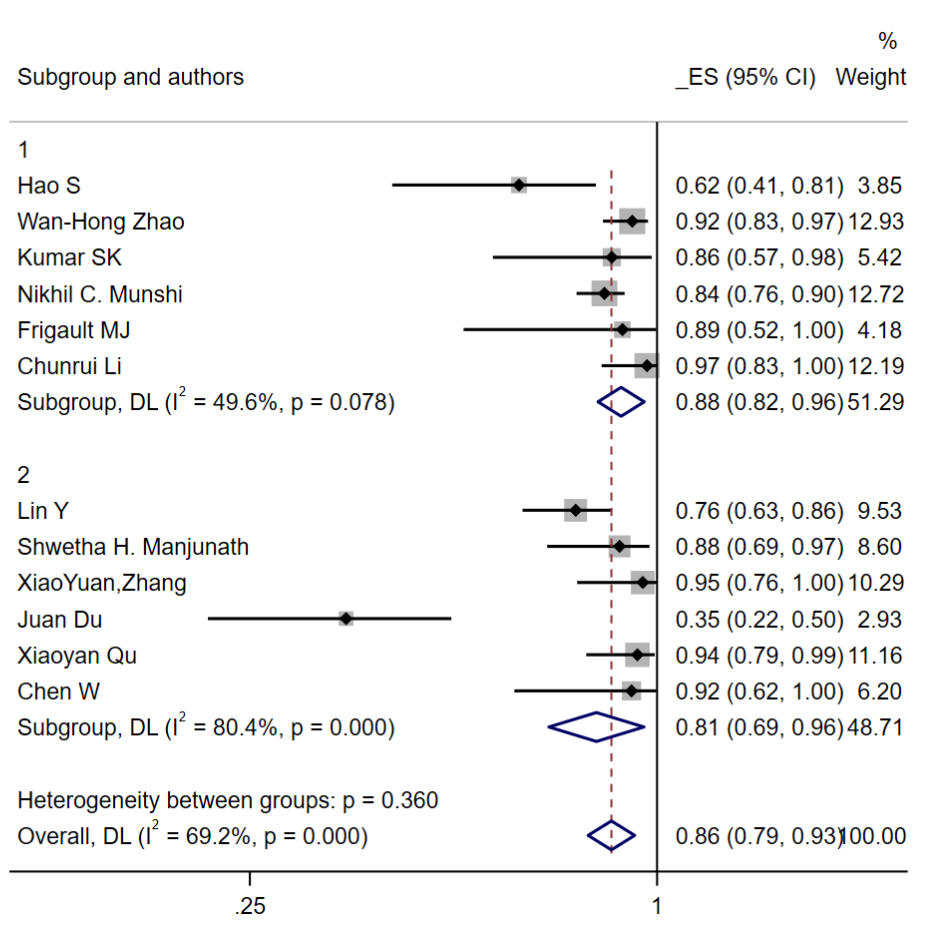


**k.**


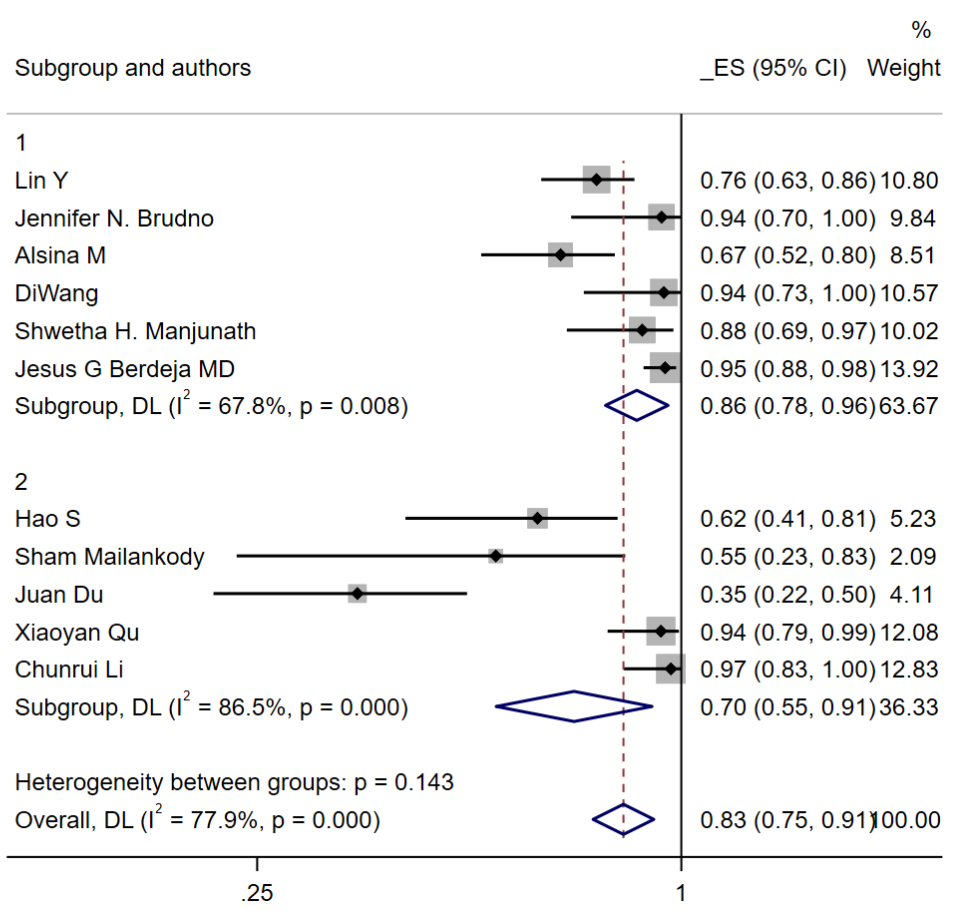

Supplement: Supplementary file 1 [file DataSheet1.docx]
